# Early evolution of the land plant circadian clock

**DOI:** 10.1111/nph.14487

**Published:** 2017-02-28

**Authors:** Anna‐Malin Linde, D. Magnus Eklund, Akane Kubota, Eric R. A. Pederson, Karl Holm, Niclas Gyllenstrand, Ryuichi Nishihama, Nils Cronberg, Tomoaki Muranaka, Tokitaka Oyama, Takayuki Kohchi, Ulf Lagercrantz

**Affiliations:** ^1^ Department of Plant Ecology and Evolution Evolutionary Biology Centre Uppsala University Norbyvägen 18D SE‐75236 Uppsala Sweden; ^2^ The Linnean Centre for Plant Biology in Uppsala Uppsala Sweden; ^3^ Graduate School of Biostudies Kyoto University Kyoto 606‐8502 Japan; ^4^ Department of Biology Lund University Ecology Building SE‐22362 Lund Sweden; ^5^ Graduate School of Science Kyoto University Kyoto 606‐8502 Japan

**Keywords:** bryophyte, circadian clock, *Marchantia polymorpha*, evolution, transcription factor

## Abstract

- While angiosperm clocks can be described as an intricate network of interlocked transcriptional feedback loops, clocks of green algae have been modelled as a loop of only two genes. To investigate the transition from a simple clock in algae to a complex one in angiosperms, we performed an inventory of circadian clock genes in bryophytes and charophytes. Additionally, we performed functional characterization of putative core clock genes in the liverwort *Marchantia polymorpha* and the hornwort *Anthoceros agrestis*.
- Phylogenetic construction was combined with studies of spatiotemporal expression patterns and analysis of *M. polymorpha* clock gene mutants.
- Homologues to core clock genes identified in Arabidopsis were found not only in bryophytes but also in charophytes, albeit in fewer copies. Circadian rhythms were detected for most identified genes in *M. polymorpha* and *A. agrestis*, and mutant analysis supports a role for putative clock genes in *M. polymorpha*.
- Our data are in line with a recent hypothesis that adaptation to terrestrial life occurred earlier than previously expected in the evolutionary history of charophyte algae. Both gene duplication and acquisition of new genes was important in the evolution of the plant circadian clock, but gene loss has also contributed to shaping the clock of bryophytes.

## Introduction

Adaptation to changing environments is critical to all life. Some of these changes are predictable, such as day–night cycles and the ever‐changing seasons. Accordingly, organisms from all kingdoms of life have developed mechanisms to anticipate such predictable changes. Intrinsic clocks that generate circadian rhythms are present in most organisms, from cyanobacteria to land plants and animals. Although the overall architecture is generally conserved, the key genes involved are generally not, suggesting multiple independent origins of circadian clocks (Dunlap, 1999; Young & Kay, 2001; McClung, 2013).

The circadian clock is a self‐sustaining oscillator and the *c*. 24 h rhythm results mainly from transcriptional and translational feedback loops (Harmer, 2009). The clock must interact with a fluctuating environment and needs to be adjusted daily. The major environmental cues for this entrainment are light and temperature (Johnson *et al*., 2003). The clock also has to cope with unpredictable variations in sunlight and temperature and is generally thought to have evolved towards a more complex and thereby flexible and robust architecture (Rand *et al*., 2004; Tsai *et al*., 2008).

Circadian regulation is important in the control of a number of diverse processes. In vascular plants, well‐studied examples include stomatal opening, leaf movement, growth, metabolism, induction of flowering and response to stress (Greenham & McClung, 2015). Correspondingly, thousands of genes are under circadian control (Covington *et al*., 2008; Michael *et al*., 2008). Disruption of the circadian clock has also been shown to confer fitness costs, implicating its importance (Yerushalmi & Green, 2009).

The plant circadian clock has been intensely studied in a few model species, mainly the angiosperm *Arabidopsis thaliana*, and the green algae *Chlamydomonas reinhardtii* and *Ostreococcus tauri*. Recent models of the Arabidopsis clock describe it as an intricate network of interlocked transcriptional feedback loops (Pokhilko *et al*., 2013; Fogelmark & Troein, 2014; De Caluwé *et al*., 2016). The main components of those models include a set of single MYB domain transcription factors, a family of PSEUDO‐RESPONSE REGULATORs (PRRs), and a few plant‐specific genes with unknown biochemical function (Table 1).

**Table 1:** Clock gene homologues in *Chlamydomonas reinhardtii* (Cr), *Ostreococcus tauri* (Ot), *Klebsormidium flaccidum* (Kf), *Marchantia polymorpha* (Mp), *Anthoceros agrestis* (Aa), *Physcomitrella patens* (Pp), *Selaginella moellendorffii* (Sm), *Picea abies* (Pa), *Oryza sativa* (Os) and *Arabidopsis thaliana* (At)

Species^a^	RVE		PRR		ELF3	ELF4	LUX	GI	ZTL
	CCA1‐clade	LCL‐clade	TOC1‐clade	PRR‐clade					
Cr	1^b^	2^c^	0	1^e^	2^e^	0	0		
Ot	1^b^	1^c^	0	0	0	0	0		
Kf	1	1	1	1	1	2	2	0^d^	1
Mp	0	1	1	1	1	1	1	1	1
Aa	1	1	0	1	1	2	1	1	1
Pp	2	3	0	4	3	1	3	0	0
Sm	0	1	1	2	1	2	1	1	1
Pa	3	0	1	2	0	5	2	1	1
Os	1	6	1	4	2	3	1	1	3
At	6	5	1	4	2	5	2	1	3

a Corresponding locus names and accession numbers are found in Supporting Information Table S1.

b These genes could not be assigned to either the CCA1/LHY or the LCL clade.

c These genes could not be assigned to either the PRR or the TOC1 clade.

d GI was not found in *K. flaccidum*, but was identified in other charophytes.

e Potential homologue.

The components of the Arabidopsis clock can be classified according to their phase of expression. The morning‐phased genes *CIRCADIAN CLOCK‐ASSOCIATED 1 (CCA1)* and *LATE ELONGATED HYPOCOTYL (LHY)* are two MYB‐like transcription factors that function mainly as repressors of day‐ and evening‐phased genes (Wang *et al*., 1997; Schaffer *et al*., 1998; Mizoguchi *et al*., 2002; Fogelmark & Troein, 2014; Kamioka *et al*., 2016). They bind to a motif coined the evening element (EE; Harmer *et al*., 2000) that is present in the promoters of several day‐ and evening‐phased genes, as well as in a large number of putative output genes.

The evening‐phased component includes three proteins that form the evening complex (EC). The complex contains the MYB transcription factor LUX ARRHYTHMO (LUX), and the two proteins EARLY FLOWERING 3 (ELF3)*,* and ELF4 (Hicks *et al*., 2001; Doyle *et al*., 2002; Hazen *et al*., 2005; Nusinow *et al*., 2011). The EC functions as a repressor of at least two *PRR* genes as well as *LUX* itself (Nusinow *et al*., 2011; Chow *et al*., 2012).

The family of *PRR* genes comprise five members in Arabidopsis: *PRR1*,* PRR3*,* PRR5*,* PRR7*, and *PRR9*. *PRR1* is also known as *TIMING OF CAB EXPRESSION 1* (*TOC1*), which, together with CCA1, constituted the first conceptual model of the Arabidopsis clock (Alabadí *et al*., 2001). The expression of *PRR* genes ranges from morning to evening, with *PRR9* peaking in the morning, *PRR5* and *PRR7* around noon, and *PRR3* and *TOC1* around dusk (Matsushika *et al*., 2000). PRR proteins are in recent models incorporated as transcriptional repressors of *CCA1/LHY* and other *PRR* genes (Fogelmark & Troein, 2014).

The proteins described are reported to function as repressors. However, recently a family of MYB transcription factors related to CCA1/LHY was identified (Rawat *et al*., 2011; Hsu *et al*., 2013), some of which were reported to work as activators in the circadian clock. These *REVEILLE* (*RVE*) genes are transcribed mainly in the morning, but the protein amounts of at least some members peak in the afternoon. RVE8 and probably RVE4 and RVE6 bind to the same EE as CCA1/LHY and induce evening‐phased genes. Conversely, one or more PRR proteins repress *RVE8* and possibly other *RVE* genes.

Additionally, ZEITLUPE (ZTL), an F‐box protein, and *GIGANTEA* (*GI*), encoding a large protein with unclear biochemical function, are implicated to function in the circadian clock (Fowler *et al*., 1999; Somers *et al*., 2000). GI interacts with ZTL which, in turn, regulates the stability of at least TOC1 and PRR5 (Más *et al*., 2003; Kim *et al*., 2007). The activity of *GI* is under clock control, including repression by EC and CCA1/LHY and probably also activation by RVE8 (Berns *et al*., 2014). GI is also important for flowering time regulation through its interaction with the ZTL paralogue FLAVIN‐BINDING, KELCH REPEAT, F‐BOX1 (FKF1; Sawa *et al*., 2007). The GI–FKF1 complex controls the degradation of CYCLING DOF FACTOR proteins, which in turn are repressors of the flowering time gene *CONSTANS* (Fornara *et al*., 2009).

The clocks in green algae differ considerably from the angiosperm clock (Matsuo *et al*., 2008; Corellou *et al*., 2009). In both *C. reinhardtii* and *O. tauri*, putative homologues to *CCA1* and *TOC1* have been identified, however, no obvious *ELF3*,* ELF4*,* GI* or *ZTL* homologues could be found (Matsuo *et al*., 2008; Corellou *et al*., 2009). Even though the identification of clock components in these algae might be incomplete, comparatively simple models can be built that reproduce the dynamics of their circadian clocks. For example, the *O. tauri* clock was successfully modelled as a feedback loop between a *CCA1* and a *TOC1* homologue (Troein *et al*., 2011).

Comparisons of circadian clock genes in green algae and angiosperms suggest that additional genes have been recruited during plant evolution, resulting in successively more complex clocks with multiple feedback loops. However, when and how these genes were recruited is currently unknown. Land plants form a monophyletic group, which nests within a freshwater Charophycean algal clade, implying that land plants evolved from an ancestral freshwater or terrestrial alga (Bowman, 2013; and references therein; Harholt *et al*., 2016). Liverworts, mosses and hornworts are gametophyte‐dominant land plants collectively known as bryophytes, and comprise the closest extant relatives to the first embryophytic land plants (Shaw *et al*., 2011). Their phylogenetic position makes them crucial for studying the evolution of biological processes from freshwater plants to land plants, and from relatively simple basal land plants to more complex forms. A few studies investigating clock genes in the moss *Physcomitrella patens* have concluded that although multiple *PRR* genes and homologues to *CCA1* and the EC genes were identified, orthologues to important clock genes such as *GI*,* TOC1* and *ZTL* were not (Okada *et al*., 2009; Holm *et al*., 2010; Satbhai *et al*., 2011).

Even though the circadian clock has been studied in a few plant model species over the last decades, little is known of clock function in the bryophytes. Because of their key phylogenetic position, bryophytes have the potential to reveal the mechanisms regulating the ancestral clock present in the first plants that colonized land. This study gives a comparative overview of charophyte and bryophyte clock gene families, spanning over all three major clades of bryophytes – mosses, liverworts and hornworts – and suggests that most components present in angiosperm circadian clocks were already recruited in the ancestors of land plants (the charophytes). Gene duplication has generally resulted in an increased complexity of the plant circadian clock, but independent gene losses in various lineages have shaped the architecture of the individual clock networks.

## Materials and Methods

### Plant material and growth conditions

*Marchantia polymorpha* ssp. *ruderalis* from Uppsala, Sweden, referred to as Upp, was grown aseptically on agar solidified Gamborg's B5 (Gamborg *et al*., 1968; PhytoTechnology Laboratories, Lenexa, KS, USA), pH 5.5. Strains Takaragaike (Tak) ‐1 and Tak‐2 were grown in a similar way in the Kyoto laboratory, and used as indicated in the text. Plants were grown under cool white fluorescent light (50–60 μmol photons m^−2^ s^−1^) in 16 : 8 h, light : dark cycles at 20°C (Uppsala), or in continuous light at 22°C (Kyoto).

### RNA isolation and quantitative reverse transcription polymerase chain reaction (qRT‐PCR)

*Marchantia polymorpha* (Upp) and *Anthoceros agrestis* were grown on half‐strength Gamborg's B5 with 1% sucrose in growth cabinets (MLR‐350; Sanyo, Osaka, Japan) with fluorescent tubes producing 40–50 μmol m^−2^ s^−1,^ red : far red ≈ 7.0 at 18°C. Plants were entrained in neutral day (ND; 12 : 12 h, light : dark cycles), and then transferred to constant darkness (DD), constant light (LL) or ND. Total RNA extraction and qRT‐PCR were performed as previously described (Holm *et al*., 2010), with PCR primers listed in Supporting Information Table S2. Primers targeting Mpβ‐*tubulin2* (Mp*TUB2*; Mapoly0158s0010.1; Buschmann *et al*., 2016), Mp*ACTIN* (Mp*ACT*) and Mp*ADENINE PHOSPHORIBOSYL TRANSFERASE* were utilized for normalization of gene expression (Saint‐Marcoux *et al*., 2015). Corresponding primers for *A. agrestis* targeted Aa*ACT* and Aa*TUB*. Normalization of gene expression was performed using analysis of covariance (ANCOVA) in R (v.3.0.2; R Core Team, 2016), where the response variable was the Ct value of the target gene and explanatory variables Ct values from the reference genes plus time. Residuals from the ANCOVA analyses were used for plotting and analysis of rhythmic expression using JTK_CYCLE (Hughes *et al*., 2010).

RNA isolation and gene expression analyses in *M. polymorpha* (Tak) were performed as described previously (Kubota *et al*., 2014), with at least two technical replicates for each cDNA sample. Primers are shown in Table S2. Expression data were normalized against *ELONGATION FACTOR 1*α (Mp*EF1*). Relative expression values for each gene and sample were then calculated as the average of three biological replicates.

### Cloning and construction of plasmids

To generate targeting vectors for Mp*PRR*, Mp*RVE*, and Mp*TOC1*,* c*. 3 kb flanking regions were PCR‐amplified from Tak‐1 genomic DNA. Primers are listed in Table S2. The PCR‐amplified fragments were cloned into the *Pac*I and *Asc*I sites of pJHY‐TMp1 (Ishizaki *et al*., 2013), using the In‐Fusion HD cloning kit (Clontech, Kusatsu, Japan). The resulting plasmids were introduced into sporelings derived from crosses between Tak‐1 and Tak‐2 (Ishizaki *et al*., 2008). Screening for correctly targeted lines was performed by genomic PCR as described previously (Ishizaki *et al*., 2013; see later Figs S8–S10). For the complementation test of Mp*rve* ^*ko*^ and Mp*prr* ^*ko*^, genomic fragments including entire regions of Mp*RVE* or Mp*PRR* were PCR‐amplified from Tak‐1 genomic DNA and subsequently cloned into pENTR/D‐TOPO (Life Technologies, Yokohama, Japan). Fragments were subcloned into pMpGWB301 (Ishizaki *et al*., 2015) in order to generate binary plasmids harbouring Mp*RVE*_*pro*_ *:g*Mp*RVE* or Mp*PRR*_*pro*_ *:g*Mp*PRR*. The resulting plasmids were introduced into corresponding knockout mutants, Mp*rve* ^*ko*^ or Mp*prr* ^*ko*^, as described previously (Kubota *et al*., 2013).

Binary promoter:*LUC* plasmids were built by PCR‐amplifying regulatory regions from genomic DNA (Upp) extracted using the DNeasy Plant Mini Kit (Qiagen). Fragments were subcloned into pMpGWB431 (Nakajima *et al*., 2010; Ishizaki *et al*., 2015), via pENTR/D‐TOPO (Invitrogen). These plasmids were transformed into sporelings (Upp) as previously described (Ishizaki *et al*., 2008). To generate the *35S*_*pro*_ *:LUC* construct, a *Hin*dIII/*Sma*I fragment from pBI221 (Takara Bio, Kusatsu, Japan) was blunt‐ended and inserted into the *Dra*I/*Eco*RV site of pENTR1a (Life technologies). This fragment was then subcloned into pMpGWB331 (Ishizaki *et al*., 2015). *35S*_*pro*_ *:LUC* was introduced into Tak‐1, Mp*toc1* ^*ko*^ *,* Mp*rve* ^*ko*^ or Mp*prr* ^*ko*^ as described previously (Kubota *et al*., 2013).

### Luciferase expression analysis

Gemmalings entrained in ND at 20°C on medium containing 1% sucrose (Uppsala) or 22°C (Kyoto) for 4–7 d were sprayed with a 1 mM solution of D‐Luciferin (Pierce D‐Luciferin; Fisher Scientific, Göteborg, Sweden). After an additional 1–2 d in ND, bioluminescence imaging was performed. In Kyoto, a dish monitoring system with photomultiplier tubes (R329P; Hamamatsu Photonics K.K., Hamatsu City, Japan) was used, as described in Miwa *et al*. (2006). To reduce fluorescent signals form Chl, a short‐pass filter (VS0630; Asahi Spectra Co. Ltd, Tokyo, Japan) was used. Each dish was subjected to 30s measurement of bioluminescence every 20 min. In Uppsala, plates were imaged with an ImagEM charge‐coupled device (CCD) camera (Hamamatsu Photonics K.K.) in a light contained box equipped with blue and red LEDs. Light intensity was set to equal amounts of blue and red light with a total intensity of 50 μmol m^−2^ s^−1^. Ten‐minute exposures were taken every hour with the light turned off starting 3 min before exposure during light periods. Intensity data were extracted with ImageJ (Abramoff *et al*., 2004), and analysed with Spectrum Resampling (Costa *et al*., 2013). For 12 h T‐cycle experiments, light intensity was reduced to 5 μmol m^−2^ s^−1^ to increase the possibility of revealing frequency demultiplication.

To visualize spatial *LUC* expression, patterns plants were sprayed with 1 mM D‐Luciferin and incubated for 12 h in continuous light. Plants were then placed in darkness for 5 min, and imaged with the CCD camera for 10 min.

## Results

### Identification of putative bryophyte and charophyte clock genes

To trace the origin of the plant circadian clock, we performed an inventory of homologues to known plant circadian clock genes in available bryophyte and charophyte genomes (Table 1; Methods S1). To identify orthologues and examine the evolutionary relationship of plant circadian clock genes, phylogenetic trees were constructed based on alignments shown in Fig. S1. The results are presented below on a gene family basis.

#### REVEILLE

The 11 members of the *RVE* family in Arabidopsis – *CCA1*,* LHY* and nine *RVE* genes – all encode proteins with a single highly conserved MYB/SANT domain (SHAQKYF class; Rawat *et al*., 2011; Farinas & Mas, 2011). Two subgroups have been named in this family, one comprising *CCA1*,* LHY*,* RVE1*,* 2*,* 7* and *RVE7‐like* and the other *RVE3*,* 4*,* 5*,* 6* and *8*. The second group, referred to as the *LCL* subfamily, shares an additional conserved region outside the MYB domain (Farinas & Mas, 2011).

**Figure 1:**
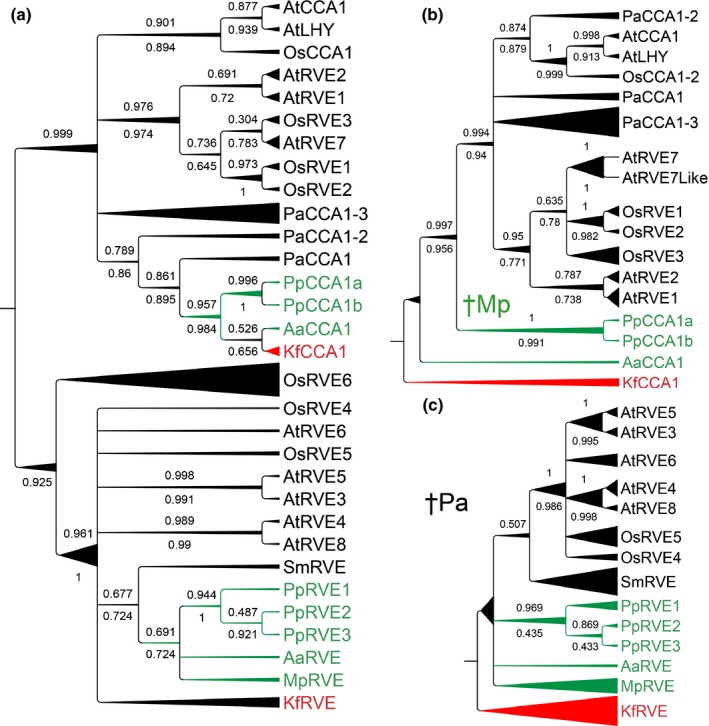
Inferred phylogeny of homologues to the CCA/LHY/RVE gene family. Separate trees were constructed from the complete alignment (a), the *CCA1* subfamily (b), and the LCL subfamily (c). Trees were constructed using MrBayes and PhyML on an amino acid alignment of proteins retrieved from *Arabidopsis thaliana* (At), *Oryza sativa* (Os), *Picea abies* (Pa), *Selaginella moellendorffii* (Sm), *Physcomitrella patens* (Pp), *Marchantia polymorpha* (Mp), *Anthoceros agrestis* (Aa) and *Klebsormidium flaccidum* (Kf). Bayesian trees are shown with posterior probabilities (above) and bootstrap proportions from PhyML analysis (below) for each node. Nodes with conflicting support from the two methods were collapsed. Branch length is relative to the thickness of individual branches: the shortest branches have a straight line and the longest are increasingly triangular. †Mp indicates loss of a CCA1/LHY homologue in Mp (as well as all surveyed liverworts); †Pa indicates loss of LCL‐like genes in Pa. The MYB/SANT domain includes, in the LCL group, a SHAQKYF‐version of the SHAQKYF‐motif, while the CCA1 subclade shares SHAQKFF.

As previously reported, the green algae *C. reinhardtii* and *O. tauri* contain single homologues that are similar to genes in the CCA1/LHY clade (Matsuo *et al*., 2008; Corellou *et al*., 2009). However, in the charophyte *Klebsormidium flaccidum* two homologues were found, one in the CCA1/LHY clade (Kf*CCA1*) and the other in the LCL clade (Kf*RVE*; Fig. 1; Table 1). Likewise, *A. agrestis* and *P. patens* contain genes from both groups (Fig. 1; Table 1).

In the liverwort *M. polymorpha* only one homologue was identified, Mp*RVE*, which clustered with the *LCL* clade in a phylogenetic analysis (Fig. 1; Table 1), suggesting that *M. polymorpha* has lost the *CCA1*/*LHY*‐type gene. Based on data from the oneKP database, the presence of one *LCL* gene and no *CCA1*/*LHY* gene is shared among all 28 deposited liverwort species, while mosses and hornworts contain both *LCL*‐like and *CCA1*/*LHY*‐like homologues.

#### PSEUDO‐RESPONSE REGULATORS

PSEUDO‐RESPONSE REGULATOR proteins contain a response regulator receiver (REC) domain and a CONSTANS, CONSTANS‐like and TOC1 (CCT) domain (Strayer *et al*., 2000). This family consists of five members in Arabidopsis: *TOC1*/*PRR1*,* PRR3*,* PRR5*,* PRR7* and *PRR9* (Matsushika *et al*., 2000).

**Figure 2:**
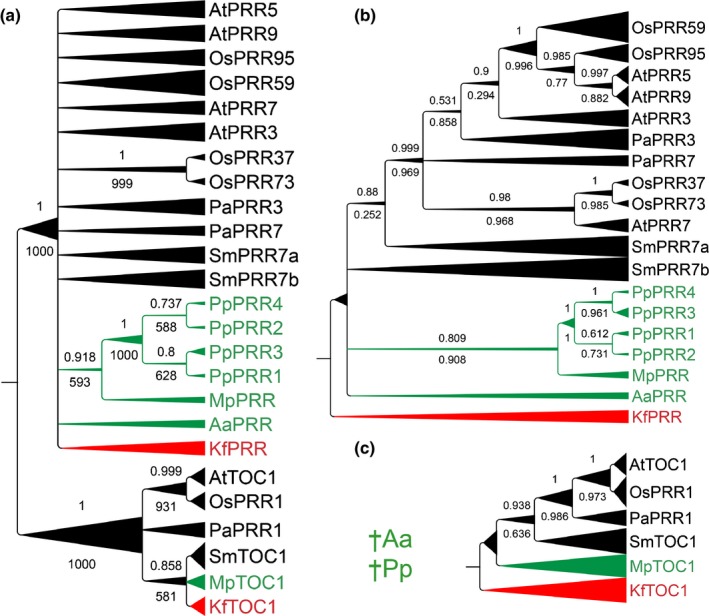
Inferred phylogeny of homologues to the PSEUDO‐RESPONSE REGULATOR (PRR) gene family. Separate trees were constructed from the complete alignment (a), the PRR subfamily (b), and the TOC1 subfamily (c). Trees were constructed using MrBayes and PhyML on an amino acid alignment of proteins retrieved from *Arabidopsis thaliana* (At), *Oryza sativa* (Os), *Picea abies* (Pa), *Selaginella moellendorffii* (Sm), *Physcomitrella patens* (Pp), *Marchantia polymorpha* (Mp), *Anthoceros agrestis* (Aa), and *Klebsormidium flaccidum* (Kf). The Bayesian tree is shown with posterior probabilities (above) and bootstrap proportions from PhyML analysis (below) for each node. Nodes with conflicting support from the two methods were collapsed. Branch length is relative to the thickness of individual branches: the shortest branches have a straight line and the longest are increasingly triangular. †Aa and †Pp indicate loss of TOC1 in Aa and Pp, respectively (as well as all surveyed hornworts and mosses).

One and two genes from this family are found in *O. tauri* and *C. reinhardtii*, respectively (Table 1; Matsuo *et al*., 2008; Corellou *et al*., 2009), but it is unclear whether these are more closely related to *TOC1* or to the other *PRR*s. *K. flaccidum* contains two *PRR* family genes, one in a clade with At*TOC1* and the other one in a clade with At*PRR3*/*7* (Fig. 2; Table 1). In *A. agrestis*, only one *PRR* gene was found, belonging to the *PRR3/7* clade (Aa*PRR*; Fig. 2), while in *P. patens*, four *PRR* genes, all belonging to the PRR3/7 clade, were identified (Fig. 2; Table 1). In oneKP database searches, *TOC1*‐related genes were not found in moss or hornwort transcriptomes (41 and eight species, respectively).

In *M. polymorpha* two *PRR* genes were identified of which one clustered in a clade with AtTOC1 (Mp*TOC1*), and one with the AtPRR3/7 clade (Mp*PRR*; Fig. 2; Table 1). We conclude that a first duplication event resulting in separate *TOC1* and *PRR3/7* paralogues occurred before or during the evolution of charophytes. Furthermore, TOC1 paralogues were lost in both hornworts and mosses.

#### EARLY FLOWERING 3

ELF3 is predicted to encode a soluble protein that is particularly rich in serine, proline, and glutamine (Hicks *et al*., 2001). No ELF3 homologues have been detected in green algae, but a single homologue was found in *K. flaccidum*,* M. polymorpha*,* A. agrestis*, and *Selaginella moellendorffii*, and three homologues were detected in *P. patens* (Fig. S2; Table 1). As previously reported, no homologue was detected in *Picea abies*, but two homologues were found in Arabidopsis and *Oryza sativa* (Fig. S2; Table 1). Thus, data suggest that ELF3 arose in charophytes.

#### EARLY FLOWERING 4

ELF4 is a small protein with a conserved domain of unknown function (DUF1313; Li *et al*., 2016). A putative protein with a DUF1313 domain has been reported in *C*. *reinhardtii*, but no ELF4 homologue was found in the early diverging green alga *O*. *tauri* (Tables 1, S1). Two homologues were found in *K. flaccidum*,* A. agrestis* and *S. moellendorffii*, while a single ELF4 homologue was identified in each of *M. polymorpha* and *P. patens* (Fig. S3; Table 1). *P. abies*, Arabidopsis and *O. sativa* contain five, five and three homologues, respectively (Fig. S3; Table 1). Previous phylogenetic analyses in angiosperms have identified two clusters of ELF4 or ELF4‐like (from now on referred to as EFL) proteins, one corresponding to the AtELF4/AtEFL1 clade in our analyses, and the other corresponding to the clade including AtEFL2, 3, 4. It should be noted that only proteins in the first clade (AtELF4 and AtEFL1) have been shown to possess circadian clock function (Kolmos *et al*., 2009).

#### LUX ARRHYTHMO

LUX and its homologue BROTHER OF LUX (BOA) are MYB‐like transcription factors, belonging to the SANT superfamily (Dai *et al*., 2011). Proteins with similarity to the LUX MYB‐domain are found in green algae (Tables 1, S1). In *K. flaccidum* two proteins were identified with BLAST. One of them had a high e‐value, but as reciprocal BLAST against Arabidopsis identified AtBOA as the top hit, this protein was included in the phylogeny. Single homologues to AtLUX were identified in *A. agrestis* and *M. polymorpha* (Fig. S4; Table 1). Three LUX homologues were identified in *P. patens*, one in *S. moelendorffii* and *O. sativa*, whereas two were identified in *P. abies* (Fig. S4; Table 1).

#### GIGANTEA

*GIGANTEA* is a large well‐conserved plant‐specific protein with no known domains and unknown biochemical function. Remarkably, a single *GI* copy is found in most land plant species, including Arabidopsis, *S. moelendorffii*,* O. sativa* and *P. abies*. No trace of *GI*‐like sequences were found in green algae or in *K*. *flaccidum*; however, GI homologues were detected in the more recently diverged charophytes *Coleochaete irregularis* (Coleochaetales) and *Cylindrocystis cushleckae* (Zygnematales). Searches in the oneKP database confirmed that charophytes, belonging to Zygnematales and Coleochaetales, do contain single *GI* homologues. A single *GI* gene is found also in *M. polymorpha* and *A. agrestis*, but no gene with detectable homology to *GI* was found in *P. patens* (Holm *et al*., 2010; Fig. S5; Table 1). According to BLAST searches in oneKP databases, the only moss, out of 41 species in 31 genera, that contains a homologue to GI is the early diverging moss *Takakia lepidozioides*. These data indicate that GI first occurred in charophytes suggested as sisters to land plants, but was uniquely lost within the moss lineage.

#### ZEITLUPE

In Arabidopsis there are three ZTL‐family F‐box proteins which are involved in blue‐light regulated protein degradation: ZTL, FKF1 and LOV KELCH PROTEIN2 (LKP2; Schultz *et al*., 2001). The genes of this family have three main domains; a Light, oxygen or voltage domain (LOV; belongs to the Per‐ARNT‐Sim (PAS) superfamily), a central F‐box and multiple Kelch‐repeats in the C‐terminus. The LOV domain confers blue‐light sensing, while F‐box and Kelch‐repeats strongly suggest a role in ubiquitin‐mediated protein degradation (Ito *et al*., 2012).

As for ELF3, homologues to the ZTL family are first observed in charophytes. One homologue was identified in each of *K. flaccidum*,* M. polymorpha*,* A. agrestis*, and *S. moelendorffii* (Fig. S6; Table 1). Previously, it has been shown that the *P. patens* genome does not contain any ZTL homologue (Holm *et al*., 2010). As for GI, a BLAST search against oneKP revealed that the only moss, among the 41 species in the database, that expresses a ZTL/FKF1 homologue is *T. lepidozioides*.

In summary, homologues to most of the core clock genes and important clock‐associated genes identified in Arabidopsis were not only found in bryophytes but also in charophytes, suggesting that the circadian clockwork of land plants may have arisen earlier than previously assumed, perhaps in land‐living charophyte algae (Table 1). Our data also suggest that besides gene duplication resulting in redundancy and functional diversification, gene loss has been important in shaping circadian clocks in hornworts, liverworts and mosses. The three genes that previously have been shown to be missing in *P. patens* (*GI*,* ZTL* and *TOC1*; Holm *et al*., 2010) were not found in any moss except the basal moss species *T. lepidozioides*. *GI* and *ZTL* were identified in liverworts, hornworts and charophytes, but *TOC1* was also absent from hornworts. Furthermore, our phylogenetic analyses suggest that liverworts have lost their *CCA1* orthologue.

### Knockout mutants support a role for Mp*PRR*, Mp*RVE* and Mp*TOC1* in the *M. polymorpha* circadian clock

The identification of orthologues of most angiosperm circadian clock genes in early land plants prompted us to start probing their role as circadian clock genes. As *M. polymorpha* has emerged as a model species with well‐developed molecular genetic tools, we investigated if in particular Mp*PRR*, Mp*TOC1*, and Mp*RVE* are required for circadian rhythmicity. First, using qRT‐PCR we found that a majority of putative *M. polymorpha* clock genes, including Mp*PRR*, Mp*TOC1* and Mp*RVE*, showed diel expression patterns in mature thalli grown in ND conditions (Table 2; Fig. S7). The rhythmic expression of most of these genes also persisted after the transition to LL and DD conditions (Table 2; Fig. S7).

**Table 2:** Rhythms in *Marchantia polymorpha* clock gene transcript abundance identified by quantitative reverse transcription polymerase chain reaction (qRT‐PCR) and JTK_CYCLE analysis

Gene	Condition^a^	*P*‐value^b^	Period
Mp*PRR*	ND	**< 0.001**	24
	DD	**0.001**	28
	LL	**< 0.001**	28
Mp*TOC1*	ND	**< 0.001**	24
	DD	**< 0.001**	28
	LL	**0.001**	28
Mp*RVE*	ND	**0.003**	24
	DD	**0.020**	28
	LL	0.061	28
Mp*ELF3*	ND	**0.005**	24
	*DD*	0.073	28
	LL	**0.008**	28
Mp*GI*	ND	**0.035**	20
	DD	**< 0.001**	28
	LL	0.080	28
Mp*LUX*	*ND*	0.073	20
	DD	**0.007**	28
	LL	0.149	28
Mp*EFL*	ND	1	20
	DD	1	28
	LL	1	28
MpFKF	ND	1	28
	DD	0.121	28
	LL	0.225	24

a Light condition: neutral day (ND; 12 : 12 h, light : dark cycles), constant darkness (DD) or constant light (LL).

b Adjusted *P*‐values < 0.05 are presented in bold.

**Figure 3:**
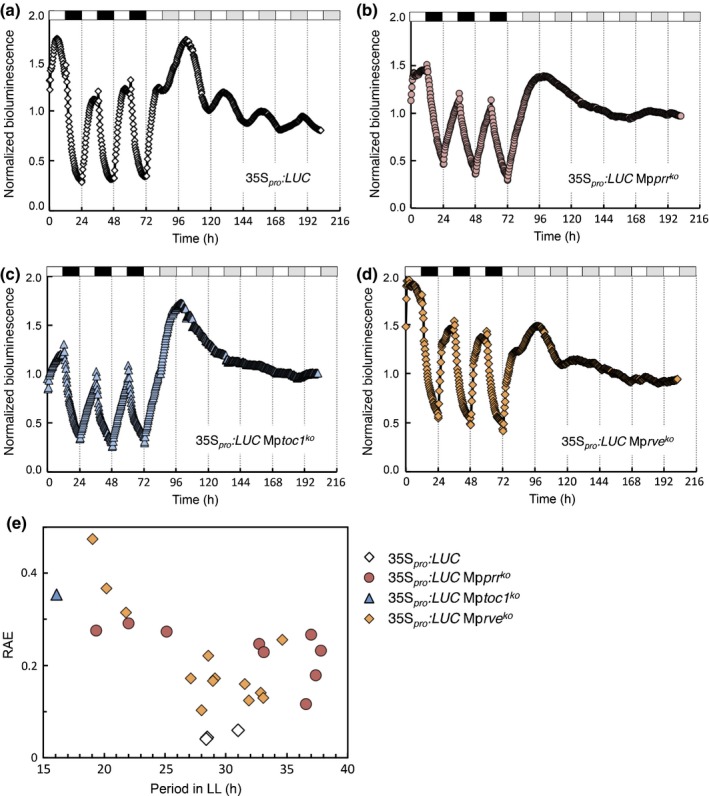
Bioluminescence rhythm of 35S_*pro*_ *:LUC* is abolished in *Marchantia polymorpha* knockout mutants of Mp*PRR*, Mp*RVE* and Mp*TOC1*. Bioluminescence was recorded under neutral‐day (ND; 12 : 12 h, light : dark cycles) and constant‐light (LL) conditions in wild‐type (Tak‐1) and clock mutants. Normalized bioluminescence for representative lines are shown, as follows: (a) 35S_*pro*_ *:LUC*; (b) 35S_*pro*_ *:LUC* Mp*prr* ^*ko*^; (c) 35S_*pro*_ *:LUC* Mp*toc1* ^*ko*^; (d) 35S_*pro*_ *:LUC* Mp*rve* ^*ko*^. (e) Estimates of period plotted against relative amplitude error (RAE). Each plotted symbol in (e) represents an independent line.

Next, we generated knockout lines of Mp*PRR*, Mp*TOC1* and Mp*RVE* in F1 sporelings derived from crosses between Tak‐1 and Tak‐2 (Figs S8–S10), and utilized a luciferase reporter controlled by a CaMV35S promoter (35S_*pro*_ *:LUC*) to assay bioluminescence in transgenic lines. As previously observed in the angiosperm *Lemna gibba* (Muranaka *et al*., 2014), 35S_*pro*_ *:LUC* lines showed diel oscillation under ND and persistent rhythm in LL for several days (Fig. 3). In white light, relative amplitude error (RAE)‐weighted period mean for three independent 35S_*pro*_ *:LUC* lines was 28.9 h, with RAE < 0.07 for each line. Analysis of mRNA levels of *LUC* under equivalent conditions revealed a diel rhythm under ND that markedly dampened upon transfer to LL (Fig. S11). Thus, it is not obvious that the bioluminescence rhythm seen with 35S_*pro*_ *:LUC* in LL is caused primarily by a transcriptional rhythm. Irrespective of the cause of the circadian rhythm in 35S_*pro*_ *:LUC* bioluminescence, a perturbation of this rhythm in knockout lines of putative clock genes would suggest a role for these genes in the generation of circadian rhythmicity. Therefore, we introduced 35S_*pro*_ *:LUC* into knockout lines of Mp*PRR*, Mp*RVE* and Mp*TOC1*. Several reporter lines were obtained for Mp*prr* ^*ko*^ and Mp*rve* ^*ko*^, but only one line for Mp*toc1* ^*ko*^. Bioluminescence rhythm was strongly affected in all knockout lines. Under ND conditions, the 35S_*pro*_ *:LUC* Mp*prr* ^*ko*^ and 35S_*pro*_ *:LUC* Mp*toc1* ^*ko*^ lines showed a pattern with an almost linear increase and decrease in light and dark, respectively, while 35S_*pro*_ *:LUC* Mp*rve* ^*ko*^ showed a pattern more similar to the 35S_*pro*_ *:LUC* lines (Fig. 3). In LL, bioluminescence rhythm was abolished in all knockout lines (Fig. 3). Obtained period estimates consequently varied widely and RAEs were high (Fig. 3). These results show that Mp*PRR*, Mp*RVE* and Mp*TOC1* do have a role in controlling the bioluminescence rhythm observed in 35S_*pro*_ *:LUC*, and suggest that the *M. polymorpha* circadian clock generates this rhythm.

**Figure 4:**
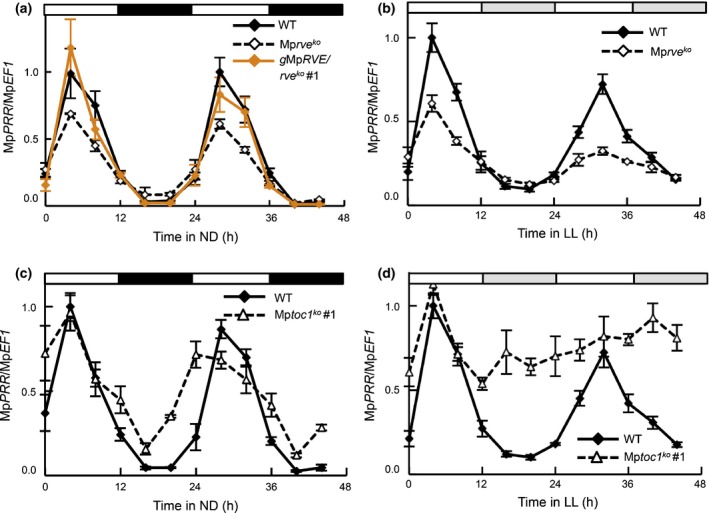
Temporal expression of Mp*PRR* in neutral‐day (ND; 12 : 12 h, light : dark cycles) and constant‐light (LL) conditions is affected in knockout mutants of putative *Marchantia polymorpha* circadian clock genes. (a, b) Expression of Mp*PRR* in wild‐type (WT; Tak‐1, solid line), Mp*rve* ^*ko*^ mutant (broken line) and restored mutant (orange line) under ND conditions (a) and in WT and mutant under LL conditions (b). (c, d) Expression of Mp*PRR* in WT (Tak‐1, solid line) and Mp*toc1* ^*ko*^ mutant (broken line) under ND (c) and LL conditions (d). Mean expression values measured using quantitative reverse transcription polymerase chain reaction are shown with standard errors based on three biological replicates. Expression was normalized using Mp*EF1*.

To further test these conclusions, we studied Mp*PRR* expression in Mp*rve* ^*ko*^ and Mp*toc1* ^*ko*^ knockout lines with qRT‐PCR. If these genes are part of transcriptional feedback loops typical of circadian clocks, we should expect changes in their temporal expression patterns. In Mp*rve* ^*ko*^ lines, reduced peak levels of Mp*PRR* expression were observed in both ND and LL (Fig. 4a,b). The deviant expression patterns in ND were restored by the introduction of a genomic fragment of Mp*RVE* into the mutant (Figs 4a, S12a), suggesting that Mp*RVE* positively regulates Mp*PRR*, similar to the role of At*RVE4, 6, 8* in promoting expression of evening‐phased genes in Arabidopsis (Rawat *et al*., 2011; Hsu *et al*., 2013).

In Mp*toc1* ^*ko*^ lines grown under ND conditions, night‐time decrease in Mp*PRR* expression was slightly reduced (Figs 4c, S12b). In LL, expression of Mp*PRR* remained at peak levels, resulting in arrhythmicity (Figs 4d, S12c). Thus, these data support the idea that Mp*TOC1* directly or indirectly represses the expression of Mp*PRR*, supporting a conserved role for *TOC1* in land plants.

Collectively, these data support the idea that Mp*PRR*, Mp*RVE* and Mp*TOC1* have central roles in the *M. polymorpha* circadian clock and that these roles are at least partially conserved in plants.

### Characterization of circadian rhythms in *M. polymorpha*

**Figure 5:**
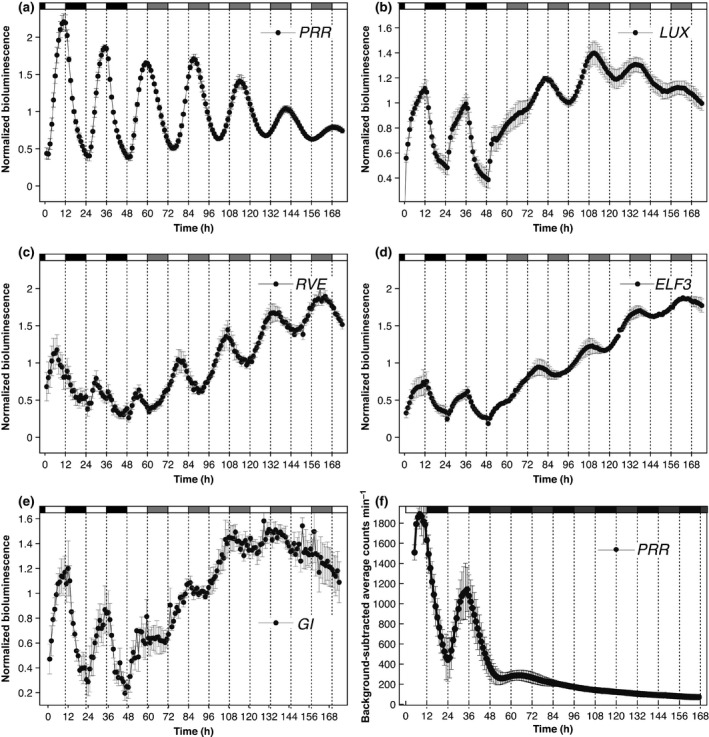
Luciferase reporter lines driven by *Marchantia polymorpha* clock gene promoters show rhythmic expression under neutral‐day (ND; 12 : 12 h, light : dark) and constant‐light (LL) cycles. (a, f) Mp*PRR*_*pro*_ *:LUC*; (b) Mp*LUX*_*pro*_ *:LUC*; (c) Mp*RVE*_*pro*_ *:LUC*; (d) Mp*ELF3*_*pro*_ *:LUC*; (e) Mp*GI*_*pro*_ *:LUC*. Transcriptional reporter lines were entrained under ND conditions and transferred to LL (a–e) or constant darkness (DD; f). Means of triplicates of representative reporter lines are shown with standard errors. Light periods are represented by white areas and dark periods by black areas on top of the graphs. In (a–e), grey areas represent subjective night in LL conditions. In (f), grey areas represent subjective day in DD conditions. (a–e) Normalized luminescence; (f) background‐subtracted average counts min^–1^.

To further study circadian rhythms of *M. polymorpha* clock genes, promoter activities of Mp*ELF3*, Mp*GI*, Mp*LUX*, Mp*PRR* and Mp*RVE* were analysed using transgenic promoter:LUC reporter lines. All lines showed distinct diurnal bioluminescence rhythms in ND (Fig. 5a–e). Significant rhythms were detected for all genes in LL, but rapid dampening of the bioluminescence signal was observed in DD (Fig. 5a–f). This rapid dampening of the luciferase signal in DD seems at odds with evidence for rhythmic expression of several of these genes in DD in qRT‐PCR assays, and suggests that the reduced bioluminescence signal may be a result of an attenuated luciferase activity in DD (e.g. owing to low concentrations of ATP) rather than a reduction in transcription of clock genes. Still, no increase in signal was observed after the addition of sucrose to culture media. With one exception (Mp*PRR*), the amplitudes in ND and LL were also modest. In these conditions, amplitudes in qRT‐PCR assays and bioluminescence assays were generally in agreement, indicating that Mp*PRR* is an exception in exhibiting a strong transcriptional rhythm in both light driven and free‐running conditions.

**Figure 6:**
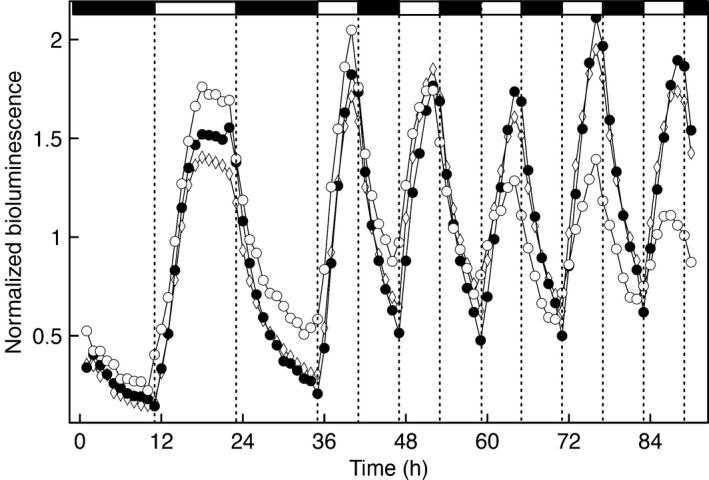
Expression of *Marchantia polymorpha* circadian clock genes is strongly driven by light. Mp*PRR*_*pro*_ *:LUC* bioluminescence of plants entrained in a 12 : 12 h, light : dark photoperiod and transferred to *T* = 12 photocycles. Light intensity was set to 5 μmol m^−2^ s^−1^. Data from three independent transformants are shown. Expression patterns were readily adjusted to a *T* = 12 photocycle without frequency demultiplication.

The apparently weak amplitudes of circadian rhythms and the rapid dampening of rhythms in DD prompted us to investigate further the relative importance of light‐driven vs circadian control on clock gene expression in *M. polymorpha*. In environmental cycles with time periods close to half the circadian period, circadian rhythms tend to frequency demultiply, meaning that every other environmental cycle is skipped (Bruce, 1960; Thines & Harmon, 2010). Luciferase reporter lines driven by *M. polymorpha* clock gene promoters were entrained in ND, and transferred to a T‐cycle of 12 h (6 : 6 h, light : dark). Even for the Mp*PRR*_*pro*_ *:LUC* lines displaying the strongest rhythm in LL, an immediate adoption of a 12 h period was evident and no tendency for frequency demultiplication could be observed (Fig. 6). The same result was obtained with Mp*ELF3*, Mp*GI*, Mp*LUX* and Mp*RVE*, suggesting that the *M. polymorpha* clock is strongly driven by light changes and that the circadian rhythms are generally weak (Fig. S13).

**Figure 7:**
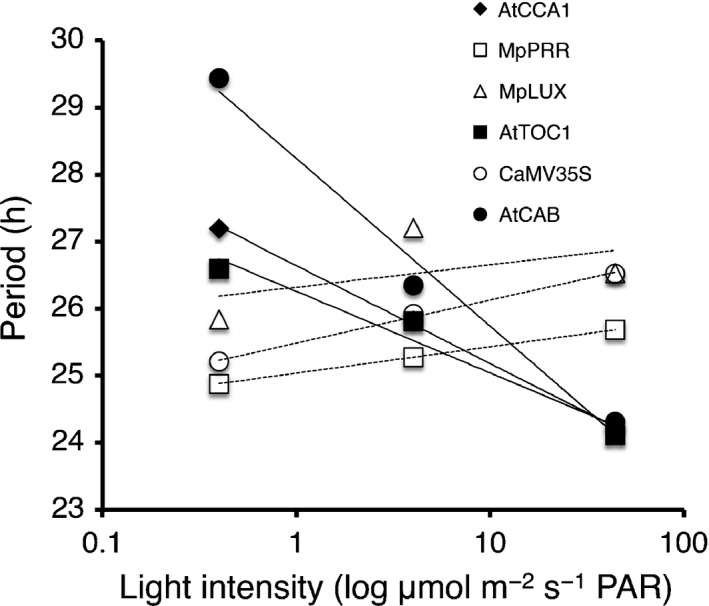
*Marchantia polymorpha* circadian clock genes do not follow Aschoff's rule. Periods of circadian rhythm for Arabidopsis (closed symbols) and *M. polymorpha* (open symbols) promoter:LUC lines were estimated in different intensities of continuous red light. Plants harbouring promoter:LUC constructs as indicated in the figure were first entrained in red and blue light, and then released into 0.4, 4 or 44 μmol m^−2^ s^−1^ of red light. Period in constant light (LL) was estimated with Spectrum Resampling (Costa *et al*., 2013) and plotted against logged light intensity. PAR, photosynthetically active radiation.

**Figure 8:**
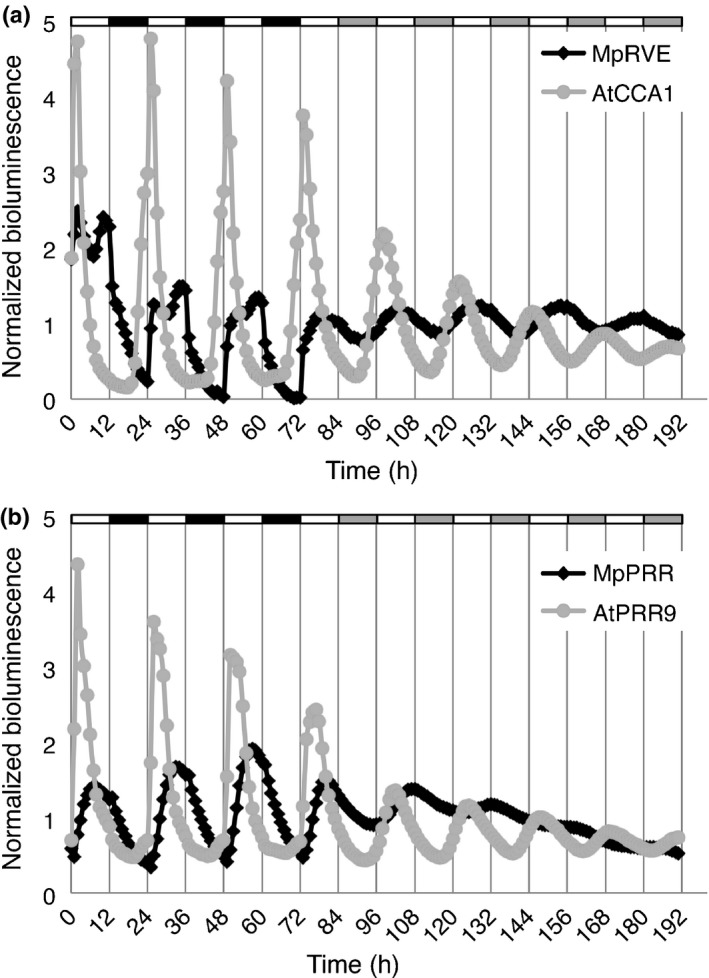
Comparison of bioluminescence rhythm for Arabidopsis and *Marchantia polymorpha* promoter:LUC reporter lines. (a, b) Bioluminescence of Mp*RVE*_*pro*_ *:LUC*, At*CCA*1_*pro*_ *:LUC* (a), and Mp*PRR*_*pro*_ *:LUC* and At*PRR9*_*pro*_ *:LUC* (b). Plants were entrained in neutral‐day conditions (ND; 12 : 12 h, light : dark cycles), monitored for 3 d and released into constant light (LL) and monitored for an additional 5 d.

To further characterize and directly compare circadian rhythms observed in *M. polymorpha* with those of Arabidopsis, bioluminescence rhythms for both species were simultaneously compared under different light qualities and intensities. After entrainment in blue plus red light, plants were released into different intensities of red light. As expected, At*CCA1*_*pro*_ *:LUC*, At*PRR9*_*pro*_ *:LUC*, At*TOC1*_*pro*_ *:LUC* and At*CAB*_*pro*_ *:LUC* displayed a clear shortening of period with increasing light intensity (Fig. 7; Somers *et al*., 1998). By contrast, all *M. polymorpha* lines, including 35S_*pro*_ *:LUC*, showed a slight increase in period with increased light intensity (Fig. 7). These results suggest that the role of light in entrainment of the *M. polymorpha* clock is different from that of Arabidopsis. One striking difference between rhythms observed in Arabidopsis and *M. polymorpha* was the absence of early‐phased genes in the latter. In Arabidopsis, both *CCA1/LHY* and *PRR9* have an early morning phase with peaks at and shortly after dawn, respectively. A homologue to *CCA1/LHY* is lacking in *M. polymorpha* and the closest relative, Mp*RVE*, showed a broad expression peak throughout the light period (Fig. 8a). Additionally, the closest homologue to *PRR9*, Mp*PRR*, showed an afternoon peak of Mp*PRR*_*pro*_ *:LUC* activity, although Mp*PRR* mRNA levels peaks earlier during the day (Figs 4, 5a, 8b, S11a, S12). Both *CCA1/LHY* and *PRR9* are also acutely induced by light, and CCA1/LHY have been suggested to be important for entrainment at dawn (Wang & Tobin, 1998); however, no such acute induction was evident for our assayed *M. polymorpha* genes (Figs 5, 8). This apparent lack of dawn‐phased genes with acute light response might explain the lack of period shortening with increased light intensity observed for *M. polymorpha*.

### *Marchantia polymorpha* clock genes are highly expressed in meristematic regions

**Figure 9:**
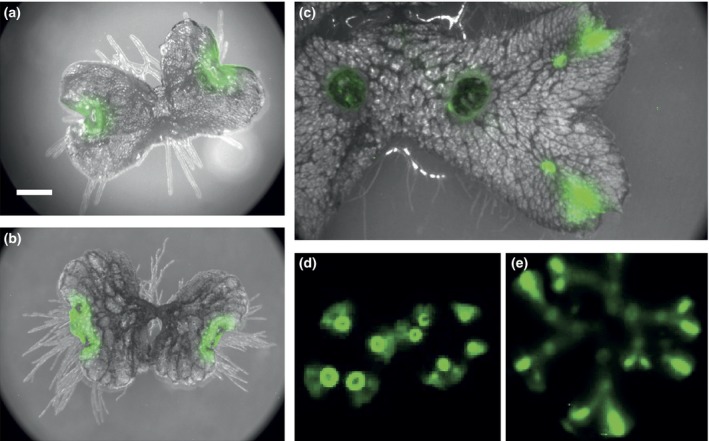
The Mp*PRR* promoter drives luciferase expression in meristematic regions. Luciferase imaging in transgenic *Marchantia polymorpha* plants expressing luciferase under the control of the Mp*PRR* promoter. (a–e) Mp*PRR*_*pro*_ *:LUC*: (a) 4‐d‐old gemmaling showing expression at the apical notches; (b) 7‐d‐old gemmaling with expression at the recently split apical notches; (c) 4‐wk‐old thallus showing strong expression in apical regions and young gemma cups; (d) 3‐wk‐old thallus grown on 2% sucrose with high expression in young gemma cups; (e) 5‐wk‐old thallus showing weak expression along the midrib. Bioluminescense is pseudocolored in green. Illuminated images are shown in grey in (a–c), but omitted from (d) and (e) to reveal weak signals.

To investigate the spatial expression pattern of *M. polymorpha* clock gene homologues, we assayed bioluminescence on different developmental stages of *M. polymorpha* using luciferase reporter lines for Mp*ELF3*, Mp*GI*, Mp*LUX*, Mp*PRR* and Mp*RVE*. We also generated transgenic plants harboring Mp*PRR*_*pro*_ *:GUS* to better assay patterns of weak expression. For all *LUC*‐lines, bioluminescence was detected in the apical thallus in and/or around the apical cell (Figs 9, S14). In gemmalings, expression was confined to the apical notches (Figs 9a,b, S14). Expression was also seen in the developing gemma cups, but as the cups matured, this expression markedly decreased and was confined to the walls of the cup (Figs 9c–e, S14). After extended exposure, a weak signal could sometimes be detected along the midrib in older thalli (Figs 9e, S14). Mp*PRR*_*pro*_ *:GUS* lines support a strong expression in meristematic regions, but prolonged staining also revealed expression in chlorenchyma cells within air chambers (Fig. S15). These observations suggest that the *M. polymorpha* circadian clock is highly active in meristematic or rapidly dividing cells and tissues, but also in photosynthetically active tissues.

### Temporal expression patterns support a role also for hornwort clock gene homologues in a circadian clock

Quantitative RT‐PCR was also used to study endogenous transcript abundance of putative *A. agrestis* clock genes identified earlier. These data suggested that a majority of the assayed genes showed a clear diurnal rhythm under ND. Significant *P*‐values from analysis of rhythmicity with JTK‐cycle were obtained for all genes except Aa*RVE* (Table 3; Fig. S16). Most genes also displayed a significant free‐running circadian rhythm under at least one constant condition (LL or DD). The exception was Aa*RVE*, which lacked evidence of significant rhythmic expression under all conditions (Table 3; Fig. S16). Data suggest an early morning phased expression of Aa*CCA1*, and a near dusk expression of Aa*ELF3*, Aa*EFL‐1*,* 2*, Aa*GI*, Aa*LUX* and Aa*PRR*. Low amplitude of Aa*RVE* expression precluded estimation of phase for this gene. qRT‐PCR data also support rhythmic expression under day–night cycles or free‐running conditions for additional putative circadian clock genes in *M. polymorpha* (Table 2; Fig. S7).

**Table 3:** Circadian rhythms in *Anthoceros agrestis* clock gene transcript abundance identified by JTK_CYCLE

Gene	Condition^a^	*P*‐value^b^	Period
Aa*CCA1*	ND	**< 0.001**	24
	LL	**0.001**	24
	DD	1	28
Aa*RVE*	ND	0.225	24
	LL	1	20
	DD	1	28
Aa*PRR*	ND	**< 0.001**	24
	LL	0.598	20
	DD	0.261	24
Aa*ELF3*	ND	**0.002**	20
	LL	0.225	20
	DD	**0.029**	24
Aa*GI*	ND	**0.004**	24
	LL	1	28
	DD	0.225	20
Aa*LUX*	ND	**< 0.001**	24
	LL	**0.035**	28
	DD	**0.010**	24
Aa*ZTL*	ND	**< 0.001**	20
	LL	0.121	20
	DD	1	24
Aa*EFL‐1*	ND	**< 0.001**	24
	LL	**0.024**	28
	DD	**0.020**	24
Aa*EFL‐2*	ND	<** 0.001**	24
	LL	**0.013**	28
	DD	0.073	24

a Light condition: neutral day (ND; 12 : 12 h, light : dark cycles), constant darkness (DD) or constant light (LL).

b Adjusted *P*‐values < 0.05 are presented in bold.

In conclusion, temporal expression patterns are consistent with a role in the circadian clock for a majority of homologues to Arabidopsis clock genes identified in the hornwort *A. agrestis* and the liverwort *M. polymorpha*. Estimates of phase are also in agreement with an *A. agrestis* clock model consisting of a dawn‐phased CCA1/LHY homologue and evening‐phased genes including those belonging to the EC (*ELF3*,* ELF4* and *LUX* homologues). While Arabidopsis contains several PRR‐family genes with phases ranging from morning to evening, one and two homologues were detected in *A. agrestis* and *M. polymorpha*, none of which showed a dawn‐phased expression. The apparent lack of dawn‐phased components in *M. polymorpha* might explain some of the differences in clock behaviour observed between *M. polymorpha* and Arabidopsis.

## Discussion

Transition through a shallow fresh water environment to a terrestrial environment probably had a profound impact on the plant circadian clock and the processes it controls. In marine algae, the circadian clock is important for control of, for example, phototaxis, chemotaxis and cell division (Bruce, 1970; Byrne *et al*., 1992; Goto & Johnson, 1995; Moulager *et al*., 2007). In angiosperms the circadian clock regulates a wide range of processes, including those affecting metabolism, growth, abiotic and biotic stress, and various photoperiodic responses (Greenham & McClung, 2015, and references therein).

### A majority of plant circadian clock genes are present in charophytes

Evolution of the plant circadian clock is characterized by an increase in gene number and interactions through additional feedback loops. This has partly been accomplished through gene duplication and functional divergence but also through the acquisition of new genes. While green algae clocks can be modelled as a simple two‐gene, one‐loop circuit, angiosperm clocks seem to consist of a complex multi‐loop network comprising many genes.

Our data suggest that all the known major core clock components present in angiosperms were actually present already in charophytes. Novel components in charophytes, which are crucial in angiosperm clocks, are the EC genes *ELF3*,* ELF4* and *LUX*, but also *GI* and genes of the *ZTL* family. Charophytes also contain both TOC1 and PRR3/7 homologues. The function of the EC genes in charophytes or bryophytes is, to our knowledge, unknown. Besides an important function in control of the circadian rhythm, the EC in Arabidopsis is also implicated in output processes such as circadian and temperature control of hypocotyl growth (Nusinow *et al*., 2011; Box *et al*., 2015).

In angiosperms, the GI protein interacts with several proteins, including ZTL family proteins, ELF3 and ELF4, to regulate protein stability and localization (references in Kim *et al*., 2013). In *M. polymorpha*, Kubota *et al*. (2014) showed that MpGI interacts with the single ZTL family member MpFKF and affects photoperiodic induction of reproduction, similar to the role of AtFKF1 in flower induction. A role for nonangiosperm GI and ZTL family proteins in clock function has, to our knowledge, not been reported. The GI protein is a large protein without known protein domains. Its protein sequence is also highly conserved, and remarkably the gene occurs in most species as a single copy. No homologous proteins are detected outside Streptophyta, and within this group homologous proteins can be found in Embryophytes, Zygnematales and Coleochaetales, but not in, for example, Klebsormidiales. This pattern supports the notion that Zygnematales or Coleochaetales is sister to land plants, and that GI arose *de novo* in parallel with the transition to a terrestrial environment.

### Gene loss has been important in shaping early land plant circadian clocks

Although gene numbers and complexity have generally increased during plant circadian clock evolution, several examples of gene loss are also evident. It was previously suggested that the reduced circadian clock of *P. patens* without *GI*,* TOC1* and *ZTL* homologues might represent an ancestral, less complex, state (Holm *et al*., 2010). It is now clear that the lack of these three components is the result of gene loss. Interestingly, homologues to *GI*,* TOC1* and *ZTL* are present in the basal moss *T. lepidozioides*. This was originally described as a liverwort, but retrieval and analysis of reproductive structures resulted in reclassification of the genus *Takakia* as a basal moss (Smith, 1993). Following this reclassification, this set of clock genes was lost very early after the branching of mosses. In Arabidopsis these three genes show close interaction, as physical interaction between GI and ZTL proteins regulate TOC1 stability (Más *et al*., 2003; Kim *et al*., 2009). It is thus intriguing that these three genes were all lost early in the moss lineage. In hornworts, on the other hand, our data suggest that homologues to GI and ZTL are retained and only TOC1 was lost. An additional intriguing gene loss is *CCA1* in liverworts. CCA1 has until now been considered a fundamental component of all plant circadian clocks, from algae to angiosperms. Functional studies of circadian clock genes in bryophytes will shed light on the effects of these gene losses and give a better understanding of the evolution of the plant circadian clock.

### Are circadian clocks of early land plants less robust and do they control fewer processes?

Analysis of circadian rhythms of putative clock genes in *M. polymorpha* suggests that these rhythms are weak compared with those in angiosperms. With one exception (Mp*PRR*), significant but weak rhythms were detected for *M. polymorpha* genes in constant conditions. In *P. patens*, clear rhythms are seen in DD but not in LL conditions, while in the gymnosperm *P. abies* a rapid dampening of rhythms was observed for clock gene homologues in both LL and DD conditions (Holm *et al*., 2010; Gyllenstrand *et al*., 2014). Even though a free‐running clock in constant conditions is often considered a hallmark of circadian rhythm, the advantage of such a property is not entirely obvious, as plants occur in a geophysical rhythmic environment. One advantage of a complex free‐running oscillator could be increased resilience to external noise (Brown *et al*., 2012). It has been suggested that a less complex clock with fewer interlocked feedback loops and less control of degradation of its component results in a more rapidly dampened timer (Brown *et al*., 2012). If circadian clocks of nonangiosperm plants are less complex or less resilience to external noise awaits further study.

Analysis of LUC and GUS reporter lines driven by promoters of putative *M. polymorpha* clock genes suggested that these genes were predominantly expressed in meristematic regions, but weaker expression was also detected in chlorenchyma cells within air chambers. This suggests that basic metabolic processes such as photosynthesis and starch metabolism may also be under circadian control in *M. polymorpha*. Processes confined to meristematic regions that are probably under circadian control include photoperiodic induction of reproduction. Gametangia production in *M. polymorpha* is induced by long photoperiods and requires Mp*GI* and Mp*FKF* (Voth & Hamner, 1940; Kubota *et al*., 2014).

Our inventory of circadian clock gene homologues in charophytes and bryophytes suggests an early acquisition of a complex circadian network, with all known main components already present before or concurrent with the occurrence of land plants, although in lower copy numbers. It has been suggested that physiological adaptations to land had already evolved in early terrestrial charophytes (Delwiche & Cooper, 2015; Harholt *et al*., 2016). Work on cell wall evolution (Mikkelsen *et al*., 2014) suggests that key adaptations to terrestrial habitats had already occurred in *Klebsormidium*, which is in line with our observations of an early complexity of the circadian network. Our data provide good grounds for pursuing functional studies of circadian clocks not only in bryophytes but also in charophytes. Our preliminary observations of circadian rhythms in *M. polymorpha*, suggesting weak rhythms, lack of morning‐phased genes that show acute light response, and deviations from predictions of Aschoff's rule for diurnal organisms, point to significant differences in the wiring of the *M. polymorpha* circadian network.

## Author contributions

A‐M.L., A.K., K.H., N.C., N.G., R.N., T.K. and U.L. conceived the project; A‐M.L. and U.L. performed the sequence retrieval and phylogenetic analyses; A‐M.L., A.K., D.M.E., E.R.A.P., K.H., N.G. and U.L. performed the qRT‐PCR experiments; A‐M.L. and A.K. analysed qRT‐PCR data. A.K., D.M.E., E.R.A.P., T.M., T.O. and U.L. performed the luciferase experiments; A‐M.L., A.K., T.M., T.O. and U.L. analysed the luciferase data. N.C. provided new materials. A‐M.L., D.M.E. and U.L. wrote the article with contributions of all the authors.

## Supporting information

Please note: Wiley Blackwell are not responsible for the content or functionality of any Supporting Information supplied by the authors. Any queries (other than missing material) should be directed to the *New Phytologist* Central Office.

**Fig. S1** Alignments used for phylogenetic construction.
**Fig. S2** Inferred phylogeny of homologues to the ELF3 family.
**Fig. S3** Inferred phylogeny of homologues to the ELF4 family.
**Fig. S4** Inferred phylogeny of homologues to the LUX family.
**Fig. S5** Inferred phylogeny of homologues to the GI gene family.
**Fig. S6** Inferred phylogeny of homologues to the ZTL gene family.
**Fig. S7** Temporal expression patterns of putative circadian clock genes in *Marchantia polymorpha* (Mp) under ND, LL and DD conditions.
**Fig. S8** Generation of Mp*RVE* knockout mutant.
**Fig. S9** Generation of Mp*PRR* knockout mutant.
**Fig. S10** Generation of Mp*TOC1* knockout mutants.
**Fig. S11** Temporal expression pattern of Mp*PRR* and 35S_*pro*_ *:LUC* under ND and LL conditions.
**Fig. S12** Temporal expression pattern of Mp*PRR* in WT, Mp*rve* ^*ko*^, Mp*toc1* ^*ko*^ and restored lines of Mp*rve* ^*ko*^.
**Fig. S13** *pro:LUC* bioluminescence for Mp*ELF3*, Mp*GI*, Mp*LUX* and Mp*RVE*.
**Fig. S14** Luciferase imaging in transgenic *Marchantia polymorpha* plants expressing luciferase under the control of *Marchantia polymorpha* promoters.
**Fig. S15** Mp*PRR*_*pro*_ *:GUS* expression in mature thallus.
**Fig. S16** Temporal expression patterns of putative circadian clock genes in *Anthoceros agrestis* (Aa) under ND, LL and DD conditions.
**Table S1** Gene names, family/subclade, gene ID or accession number
**Table S2** Oligonucleotides used in this study
**Methods S1** Supplemental materials and methods describing sequence retrieval, sequence analysis and phylogenetic reconstruction.
