# Supplementary material for: Early evolution of the land plant circadian clock
Source: New Phytol. 2017 Feb 28;216(2):576–90. doi: 10.1111/nph.14487 (PMC5638080; doi:10.1111/nph.14487)
Supplement: Supplementary file 1 — Fig. S1 Alignments used for phylogenetic construction. Fig. S2 Inferred phylogeny of homologues to the ELF3 family. Fig. S3 Inferred phylogeny of homologues to the ELF4 family. Fig. S4 Inferred phylogeny of homologues to the LUX family. Fig. S5 Inferred phylogeny of homologues to the GI gene family. Fig. S6 Inferred phylogeny of homologues to the ZTL gene family. Fig. S7 Temporal expression patterns of putative circadian clock genes in Marchantia polymorpha (Mp) under ND, LL and DD conditions. Fig. S8 Generation of MpRVE knockout mutant. Fig. S9 Generation of MpPRR knockout mutant. Fig. S10 Generation of MpTOC1 knockout mutants. Fig. S11 Temporal expression pattern of MpPRR and 35Spro :LUC under ND and LL conditions. Fig. S12 Temporal expression pattern of MpPRR in WT, Mprve ko, Mptoc1 ko and restored lines of Mprve ko. Fig. S13 pro:LUC bioluminescence for MpELF3, MpGI, MpLUX and MpRVE. Fig. S14 Luciferase imaging in transgenic Marchantia polymorpha plants expressing luciferase under the control of Marchantia polymorpha promoters. Fig. S15 MpPRR pro :GUS expression in mature thallus. Fig. S16 Temporal expression patterns of putative circadian clock genes in Anthoceros agrestis (Aa) under ND, LL and DD conditions. Table S1 Gene names, family/subclade, gene ID or accession number Table S2 Oligonucleotides used in this study Methods S1 Supplemental materials and methods describing sequence retrieval, sequence analysis and phylogenetic reconstruction. [file NPH-216-576-s001.pdf]

New Phytologist Supporting Information

Article title: **Early evolution of the land plant circadian clock**

Authors: Anna-Malin Linde, D. Magnus Eklund, Akane Kubota, Eric R. A. Pederson, Karl Holm, Niclas Gyllenstrand, Ryuichi Nishihama, Nils Cronberg, Tomoaki Muranaka, Tokitaka Oyama, Takayuki Kohchi and Ulf Lagercrantz

Article acceptance date: 18 January 2017

The following Supporting Information is available for this article:

Fig. S1 Alignments used for phylogenetic construction

Fig. S2 Inferred phylogeny of homologs to the ELF3 family

Fig. S3 Inferred phylogeny of homologs to the ELF4 family

Fig. S4 Inferred phylogeny of homologs to the LUX family

Fig. S5 Inferred phylogeny of homologs to the GI gene family

Fig. S6 Inferred phylogeny of homologs to the ZTL gene family

Fig. S7 Temporal expression patterns of putative circadian clock genes in *M. polymorpha* (Mp) under ND, LL and DD conditions

Fig. S8 Generation of Mp*RVE* knockout mutant

Fig. S9 Generation of Mp*PRR* knockout mutant

Fig. S10 Generation of Mp*TOC1* knockout mutants

Fig. S11 Temporal expression pattern of Mp*PRR* and $35S_{pro}$:*LUC* under ND and LL conditions

Fig. S12 Temporal expression pattern of Mp*PRR* in WT, Mp*rve*^{ko}, Mp*toc1*^{ko} and restored lines of Mp*rve*^{ko}

Fig. S13 pro:LUC bioluminescence for Mp*ELF3*, Mp*GI*, Mp*LUX* and Mp*RVE*

Fig. S14 Luciferase imaging in transgenic *M. polymorpha* plants expressing luciferase under the control of *M. polymorpha* promoters

Fig. S15 MpPRR_{pro}*:GUS* expression in mature thallus

Fig. S16 Temporal expression patterns of putative circadian clock genes in *A. agrestis* (Aa) under ND, LL and DD conditions

Table S1 Gene names, family/sub-clade, gene ID or accession number

Table S2 Oligonucleotides used in this study

Methods S1 Supplemental materials and methods describing sequence retrieval, sequence analysis and phylogenetic reconstruction

Fig. S1 Alignments used for phylogenetic construction. Amino acid sequences were aligned using the M-Coffee algorithm in T-Coffee (Notredame *et al.*, 2000; Wallace *et al.*, 2006). Alignments were filtered using Transitive Consistency Score (TCS) in the T-Coffee distribution (Chang *et al.*, 2014). (a) CCA1/LHY/RVE, (b) PRR/TOC1, (c) ELF3, (d) ELF4, (e) LUX, (f) GI, (g) ZTL.

(a)

```
#NEXUS
BEGIN DATA;
dimensions ntax=31 nchar=215;
format missing=?
symbols="ABCDEFGHIKLMNOPQRSTUVWXYZ"
interleave datatype=PROTEIN gap=- match=.;

matrix
OsRVE6       MDRSSSEMPGKKARKPYTITKPRERWSEEEHERFLDALIMYGRDWKKIEE
PaCCA1-3     MLPFSIDEPFSKVRKPYTITKQRERWTEDEHKKFLDALKLYGRSWRHIEE
PaCCA1       MMSSSGDELAAKVRKPYTITKQRERWSEEEHLKFLEALKMYGRAWRRIEE
KfRVE        MLGGKGDDMHGKVRKPYTITKQRENWTEQEHNKFLKALELFDRDWRKIEE
MpRVE        MA-QGSEEGSKKVRKPYTITKSRESWTEQEHDKFLEALQLFDRDWKKIEA
AtRVE5       MVSSFSEDPTTKIRKPYTIKKSRENWTDQEHDKFLEALHLFDRDWKKIEA
AtRVE3       MVTSFFEDPTKKVRKPYTITKSRENWTEQEHDKFLEALHLFDRDWKKIKA
AtRVE4       MTSEAGEAPEKKVRKAYTITKSRESWTEGEHDKFLEALQLFDRDWKKIED
AtRVE8       MSSAVAEGSSKKVRKPYTITKSRESWTEEEHDKFLEALQLFDRDWKKIED
OsRVE5       MSSEVEDDGGRRVRKPYTITKSRESWTDPEHDKFLEALQLFDRDWKKIEA
AaRVE        MF-GVTEEGSKKIRKPYTITKSRESWTEQEHDKFLEALQLFDRDWKKIEA
SmRVE        ---PSDDAVSKKIRKPYTITKSRESWTEQEHDKFLEALQLFDRDWKKIEA
OsRVE4       MVSSAGEDASKKVRKPYTITKSRESWTEQEHDKFLEALQLFDRDWKKIEA
AtRVE6       MVSSSEEDLSKKIRKPYTITKSRESWTEPEHDKFLEALQLFDRDWKKIEA
PpRVE1       MNASVSEEGSKKIRKPYTITKSRESWTEQEHDKFLDALQLFDRDWKKIEA
PpRVE2       MNAPVSEEGSKKIRKPYTITKSRESWTEQEHDKFLEALQLFDRDWKKIEA
PpRVE3       MNASVSEEGSKKIRKPYTITKSRESWTEQEHDKFLEALQLFDRDWKKIEA
KfCCA1       MDMSTSESLSTKVRKPYTITKQRERWTEAEHDRFLEALKLHGRAWRKIEE
PaCCA1-2     MASSSGDEFTTKVRKPYTITKQRERWTEEEHHKFLEALKMHGRAWRRIEE
OsCCA1       ---SSGEEAVVKVRKPYTITKQRERWTEAEHNRFLEALKLYGRAWQRIEE
AtLHY        ---TSGEELLAKARKPYTITKQRERWTEDEHERFLEALRLYGRAWQRIEE
AaCCA1       MNSSSGDELAMKVRKPYTITKQRERWTEEEHTKFLEALKLYGRAWRRIEE
AtCCA1       ---SSGEDLVIKTRKPYTITKQRERWTEEEHNRFIEALRLYGRAWQKIEE
OsRVE2       MARMMDEARIPKARKPYTITKQREKWTEDEHKLFLEALQLHGRAWRRIQE
OsRVE1       MACLSGEEHVPKARKPYTITKQREKWTDEEHRLFLEALQLHGRAWRRIQE
AtRVE7       MLCSHWIENVVKVRKPYTVTKQREKWSEEEHDRFLEAIKLYGRGWRQIQE
AtRVE7Like   MVMSHWIENVVKVRKPYTVTKQREKWSEEEHDRFLEAIKLYGRGWRQIQE
PpCCA1a      ------------VRKPYTITKQRERWTEEEHQRFLEALKLYGRAWRRIEE
PpCCA1b      ---SGDEGASTKVRKPYTITKQRERWTEEEHQKFLEALKLYGRAWRRIEE
OsRVE3       MASEGMEEFPVKVRKPYTITKQREKWTEEEHDKFLEALKLYGRSWRQIQE
```

```
AtRVE2       M--SSSDAFYLKTRKPYTITKQREKWTEAEHEKFVEALKLYGRAWRRIEE
AtRVE1       MASFGGNDYAPKVRKPYTITKERERWTDEEHKKFVEALKLYGRAWRRIEE

OsRVE6       HVGTKTTIQIRSHAQKYFLKVQKMGLAA--GLPPQYPRRRLVMQQQQQQS
OsCCA1-3     HIGTKSAVQIRSHAQKFFTKLEKGASTGYLEIPPPRPKRKPGHPYPKKTG
PaCCA1       HIGTKTAVQIRSHAQKFFSKLVRGSSSKDIDIPPPRPKRKPRHPYPRKAV
KfRVE        YVGTKTVIQIRSHAQKYFLKVQKLGQDA--HVPPPRPKRKSSQPYPQKAS
MpRVE        FVGSKSVIQIRSHAQKYFLKVQKNGTGE--HVPPPRPKRKSAQPYPQKAP
AtRVE5       FVGSKTVVQIRSHAQKYFLKVQKSGANE--HLPPPRPKRKASHPYPIKAP
AtRVE3       FVGSKTVIQIRSHAQKYFLKVQKNGTKE--HLPPPRPKRKANHPYPQKAP
AtRVE4       FVGSKTVIQIRSHAQKYFLKVQKNGTLA--HVPPPRPKRKAAHPYPQKAS
AtRVE8       FVGSKTVIQIRSHAQKYFLKVQKNGTLA--HVPPPRPKRKAAHPYPQKAS
OsRVE5       YVGSKTVIQIRSHAQKYFLKVQKNGTGE--HLPPPRPKRKAAHPYPHKAS
AaRVE        FVGSKTVIQIRSHAQKYFLKVQKNGTGE--HVPPPRPKRKSAQPYPQKAP
SmRVE        FVGSKTVIQIRSHAQKYFLKVQKNGTGE--HVPPPRPKRKSAQPYPQKAA
OsRVE4       FVGSKTVIQIRSHAQKYFLKVQKNGTSE--HVPPPRPKRKAAHPYPQKAS
AtRVE6       FIGSKTVIQIRSHAQKYFLKVQKSGTGE--HLPPPRPKRKAAHPYPQKAH
PpRVE1       FVGSKTVIQIRSHAQKYFLKVQKNRTGE--HVPPPRPKRKSAQPYPQKAS
PpRVE2       FVGSKTVIQIRSHAQKYFLKVQKNGTGE--HVPPPRPKRKSVQPYPQKAP
PpRVE3       FVGSKTVIQIRSHAQKYFLKVQKNGTGE--HVPPPRPKRKSAQPYPQKAP
KfCCA1       HIGSKTAVQIRSHAQKFFSKLEREGSTAEIDIPPPRPKRKPSHPYPRKAG
OsCCA1-2     HIGTKTAVQIRSHAQKFFSKLEREASAGDISIPPPRPKRKPSHPYPRKAG
OsCCA1       HVGTKTAVQIRSHAQKFFTKLEKEAINNDIDIPPPRPKRKPNSPYPRKSC
AtLHY        HIGTKTAVQIRSHAQKFFTKLEKEAEVKDIEIPPPRPKRKPNTPYPRKPG
AaCCA1       HIGTKTAVQIRSHAQKFFSKLEREATAADIDIPPPRPKRKPSHPYPRKAG
AtCCA1       HVATKTAVQIRSHAQKFFSKVEKEAEAKDIAIPPPRPKRKPNNPYPRKTG
OsRVE2       HIGTKTAVQIRSHAQKFFSKVIKESSGDSIQIPPPRPKRKPVHPYPRNLG
OsRVE1       HIGTKTAVQIRSHAQKFFSKVVRESSGSAIQIPPPRPKRKPAHPYPRKVD
AtRVE7       HIGTKTAVQIRSHAQKFFSKMAQEADSRAIVIPPPRPKRKPAHPYPRKSP
AtRVE7Like   HIGTKTAVQIRSHAQKFFSKMAQEADSRAIVIPPPRPKRKPAHPYPRKSP
PpCCA1a      HIGTKTAVQIRSHAQKFFSKIERDVSAGVIDIPPPRPKRKPSHPYPRKAG
PpCCA1b      HIGTKTAVQIRSHAQKFFSKIERDVTAGVIDIPPPRPKRKPTHPYPRKAG
OsRVE3       HIGTKTAVQIRSHAQKFFSKVVREPGS-AIEIPPPRPKRKPLHPYPRKCA
AtRVE2       HVGTKTAVQIRSHAQKFFTKVARDFGV-SIEIPPPRPKRKPMHPYPRKLV
AtRVE1       HVGSKTAVQIRSHAQKFFSKVAREATGGPIVIPPPRPKRKPAHPYPRKFG

OsRVE6       SPA-----MQNLEWASTSGTAAWGNHHGLPFVSFE--------TT-----
OsCCA1-3     VID-----KSTIHKSSVFSNTNWSPA--LSYTHQ-QQEDEKQRNK----L
PaCCA1       GTA-----AEAVAVATVAAASAWWSLYGASLHPR-AENISSV-KAREGIQ
KfRVE        KVV-----TPSKANPDFAK--V-YRFLGDVFEIGVAGNLQKLKCMPPIDR
MpRVE        KAV-----TDLRATPDFAE--V-YKFIGNVFDPGSNHNLKKLKEMAPIDR
AtRVE5       KNV-----KPHRVMPNFAE--V-YSFIGSVFDPNTSGHLQRLKQMDPINM
AtRVE3       KFT-----KPHRVAPNFAE--V-YNFIGSVFDPKTTGHVKRLKEMDPINL
AtRVE4       KNA-----PSMHGLPDFAE--V-YNFIGSVFDPDSKGRMKKLKEMDPINF
AtRVE8       KNA-----PVLHGVPDFAE--V-YNFIGSVFDPETRGHVEKLKEMDPINF
OsRVE5       KRA-----PTLRAMPDFAQ--V-YNFLGSIFDPETSGHLQRLREMDPIDV
AaRVE        KSV-----ASLRATPDFAQ--V-YKFIGSVFDPGTAGHLKKLKEMAPIDR
```

```
SmRVE        KPG-----VTLSAAPAFSE--V-YKFIGSIFDPGTAGHLKKLREMAPIDR
OsRVE4       KNE-----PSLRLMPDFAQ--V-YSFLGSVFDPSTSGHLQKLKEMNPIDV
AtRVE6       KNV-----HSLRVLPDFAQ--V-YGFIGSVFDPYASNHLQKLKKMDPIDV
PpRVE1       KCV-----SCIRAAPDFAE--V-YKFIGSVFDPIVSGHLRTLKEMAAIDR
PpRVE2       KTA-----SCIRAAPDFAE--V-YKFIGSVFDPGVSGHLRKLKEMSAIDR
PpRVE3       KCG-----SCIRAAPDFTE--V-YKFIGSVFDPGVSGHLRKLKEMSPIDR
KfCCA1       GNM-----AAAVAAATMAAASAWWTLQNNAQFP-QAKESEEQK-G--SMG
OsCCA1-2     TAI----IMAAVTAATVAAASAWWTLHGAPLHPG-SGNSSPL-KTEDGNE
OsCCA1       LSSGIH--MASIVTATVAAASAWWATQGLL-PPP--ADVQRAQEKQDNDE
AtLHY        NNG-----ITAIAAATVAAATAWWASHGLV-API--VAVPTPAMTQDQDS
AaCCA1       GSF---Y-AAAVAAATIAAASAWWALQGSPVHP-APTRTQKQE--DEGVP
AtCCA1       SGT-----LAAMAAATVAAASAWWAANGLL-APL--HPSTFGPSCQEHDS
OsRVE2       STA---------PNGQVHSALPCFTYHNEF-NPQ----------------
OsRVE1       GAA----------GFAAQPVMPWLSYNGSCF----------------AAA
AtRVE7       VPY-----------------------------------------------
AtRVE7Like   VPY-----------------------------------------------
PpCCA1a      KI------AVSAMAATIAAASTWWAMHGGPMHPAV--------QCGENIP
PpCCA1b      RSF-----AVSAMAATIAAASAWWAMQGGPMHPAA--------QCGQEVL
OsRVE3       NSG-----GESAADARIPPLHVWWPYYGFI-HPR-----------SDESS
AtRVE2       IP------------------------------------------------
AtRVE1       NE----------LS--SSKTL-----------PI-RNSQEELLCW---PL

OsRVE6       -----LP--LSP--------------------------------------
OsCCA1-3     EAIVL----------------------------------QS---------
PaCCA1       ESNDARC---HAEKQ-----------------GKERSQCASNTPRRGAF-
KfRVE        ETIILLMRNLAINLASPVFAEQQHILALAD--QPRM-IEPESQ----EQ-
MpRVE        ETVLLLMRNLSINLSSPEFEERKLFMPAFE--QHLA-MLSAQ--PHRTH-
AtRVE5       ETVLLLMQNLSVNLTSPEFAEQRRLISSYS--------------------
AtRVE3       ETVLLLMKNLSVNLTSPEFDEQRKLISSYN--------------------
AtRVE4       ETVLLLMRNLTVNLSNPDFEPTSEYVDAAE--------------------
AtRVE8       ETVLLLMRNLTVNLSNPDLESTRKVLLSYD--------------------
OsRVE5       ETVLLLMKNLSINLTNPNFEAHRKVLASHG---------------HE---
AaRVE        ETVLLLMRNLSINLSSPDFEEHKLFLSVYD--QING-PSTAPAPSKHRY-
SmRVE        ETVLLLMRNLAINLSSPDFDQRVKFADK-------------------CF-
OsRVE4       ETALLLMRNLSINLTSPDFEDQKKLLSSYS-----------------EL-
AtRVE6       ETVLLLMRNLSINLSSPDFEDHRRLLSSYD----------------TDH-
PpRVE1       ETVLLLMRNLSINLSSPDFDEHKLFMPVID--DSTS-IPGAPQPTPRSY-
PpRVE2       ETVLLLMHNLSINLASPDFEEH----------------------TARSH-
PpRVE3       ETVLLLMRNLSINLASPDFEEHKLYLSVYV--DLTS-APSAAQ-TPRSY-
KfCCA1       APGGVPR--EAAIRRAERANKLREL-----KGLRTQSASGSNEPRGAEG-
OsCCA1-2     ESCDVEM---QIENQ-----------------IKERSSSGSNTPWEGAF-
OsCCA1       S--EVSL---HTEL------------------KQDRSSCGSNTPWEGAF-
AtLHY        D--VTKL---NADS------------------LVDRSSCGSNTPWEGAF-
AaCCA1       DEAPSRK--CSAAKRAERASRMKEQ-------ARVRSVSGSNTPRGGDG-
AtCCA1       E-----V---ENKS------------------QVDRSSCGSNTPWEGAF-
OsRVE2       --------------------------------QREGSWTESNTSY-----
OsRVE1       -----------------AANQQCHR-------QREGSLTGSNTA------
```

```
AtRVE7       ----------------------------------ETFCTGSNAFW-----
AtRVE7Like   ----------------------------------FCTGS---S-------
PpCCA1a      INGN-SR--SSAMRRAERAQLVKEL-------SSNSSACGSSDGRRGAFL
PpCCA1b      SNNNHSR--SSAMRRAERAQRVKEL-------SSNSSACGSSDGRRGAFL
OsRVE3       S--------------------------------------------S--L-
AtRVE2       --------------------------------SETQSQCSSSTS------
AtRVE1       KQEDVEN-------------------------QNEGSSTGSNTG------

OsRVE6       ------FEPIRRLLS
OsCCA1-3     --SGAGFVPYKGCQK
PaCCA1       -KSGVGFVPYERCS-
KfRVE        ----------P----
MpRVE        -GFGMPLPTLT----
AtRVE5       ---------------
AtRVE3       ---------------
AtRVE4       -----------S---
AtRVE8       -----------A---
OsRVE5       -----------K---
AaRVE        -VYGLGMPPLA----
SmRVE        -----------KVRK
OsRVE4       ----------P----
AtRVE6       ---------------
PpRVE1       -VYSLPLPSLP----
PpRVE2       -VYS--LPSLP----
PpRVE3       -VYSAPLPSLP----
KfCCA1       HGGSSGFVPYKGYQG
OsCCA1-2     -NFFQGSVTWETLCF
OsCCA1       -SRRTGFKPYKRCSV
AtLHY        -TRQTGFKPYKRCSM
AaCCA1       -FSGVGFVPYQRNST
AtCCA1       -RGRTGFKPYKRCSM
OsRVE2       -NCRRGFVPYKRCVA
OsRVE1       -LLQRGFMPYKRCAA
AtRVE7       -VNASGFRPYKRCLS
AtRVE7Like   -VNASGFRPYKRCLS
PpCCA1a      -YSGVGFVPYQRVSI
PpCCA1b      -YSGVGFVPYQRAST
OsRVE3       -EVIRGFVPYKRCKF
AtRVE2       -NNMRGFMPYKKRVK
AtRVE1       -SNSRGFGPYKKRKM

;
end;
```

(b)

```
#NEXUS
BEGIN DATA;
dimensions ntax=25 nchar=361;
format missing=?
symbols="ABCDEFGHIKLMNOPQRSTUVWXYZ"
interleave datatype=PROTEIN gap=- match=.;

matrix
KfTOC1      M-----------HDVTTFHRSSVHILLADRDLQTRKEVFELLRRCSYKVT
PaPRR1      M-----------AGSSVLDRSCVRILLCDKDPTNSQQLLELLRKCMYQVT
MpTOC1      M-----------GRRVVFDRSRLRILLCDPNTSP--EVVDLLQRCSYQVV
SmTOC1      M-----------EGKIGFDRSRVRILLCDKDSATAQEVKELLCRCSYQVS
AtTOC1      M-------------DGFIDRSRVRILLCDNDSTSLGEVFTLLSECSYQVT
OsPRR1      MV----------EGQQFVDRSKVRILLCDSDPSSSREVLRLLCNCSYQVT
OsPRR59     --------------------------------------------------
OsPRR95     MG----------RETRMLPRMPVRVLLAEGDDSTRHIICALLRKCGYRVA
AtPRR7      MN----------CWERFLHVRTIRVLLVENDDCTRYIVTALLRNCSYEVV
OsPRR37     MM----------CWERFIQKKTIKVLLVDSDDSTRQVVSALLRHCMYEVI
OsPRR73     ------------RWERFLPVKTLRVLLVENDDSTRQVVSALLRKCCYEVI
PaPRR3      MQ----------VKDQHQPQRCLKVLLVEDDDSTRKVVRALLCHCRYEVT
AtPRR5      MW----------KWERFLPKIALRVLLVEADDSTRQIIAALLRKCSYRVA
KfPRR       MT----------SWQATLPSKHLRVLLVEDDDCTRHVVAALLRNCGYEVT
AtPRR9      MG----------QWEKYLPKTVLRVLLVESDYSTRQIITALLRKCCYKVV
SmPRR7b     M-----------QAENAFQHHHLKVLLVEDDDSTRHVVAALLRNCGYEVT
AtPRR3      MC----------KWERYLPVRSLKVLLVENDDSTRHIVTALLKNCSYEVT
SmPRR7a     MT----------GWENLPPYRQLKVLLVEDDDSTRHVVAALLRNCGYQVV
PaPRR7      MW----------RWSLLVHLQPLVD-----------VV---------SVV
AaPRR       MT----------GWESFLPQRHLRVLLVEDDDSTRHVVGALLRNCSYEVT
MpPRR       MS------AEEQGWESFLPRKHIRVLLAEDDDSTRHVVGALLRNCGYEVT
PpPRR4      MDVARAGE----GWESFLLKRNLKVLLVEDDDATRHVVGALLRNCNYEVT
PpPRR3      ---------------------MLSLLL-N-------------------VE
PpPRR1      ------------GWESFLLKRSLRILLVEYDDATRHVVGALLRNCDYEVT
PpPRR2      ------------SWESFLLKRKLRVLLVEDDDATRHVVGALLRNCNYEVT

KfTOC1      AVESAKHVLEVLRAGGPAVDVILSEVEFPHGKGLKLLKHIMREEKLKQIP
PaPRR1      AVSTARGVVSILNAEGQEIDLILADVDLPKSKGFKMLKYITRSTCLQRIP
MpTOC1      AVQNARQVMEVLRNGMSDVDLILAEVELPKKKGFKMLKHITKEERLKQIP
SmTOC1      VVKTARQVVEVLNITDSKVDLVLSEVELPNGRGFKMLKHIVKSENFKHIP
AtTOC1      AVKSARQVIDALNAEGPDIDIILAEIDLPMAKGMKMLRYITRDKDLRRIP
OsPRR1      CAKSPRQVINVLNCEAGEIDIILAEVDLPVSKCFKMLKYIARNKELRHIP
OsPRR59     -----MKAWGVMRERAYAFDLVLTEVTMPTLSGIELLSRIVASDECKNIP
OsPRR95     AASDGVKAWDILKEKSFNIDLVLTEVELPLMSGFLLLSTIMEHDACKNIP
AtPRR7      EASNGIQAWKVLEDLNNHIDIVLTEVIMPYLSGIGLLCKILNHKSRRNIP
OsPRR37     PAENGQQAWTYLEDMQNSIDLVLTEVVMPGVSGISLLSRIMNHNICKNIP
OsPRR73     PAENGLHAWQCLEDLQNHIDLVLTEVVMPRLSGIGLLSKITSHKICKDIP
PaPRR3      AVANGLQAWDLLEDTRNQFDLVLTEAMGPCLSGIDLLSKIMSHRTYKHIP
```

```
AtPRR5      AVPDGLKAWEMLKGKPESVDLILTEVDLPSISGYALLTLIMEHDICKNIP
KfPRR       PAADGFQALKLLSDKMKQFDLVLTEVVMPGLSGIQLLSRIQQRDQHKRMP
AtPRR9      AVSDGLAAWEVLKEKSHNIDLILTELDLPSISGFALLALVMEHEACKNIP
SmPRR7b     PAASGLQAWEILETRSSSVDLVLTDLMMPRLSGIELLGKIMSRDSPKRIP
AtPRR3      AVPDVLEAWRILEDEKSCIDLVLTEVDMPVHSGTGLLSKIMSHKTLKNIP
SmPRR7a     PAANGLQAWSLLDDRNREIDLVLTDVVMPGLSGLGLLSKIMHHKNHQKVP
PaPRR7      AVANGQQAWKLLEDPSNHFDLVLTEVVMPCLSGIGLLCKIMGRSTCKNIP
AaPRR       SAGNGLQAWELLETPSNHFDLVLTDVVMPCLSGVGLLSRIMSLDPGKRVP
MpPRR       SAANGMQAWEMLEDPNNFFDLVLTDVVMPCLSGVGLLTKIMSREAGKRIP
PpPRR4      PVANGSLAWGLLEEANSNFDLVLTDVVMPYLSGVGLLSKMMKREACKRVP
PpPRR3      PR-------PELEDANSNFDLVLTDVVMPCLSGVGLLSKMMKREACKRVP
PpPRR1      AVANGSIAWGLLEDANSNFDLVLTDVVMPRLSGVGLLSKMMKREACRRVP
PpPRR2      SVANGSLAWGLLEEANSNFDLVLTDVVMPCLSGVGILSKMLKREACKRVP

KfTOC1      VVMMSQRDEMALVVKSLRLGAADYLVKPLRTNELLNLWTHMWRRRRMLGM
PaPRR1      IVMMSARDEVTVVMKCLKLGAADYLVKPLRINELLNLWMHMWRRRRMLGL
MpTOC1      IVMMSSRDEMAVVVKCLRLGAADYLVKPLRTNELLNLWTHMWRRRRMLGM
SmTOC1      IVMMSARDEMAVVVKCLKLGAADYLVKPLRINELLNLWTHMWRRRRMLGL
AtTOC1      VIMMSRQDEVPVVVKCLKLGAADYLVKPLRTNELLNLWTHMWRRRRMLGL
OsPRR1      IIMMSNRDEVSVVVKCLRLGAAEYLVKPLRMNELLNLWTHVWRRRRMLGL
OsPRR59     VIMMSSQDSIGTVLRCMQKGAVDFLVKPVRKNELRNLWQHVWRRHAMNSQ
OsPRR95     VIMMSSNDSVSMVFKCMLKGAADFLVKPIRKNELRNLWQHVWRKQLSSGV
AtPRR7      VIMMSSHDSMGLVFKCLSKGAVDFLVKPIRKNELKILWQHVWRRCQSSSG
OsPRR37     VIMMSSNDAMGTVFKCLSKGAVDFLVKPIRKNELKNLWQHVWRRCHSSSG
OsPRR73     VIMMSSNDSMGTVFKCLSKGAVDFLVKPIRKNELKNLWQHVWRRCHSSSG
PaPRR3      VIMMSSHDSMILIFKCLTMGAVDFLIKPLRKNELKNIWKHIWKRHQNSSA
AtPRR5      VIMMSTQDSVNTVYKCMLKGAADYLVKPLRRNELRNLWQHVWRRQTSLAP
KfPRR       VIMMSSKDATDVVFKCFQRGAADFLVKPVRKNELKNLWQHVWRRCKSSSG
AtPRR9      VIMMSSQDSIKMVLKCMLRGAADYLIKPMRKNELKNLWQHVWRRLTLRDD
SmPRR7b     VVMMSCLDSMDVVLKCLSKGAVDFLVKPVRKNELKNLWQHFWRMCLSSSG
AtPRR3      VIMMSSHDSMVLVFKCLSNGAVDFLVKPIRKNELKNLWQHVWRRCHSVSG
SmPRR7a     VVMMSSHDSTNVVFKCLTKGAADFLVKPVRKNELKNLWQHAWRKARSSSG
PaPRR7      VIMMSSHDSMGTVFKCLSKGAVDFLVKPVRKNELKNLWQHVWRRCQSNVD
AaPRR       VVMMSSHDSMDFVFRCLSKGAADYLVKPVRKNELKNLWQHVWRKCHSSSG
MpPRR       VIMMSSHDSLDIVYRCLFKGAADFLVKPVRKNELKNLWQHVWR-CQSSSG
PpPRR4      IVIMSSYDSLGIVFRCLSKGACDYLVKPVRKNELKNLWQHVWRKCHSSSG
PpPRR3      IVVMSSYDSLDIVFRCISKGACDYLVKPVRKNELRNLWQHVWRKCRSSSG
PpPRR1      IVIMSSYDSLDIVFRCLSKGACDYLVKPVRKNELRNLWQHVWRKCHSL--
PpPRR2      IVIMSSYDSLNIVFRCLSKGACDYLVKPVRKNELKNLWQHVWRKCHSGSG

KfTOC1      TRLEGQVNEDNER-EDSRPQMS-YGSPAKFRTPPAP--------F--VPS
PaPRR1      RNHAG---PEIS-------ASH---------EL-----------------
MpTOC1      RSVMDVANSKLSQ--YLLLTSV---------KP---VLTVCTHI------
SmTOC1      KKLKPISEEE---------------------EI-----------------
AtTOC1      RSTNPQRGNLSHN--EWSVATA-DHLAGSHHEPMKR--------------
OsPRR1      ENINQETSTSNQY--ESNPSDA---------EGGDQ--------------
OsPRR59     SKTGEHSDEESDAQSSGSKREV-HALKGNDDAPSGN--------LRSSDP
```

```
OsPRR95     HKNMECSEQESDAQSSCTRSEL-FSKPDHKNTEKNG--------CEKASR
AtPRR7      LNASDGSSDGSGAQSSWTKKAV-DVTMGRDLEISIR--------ESPSSK
OsPRR37     LNARDGSDNGSGTQSSWTKRAV-DDFKGKDLEIGSP--------EESSVR
OsPRR73     HNARDGSDNGSGTQSSWTKRAV-DDSMGKYLEIGAP--------NEPTTQ
PaPRR3      SKIVDGSDKGIGTQAG-------DCAMGQKLEIAIP--------VEPLQE
AtPRR5      DHVVSG--NGGDAQSSCTRPEM----------------------QSKQAE
KfPRR       VNVGGGSDNGSGTRNESSPVPD-QRE-----ETPER--------SRSSEK
AtPRR9      -DSRYHSDQGSGAQAI----------------------------KETFDV
SmPRR7b     LNVGGGSDNGSGTQSSWTKKPV-DRDCG-EVAMAEK--------GGGEPS
AtPRR3      LNQDGGSDNGSGTQSSWTKRAS---------QIGTG--------SKKAEE
SmPRR7a     IRISDQSDTES----SCTKKVA-PKP-----CPPSR--------TDRAME
PaPRR7      LNNRDGSDNGSGTQISWTKRVV-DFAMGQDSEIAVP--------SEVATK
AaPRR       LNLRGGSDNGSGTQSSWTKRAT-EIESAHDVESVKK--------YEQCPE
MpPRR       LNVRGGSDNGSGTQTVAEHVQ--DEQMGQDLEMATR--------SGGSAK
PpPRR4      VNAQGGSDNGSGNQACMQPVQ--DGEMGHDLEMATR--------EESSPK
PpPRR3      LNAQGGSDNGSGNQACMQPVQ--DEEMGHDLEMATR--------EESSPK
PpPRR1      --------------------------------------------EESSLK
PpPRR2      LNAQGGSDNGSGNQACVQPVQ-LDSKMGHDLEMATR--------EESSPK

KfTOC1      SGWHPNGAQHAPPPEYHRSAQGDARRGTEGGE----GSWQNSHRPPYFP-
PaPRR1      ---------QK----------------------------NVSVSPEIPIG
MpTOC1      ---------PA-----------------------------CMGPPA----
SmTOC1      ---------P-----------------------------HHQHSIPWSSS
AtTOC1      SLHLHRGLAEKF----------GTEQ----YH----SQGETLQNGASYPH
OsPRR1      RLDS-------S----------TPPV----YH----FP-------FYYPG
OsPRR59     GKDLLVVAQTDRSHFVEINLEKQHHNGYTNHKLNEKDIFNHSNSSAFSRY
OsPRR95     DMELVHIIDNQQAHQLELSLRRSDYSRLESQEKNERRTLNHSTSSPFSLY
AtPRR7      QMHEDGGSSFK-LMHVEHSSKRHRGTKDDGTLRDDRNVLRRSEGSAFSRY
OsPRR37     AADLIGSMAKNMMPSLELSLKRSRSTGDGANQEEQRNVLRRSDLSAFTRY
OsPRR73     TVDLISSIARSTMTSEELGLKRLKTTGSATEIHDERNILKRSDLSAFTRY
PaPRR3      AIDLIGSIARKPTPLLELTLRRSHPISDGDG-VHEPHVLRHSGASAFSRY
AtPRR5      AIDFMGASFRRTRIELDLSLRRPNAS--ENQSSGDRPSLHPSSASAFTRY
KfPRR       AADLIGNFVEEKMPGLELSLKRGRPAGDSSEEAQQMRTLRQSGGSAFSRY
AtPRR9      TMDLIGGIDKRPGPELGLSLKRSCSVSFENQDESKHQKLSLSDASAFSRF
SmPRR7b     CKDLLASIQKQADSALELSLKRTRVKGPDDGDGEQKRLLHHSGGSAFSRY
AtPRR3      PGDLEKNAKYSVLQSLEQTLKKTRED--RDYKVGDRSVLRHSNLSAFSKY
SmPRR7a     AVDLIGNIAEPSAPTLELALKSPTP----DHAYEPSYVLNQSGPSAFSRS
PaPRR7      AADLIGAIANKPLPFLELTLKRPRQNGKEDGEPEDRHVLRQSGVSAFSRY
AaPRR       PMDLTATISRQALPTLELSLKRPRAAGDHEGETDDRRVLRQSGGSAFSRY
MpPRR       AIDLIGGIACQNMPTLELSLKRSRVPGDDDVEVEERRAVRQSGGSAFSRY
PpPRR4      IIDLINVIACQPGTSLELSLKRPRSAVGNGGELEERQPLRHSGGSAFSRY
PpPRR3      AVDLSHRRRVQSLVKCDIELGR--------------------------WY
PpPRR1      AVDLINGIACQPGSMLELSLKRPRSAVDNDGDTEERQPLRHSGGSAFSRC
PpPRR2      AIDLINVVACQPGPMLELSLKRPRSAVDNDGELEERQPLRYSGGSAFSRC

KfTOC1      -PQFAGMPGRESQLPSDLLM----PHLAMFNLP----PQSKPPQVTG---
PaPRR1      -AAAG---------SMHLC-H-GVHDV-----------------------
```

```
MpTOC1     --------------PSEQIPM--MMHTH-KETL-----------------
SmTOC1     -TGAA---------------L-MVHSIH----------------------
AtTOC1     -ERSR---------DGSGF-S-APNAYP-YYMH-----------------
OsPRR1     -DHGM---------HG----------------------------------
OsPRR59    -GNKRIELLSCEV-GAAIPYHYGAIMQPMYYPQGA-PVDL------RKGS
OsPRR95    -NCRTAS-----LTFDGQPFWNGAPVASLFYPQSA-----------KTLS
AtPRR7     -NPASNA---IKVKH--------SSFQPL------LPPQCGSSNVNYSVN
OsPRR37    -HTPVASAMKTDVKANGHTSAFHPAQH--------GAPQCGSSNVNYGVN
OsPRR73    -HTTVASALKTDVKATQHTSAFHPVQR--------SDAQCGSSNVNYSVN
PaPRR3     -NTKCNHVMPFEILYNGVPVQYGAAFPAMFHPQTCTVPNCGLSSM-ESVN
AtPRR5     -VHRPLQ-------SNQLNSWPGQS----SYPTPT-------------GL
KfPRR      -SMGGAMPSAFQMPPGFPEPGFGPPPHFGYYFQPQESGLAGSSRQS-ANN
AtPRR9     -EESKSA--------NQENI--G---------------------------
SmPRR7b    -SNNPLL-----VAFNDIAAAYAQSLHHFFYHGSSSTQQCGSSNVNGSVN
AtPRR3     -NNGATS----------------------------------------KLL
SmPRR7a    -NDQAAA-----LSYDQSLPARGGATY--YYTQPAETPGCGSTNVNGGNN
PaPRR7     -NTSGGQAVAIEMPYDTTPRAYGSAIHPIYYSHTGTPPLCGSSNMNGSAN
AaPRR      -STSVVNGLGKDMTYDGLSAAFGPSMHPGFYPRPGAGHRCGSSNVNGSVN
MpPRR      -STSGVISTAFDIIYDGVCASYGPPMHPSFYSHPA----------NGSVN
PpPRR4     -GSGGTIGMGMDLAYDGVIGTYGPAMHPMYYAHPSSAPRCGSTGVNGSMN
PpPRR3     -GSGGTIGMGMELAYDGMGGAYGPAMHPMYYAHAGGAPRCGSTGVNGSMN
PpPRR1     -----------------------------------GAPRCGSTGVNGSMN
PpPRR2     SDSGGTIGMGMDLAYDGVGGSYGPAMHPMYYAHPSGAPRCGSTGMNGSMN

KfTOC1     GPVIGVSV-MDQAERREAALSKFRQKRKDRCFAKKIRYASRKKLAEARPR
PaPRR1     ---------ISQVERREAALNKFRQKRKDRCFDKKIRYVSRKRLAEQRPR
MpTOC1     STRTTANTYFEQAERREAALNKFRQKRKDRCFEKKIRYVSRKRLAEQRPR
SmTOC1     ---------AGVAERREAALHKFRQKRKDRCYEKKIRYASRKRLAEQRPR
AtTOC1     ---QHSQMSVNKLDRREEALLKFRRKRNQRCFDKKIRYVNRKRLAERRPR
OsPRR1     ---QSNQLPCSRSERRAAALAKFRLKRKERCFDKKVRYVNRKKLAETRPR
OsPRR59    ---------DGDRSRREAALLKFRMKRKDRCFEKKVRYHSRKKLAEQRPR
OsPRR95    GSVCDSSS-LRHLSQREAALNKFRLKRKDRCFEKKVRYQSRKLLAEQRPR
AtPRR7     GSVSGSGHGENKISQREAALTKFRQKRKERCFRKKVRYQSRKKLAEQRPR
OsPRR37    GSNSGSNNGLKRFTHRVAAVIKFRQKRKERNFGKKVRYQSRKRLAEQRPR
OsPRR73    GSFSGGHNGQNGVCYREAALNKFRQKRKVRNFGKKVRYQSRKRLAEQRPR
PaPRR3     ASEHGSNNRQNQSAQREAALKKFRLKRKERCFEKKVRYQSRKRLAEQRPR
AtPRR5     QDRDCSMDVIQQSLQREAALTKFRMKRKDRCYEKKVRYESRKKLAEQRPR
KfPRR      TSGGGSNFGVDRSARREAALNKFRQKRKERNFEKKVRYQSRKRLAEQRPR
AtPRR9     --KAASKEVRWSRSQREAALMKFRLKRKDRCFDKKVRYQSRKKLAEQRPR
SmPRR7b    GSVSGSNNLSARLTRREVALYKFRQKRKERCFEKKVRYQSRKRLAEQRPR
AtPRR3     GSSSSSDNPSDRWAQREAALMKFRLKRKERCFEKKVRYHSRKKLAEQRPH
SmPRR7a    GSVSGSNHGNNSKVRREAALNKFRQKRKERCFEKKVRYQSRKRLAEQRPR
PaPRR7     GSATGSNNGINRSAQREAALTKFRQKRKERCFEKKVRYQSRKKLAEQRPR
AaPRR      GSASGSNNATTKFSRREAALNKFRQKRKERCFEKKVRYQSRKRLAEQRPR
MpPRR      GSASGSNNLQNRFARREAALNKFRQKRKERCFEKKVRYQSRKRLAEQRPR
PpPRR4     GSASGSNTGQMRFARREAALNKFRQKRKERCFEKKI--------PKQETA
PpPRR3     GSASGSNTGQIRFARREAALNKFRQKRKERCFEKKIRYQSRKKLAEQRPR
```

```
PpPRR1       GSASGSNTGQIRFARREAALNKFRQKRKERCFEKK-RYQSRKKLAEQRPR
PpPRR2       GSASGSNTGQMRFARREAALNKFRQKRKERCFEKK-RYQSRKRLAEQRPR

KfTOC1       IKGQFVRQKEG
PaPRR1       VRGQFVRQTND
MpTOC1       NRGQFVRRTSD
SmTOC1       VKGQFVRRAEE
AtTOC1       VKGQFVRKMNG
OsPRR1       VRGQFVRQANY
OsPRR59      VKGQFVSQKLK
OsPRR95      VKGQFVRQDHG
AtPRR7       VRGQFVRKTAA
OsPRR37      VRGQFVRQAVQ
OsPRR73      IRGQFVRQSGQ
PaPRR3       VRGQFVCQAVC
AtPRR5       IKGQFVRQVQS
KfPRR        IRGQFVRQATF
AtPRR9       VKGQFVRTVNS
SmPRR7b      IKTKINDNNEA
AtPRR3       VKGQFIRKRDD
SmPRR7a      VRGQFVSQAVF
PaPRR7       VRGQFVRQTTH
AaPRR        VRGQFVRQAVY
MpPRR        VRGQFVRQTVY
PpPRR4       C----------
PpPRR3       VGAKCCIAALR
PpPRR1       VRGLFVRQAAH
PpPRR2       VRGQFVRQAVY

;
end;
```

(c)

```
#NEXUS
BEGIN DATA;
dimensions ntax=11 nchar=204;
format missing=?
symbols="ABCDEFGHIKLMNOPQRSTUVWXYZ"
interleave datatype=PROTEIN gap=- match=.;

matrix
PpELF3-2     AGPALFPRLHVAETKKAGPRGPPRNKMALYEQLTIPSHRYKMSPLPPSKK
PpELF3-1     PEIALFPRLHVAETKKAGPRGPPRNKMALYEQLTVPSHKFKPSPMPPTNK
PpELF3-3     VGAALFPRLHVAETKKAGPRGPPRNKMALSEQLTIPSHKFKPSPIAPSKK
KfELF3       EAAALFPRLHVSHAKRNGPRPPPRNKMALYEQVTLQASHPKAAGASSRPR
SmELF3       PGGALFPRLHVKETKNAGPRAPPRNKMALYEQLTIPSHRFQQQQSDKTRA
```

```
AtEEC        TIPPLFPRVHVNDTGRGGL-----------------SQQFDGKTMS----
AtELF3       ILEPMFPRLHVNDADKGGPRAPPRNKMALYEQLSIPSQRFGDHGTMQRKM
OsELF3-1     VMGPLFPRLHVNDAAKGGPRAPPRNKMALYEQFTVPSHRFSGGGGGSGKR
OsELF3-2     VMGPLFPRLHVNDAAKGGPRAPPRNKMALYEQFTVPSHRFSGGGGASGRK
AaELF3       ETGALFPRLHVKETNKVGPRAPPRNKMALYEQFTIPSHRFMSSSLPAAKK
MpELF3       ETGALFPRLHVKETKRVGPRAPPRNKMALYEQFTFPSYRFVPPAVMSKKG

PpELF3-2     GKEDDDFAVPTYSSAAETESSILEDLPLGSSCFQELLDHFGQQELWKAQK
PpELF3-1     ESLGDDFAVPTYSSAMETGSSMLENVVMGSIEFRDVVDAFGQQEFWKVQM
PpELF3-3     GSRDDEFAVPAHSSGARTGSLMLENVPTDFVQLRDVVNIFGQQGFWKTQK
KfELF3       QGEEEGCIVPAYSGPCDVESNGASAAAAAGILPKDVVSVIGQHGFWKSRK
SmELF3       PRVDDVAAVPTYPSKDDSATSVVNNAAPSAITSKEIMSAVGDEEFWKMRK
AtEEC        --EKNQFS-PIYNTKVASNCSAIESLSGISASSYDIARVIGEKRFWKMRT
AtELF3       VREEEDFAVPVYINSDVSDDSMVDSISSIDVSPDDVVGILGQKRFWRARK
OsELF3-1     LADDDEFMVPSVFNSDDVSDSSVECITGWEISPDKIVGAIGTKHFWKARR
OsELF3-2     LADDDEFIVPSVFSADDLSDSSVECITAWEISPDEIVGAIGAKHFWKARR
AaELF3       HRNEDDFTVPTYSVPDDSDSSMVDSITIHRISPRHVIGAIGQQEFWRTRK
MpELF3       GRINDDCTVPIYTAPEESDATMLEADPSHKITPKDLINAVGQQEFWTARK

PpELF3-2     AIIRQQKVFSRQVFELHRVIEVQRLLAKLPSLSFRL-SNPYAARMHMYVP
PpELF3-1     MIIRQQKLFALQVFELHRAIEVQQLLAKALCFSLHV-ANPCATRMYMYVP
PpELF3-3     VIIRQQRIFSKQLFDLHRIMEVQHLLAKELCFSITV-TNPWAPRMYVYVP
KfELF3       AMQQQQKKFSAQLFEMHRLVKVQKMFAENPDFTTEQVPNPWLQNAAYY-A
SmELF3       AMQRQQTIFQKQLFELHRLTKVQHLMANSKISD----YNQWYAPL----P
AtEEC        YMINQQKIFAGQVFELHRLIMVQKMVAKSPNLFLES-GNQWLVPVLVYKP
AtELF3       AIANQQRVFAVQLFELHRLIKVQKLIAASPDLLLDE-GQQWLIPVLIYKP
OsELF3-1     AIMNQQRVFAVQVFELHKLVKVQKLIAASPHVLIES-QNQWLVPVLVYKP
OsELF3-2     AIINQQRVFAAQVFELHKLVKVQKLIAASPHVLIEG-QNQWLIPVLVYKP
AaELF3       AILRQQRIFSVQVFELHRLVKVQQLMAMSPSVEYTV-VNPWLGAMYMCHP
MpELF3       ILQRQQMIFASQVFELHCLIKVQQLLVGEDEEDWEE-ANGWFGALYMY-P

PpELF3-2     YPGPCSPWGFPWFPIIQCGVIKVVPRAVSATQESAASILLSIQKERQR--
PpELF3-1     YSGPCPPWASRWFPTLYGGVIKVVPRAVSATQESTASILLSIQTERRR--
PpELF3-3     YPPTCSPWACRWFPILQIGVIKAVPRAVSATLESAANILLSIQTEKWR--
KfELF3       FSNPAAQWELQLFPVKAAAPIKVVPRAASETPDLGVSILKSIQRER----
SmELF3       FQFPFPV---------SHKAIKVTPRAAPVTAESAAEILHSIQKERPS--
AtEEC        FPGPCPPY--------MLRAIKAVPHNSTSASESAARIFRFIQEERRDSD
AtELF3       HPGMAHTY--------VTRVIKVVPHNAKLASENAARIFQSIQEERKRYD
OsELF3-1     YSGPCPPY--------QTNVIKVVPHNSRTASESAARIFRSIQMERQRDD
OsELF3-2     YSGPCPPY--------QTNVIRVIPHNNSQTSESAARIFRSIQMERQQDD
AaELF3       SAGPLVGWPLPLFPVVQGGVVKVAPRAVVATPESAAGILLSIQQERRR--
MpELF3       YPGPQAHWALPLFPLAFPGVIKVVPRAMVATAESAAEILLSIQKQRQQ--

PpELF3-2     ----
PpELF3-1     ----
PpELF3-3     ----
KfELF3       ----
```

```
SmELF3       ----
AtEEC        HMIS
AtELF3       SSKP
OsELF3-1     ----
OsELF3-2     S---
AaELF3       ----
MpELF3       ----

;
end;
```

(d)

```
#NEXUS
BEGIN DATA;
dimensions ntax=21 nchar=87;
format missing=?
symbols="ABCDEFGHIKLMNOPQRSTUVWXYZ"
interleave datatype=PROTEIN gap=- match=.;

matrix
AtELF4       MK--GGEDPAMWENLDRNFRQVQSVLDRNRSLIQQVNDNHQSRMADNMSK
AtEFL1       MEASGVEDVEVWDTLSNGFKRAQLYLDQNRDLIQRVNENHMSRIPDNVSR
KfELF4-1     MDLSTLGISKEWPG-AANFDKVQMILDQNRLLIKEINQNHCARRTEGLSR
SmELF4-2     MESSLGAEGKAWGVFQRTFHQVQFLLDHNRLLIKEINVNQESQIPESLSR
KfELF4-2     MDLDTVFDPHEWPGFEK-FIIVQAILDHNKLLINEINLNHERRRPEGLTR
AaELF4-1     MDE----KFNAEETFHRRFKEVECMLDHNRLLISEINHNHETRIPERLTR
OsELF4-3     MEEDSGGGGKVVQVLQRNFGEVQGILEQNRVLIQEISQNHEARDADGLTR
OsELF4-2     MDGDTAEDGKVLHAFQTSFVQVQSLLDQNRVLINEINQNHESKVPGDLSR
OsELF4-1     MEGDSQVDNKLIQTFHKSFVQVQSILDQNRMLINEINQNHESRAPDNLTR
AtEFL2       MEGDVQMDGKLLQNFQKSFVQVQDILDQNRLLINEINQNHESKQADHLGR
AtEFL4       MEGDVNMDGKLLQSFQKSFVDVQDILDQNRLLINEINQNHESKQPDNLGR
AtEFL3       MEGDTQMDGKILQTFEKSFVQVQNILDHNRLLINEINQNHESKIPDNLGR
PaELF4-1     MEGDTQIDGKVMQTFQKSFGQVQNILQHNRLLINEINQNQESRTPDNLSR
PaELF4-2     MEGDAQIDGKVMQTFQKSLGQVQNILDQNRLLIKEINQNHESKMSDNLTR
PaELF4-3     MEGDAQMDSKVMQTFQKGFGQVQNILDENRLLIKEINQNQESKIPDNLSR
PaELF4-4     MEGDAQIDDKVLQTFQKSFGQVQNILDQNRLLINEINQNHESKVPDNLSR
PaELF4-5     MEGEAQIDGKVLQTFQKSFGQVQSILDQNRLLINEINQNHESKIPDNLSR
AaELF4-2     ------MDRKALSKFHGSLSQVDFVLDHNRVLIDEINRNQEEKIPESLTR
SmELF4-1     MEADAPVDRKMWAAFERGFSQVQFLLDHNRLLINEINQNQESKVPESLSR
MpELF4       MDTDAQMDTKIWAGFHQNFSQVQYLLDHNRLLINEINQNHESKIPESLTR
PpELF4       MDRERPVDRRVSSIFNNVSRQVQFLLDHNRLLINEINQNHEAKIPEGLTR

AtELF4       NVALIQELNGNISKVVNMYSDLNTSFSSGFHGTR---
AtEFL1       NVGLINEINGNISQVMEIYSDLSLNFAKKFDQ-----
KfELF4-1     NVLLIRELHSNVAKVVELYREISESFVVVFGN--VQ-
SmELF4-2     NVMLIKELNYNIKRVVELYAGLPTSFIRSFSNLKARS
```

```
KfELF4-2    NVQLIRELNENVTKVMKLYEELSQAFVQSFGK--GQK
AaELF4-1    NVTLIRELNHNILKVVGMYSDLSAVFEKSFENAKMRI
OsELF4-3    NVALIRELNTNIARVVDLYANLSGSFSRSVTASKSRA
OsELF4-2    NVGLIRELNNNIRRVVDLYADLSSLFAASSPAHKVRS
OsELF4-1    NVGLIRELNNNIRRVVGLYADLSASFARTMDAQKVRP
AtEFL2      NVGLIRELNNNIRTVASLYGDLSHSFARSVDAQKFRS
AtEFL4      NVGLIKELNNNIRRVASLYGDLSHSFARSVDAQKFRS
AtEFL3      NVGLIRELNNNVRRVAHLYVDLSNNFSKSMEANRIRP
PaELF4-1    NVGLIRELNNNITRVVDHYAGLSTSFTKSIETQKFRT
PaELF4-2    NVTLIREFNNNITRVVDLYAGLSTSFTKAIETQKFR-
PaELF4-3    NVALIRELNNNITRVVDVYAGLSTSVTKAIETQKFR-
PaELF4-4    NVALIRELNNNITRVVDLYACLSTSVTKEIET-----
PaELF4-5    NVSLIRELNNNISRVVDLYSGLSTSFTKAVETQKFRP
AaELF4-2    NVSLITELNQNIKKVVDLYSGISSSFASTFQS-----
SmELF4-1    NVVLIKELNKNIGQVVSLYSTISSSFVKSFENHKVRS
MpELF4      NVLLIRELNNNITKVVDLYASLSVSFVKLFDNMKARS
PpELF4      NVMLIRQLNSNIGKVVDLYANLSSNFS----------

;
end;
```

(e)

```
#NEXUS
BEGIN DATA;
dimensions ntax=13 nchar=176;
format missing=?
symbols="ABCDEFGHIKLMNOPQRSTUVWXYZ"
interleave datatype=PROTEIN gap=- match=.;

matrix
KfLUX-1     MSRESISKVTLGLGSGEDLTSLEQELLTLDLAGSLAGSSVEQAIAAEEAV
KfLUX-2     --KEITRRWGVGIPSPEELAPLSTRLITPVLACAFNIEGDSGASEEEVDE
AaLUX       --------MQLGVS------------------------------------
AtBOA       MGEYRIPEWEIGLPNGDDLTPLSQYLVPSILALAFSMIPERSRTIHDVNR
OsPCL1      MGDERVMEWETGLPGADELTPLSQPLVPAGLAAAFRIPPEPGRTLLDVHR
PpLUX2      --DDQVSDWTKGLPTSEELTPLSHTLISRILASAFRIKHEEPMTEEDVRR
PpLUX1      --------------------------------------------------
PpLUX3      MHGDHVVEWNPGLPTGEELNPTSKSLISLVLASGLSMKPEPLKTAADVSG
MpLUX       MANNRVREWEAGLPSASDLTPLHHSLITRVLARAFCIPTNATPSAADVLQ
PaLUX       --EDWLGKWEDELPSPEELMPLTQNLITPDLAAAFKIHPSSASASAP---
AtLUX       MGGDRVSEWEMGLPSDEDLASLSYSLIPPNLAMAFSITPERSRTIQDVNR
PaLUX2      --DDRVTEWEAGLPGPDELTPLSQVLITPELACAFSISPEPCRSQLDVSR
PaLUX2      --NDRVEEWEEGLPTPEELTPLNQSLITPELASAFSISQEAAKSSSDVLH

KfLUX-1     PGEGAQAGQAGGTEGEEQEGRGQGNKKQRLVWTAELHSRFMNAVNHLGVK
KfLUX-2     AALNTVTPSQEGMEHTSDDPPGRALKRPRLVWTPQLHKRFCDAVNHLGVE
```

```
AaLUX         ----------GGPENSNEEPNARTLKRPRLVWTPQLHKRFVDAVGHLGIK
AtBOA         ASQITLSSLR-----GPEDASGKTSKRPRLVWTPQLHKRFVDVVAHLGIK
OsPCL1        ASAATVSRLRGGADGGNGNTNNSSSKRARLVWTPQLHKRFVEVVAHLGMK
PpLUX2        ESQATIHNLFGGLMNSNDE----PLKRARLVWTPQLHKRFVEAVGHLGIK
PpLUX1        ----------GGPVNSNEEANARTLKRPRLVWTPQLHKRFVDAVGHLGIK
PpLUX3        ESRASFLDSQGGPVNSNEETNARTLKRPRLVWTPQLHKRFVDAVGHLGIK
MpLUX         ASKSTVQQLQDCP----DGNTARTLKRPRLVWTPQLHKRFVDAVGHLGIK
PaLUX         ----------GVTENVG-EEPARTLKRPRLVWTPQLHKRFVDAVAHLGIK
AtLUX         ASETTLSSLR-----GTEDLSGKTLKRPRLVWTPQLHKRFVDVVAHLGIK
PaLUX2        ASKNTMSALRSGFENSSDEHSARTLKRPRLVWTPQLHRRFVDVVSHLGIK
PaLUX2        ASIATVTALRGGPENSGEEPAARTLKRPRLVWTPQLHKRFVDAVAHLGIK

KfLUX-1       NAVPKTILQLMNVEGMTRENVASHLQSAPAPARPPHHACKYRLYLKRLAG
KfLUX-2       NAVPKTIMQLMNVEGLTRENVASHLQ-------------KYRLYLRQFQT
AaLUX         NAVPKNIMQLMNVEGLTRENVASHLQ-------------KYRLYLKRMSG
AtBOA         NAVPKTIMQLMNVEGLTRENVASHLQ-------------KYRLYLKRIQG
OsPCL1        NAVPKTIMQLMNVEGLTRENVASHLQ-------------KYRLYVKRMQG
PpLUX2        NAVPKTIMQLMNVEGLTRENVASHLQ-------------KYRLYLKRMQG
PpLUX1        NAVPKTIMQLMNVEGLTRENVASHLQ-------------KYRLYLKRMQG
PpLUX3        NAVPKTIMQLMNVEGLTRENVASHLQ-------------KYRLYLKRMQG
MpLUX         NAVPKTIMQLMNVEGLTRENVASHLQ-------------KYRLYLKRMQG
PaLUX         NAVPKTIMQLMNVEGLTRENVASHLQ-------------KYRLYLKRMQG
AtLUX         NAVPKTIMQLMNVEGLTRENVASHLQ-------------KYRLYLKRMQG
PaLUX2        NAVPKTIMQLMNVEGLTRENVASHLQ-------------KYRLYLKRMQG
PaLUX2        NAVPKTIMQLMNVEGLTRENVASHLQ-------------KYRLYLKRMQG

KfLUX-1       LPPNARMMAPPLHQGYPPPPPHRMQP
KfLUX-2       DAPAVPSSSDAVFANGPQPPRGGAHG
AaLUX         VPTDGPGANDQLFASTPLPPNLGLFP
AtBOA         LTTEEDSSSDQLFSSTPVPPQSFVND
OsPCL1        LSNEGPSPSDHIFASTPVPHA-----
PpLUX2        LSNDGPSASDHLFASMPLPPGIMLFP
PpLUX1        LPSDGPMANDQLFASTSLPSNLGLFP
PpLUX3        LSSDGPPANDQLFSSTPLPPNLGLFP
MpLUX         LSNEGPSASDPLFASAPLPPNLTQFA
PaLUX         LSSEGPSSCDQLFASTPVPPS--LFP
AtLUX         LTNEGPSASDKLFSSTPVPPQSFANE
PaLUX2        LSNEGPSLSDHLFASTPVPYNHA---
PaLUX2        LSSEGPSASDHLFASTPVP-------

;
end;
```

(f)

```
#NEXUS
BEGIN DATA;
dimensions ntax=9 nchar=1001;
format missing=?
symbols="ABCDEFGHIKLMNOPQRSTUVWXYZ"
interleave datatype=PROTEIN gap=- match=.;

matrix
CcGI         -----------------------------------------MAFVDLLLQ
CiGI         PGDDQDRSEDDLPKLRWLQGLVSSSLLRPPPSNNQQRQVEVLAYTELFGQ
AaGI         ---------MSVARPEWLLGLQSSSLFRPPPRNPHERQMEILGYLELMVQ
PaGI         ---------MSISEQKWIHGLQSSSLFRPPPQDAQQRQAEILAYVELFAQ
AtGI         ---------SSSSSERWIDGLQFSSLLWPPPRDPQQHKDQVVAYVEYFGQ
OsGI         ---------MSASNEKWIDGLQFSSLFWPPPQDSQQKQAQILAYVEYFGQ
MpGI         ---------MSFPGQKWLRGLQSSSLFRPPPESAIRKQAEILASVELFGH
SmGI         ---------MSSPQQKWLTGLKSTSLFRAPPLDLHERQTKTVAYVELFGQ
TlGI         -------------RQQWLRGLKSSSLYRPPPANRHQRQTEVLEYVELFGQ

CcGI         LSQDLFAHVIAELIRLQYPREHPILLDDVLASIVLHSPEHGHAILHVLLS
CiGI         FASELLSDDISQLVRNHYPQNSSTLLDDVLATFVLHHPEHTDAVLHALLS
AaGI         FASESFVDEMNELVRSHYPKEEACLVDDVLAAFVLHHPEHAHAVLHALLS
PaGI         FTS-EFPDDIAELVHSHYPNGEASLLDDVLAIFVLHYPEHGHAIIHPLLS
AtGI         FTS-QFPDDIAELVRHQYPSTEKRLLDDVLAMFVLHHPEHGHAVILPIIS
OsGI         FTADQFPEDIAQLIQSCYPSKEKRLVDEVLATFVLHHPEHGHAVVHPILS
MpGI         FASESFVDDIGELVRAHYPTNEHCLLDDVLATFVLHHPEHGHAVLHALLS
SmGI         FASDSFPEDIAELVRDHYPHKEPCLLDDVLATFVLHHPEHGHTILHPLLS
TlGI         FASDAFVDDIGELVRGHYPSEEVCLLDDVLATFVLHHPEHGHAILHALLS

CcGI         SLVHGNLRYSRGISPFSAFVAIFSPSSQRACPEQYELACAEVLQLLTHYN
CiGI         CIIDGSLEYSRKSPPFGSFVALFNTSTERMGLRQWALACTEILRVLTHYN
AaGI         CVIDGTLLYSKEGSPFRCFIALFSSANEKHFSEQWAVACGETLRLLTHYN
PaGI         CIIDGTLIYDKKMSPFSSFNSLFSPSSENDYSEQWALACGEILRVLTHYN
AtGI         CLIDGSLVYSKEAHPFASFISLVCPSSENDYSEQWALACGEILRILTHYN
OsGI         RIIDGTLSYDRNGFPFMSFISLFSHTSEKEYSEQWALACGEILRVLTHYN
MpGI         CVIDGTLIYNKKTPPFGSFVSLFSPSTERDFSEQWALACGETLRVLTHYN
SmGI         CVIDGTLAYSKTTPPFGSFVSVFGVSSERDLTEQWALACGEILRLLTHYN
TlGI         CVIDGTLIYDKKTPPFGSFVALFSPSSEREFSEQWALACGETLRILTHYN

CcGI         RPVQRTSS-------GRVAPERVLTQWITDCLLASPLGFRRDYFRWCGGV
CiGI         KPVHKNRQEEDTNNNERRDAFRPLTMWITDCLLAAPSIMRTNYFRWCGGV
AaGI         RPVYKSDP-------EKRVPSRLLTPWITDSLLAAPLGVRSDYFRWCGGV
PaGI         RPVYKVDR-------QDKKPLRLLTPWITDILLAAPLGIRSDYFRWCGGV
AtGI         RPIYKTEQ-------HERKPLRPLSPWISDILLAAPLGIRSDYFRWCSGV
OsGI         RPIFKVDH-------PDRKPLRPLSPWITDILLAAPLGIRSDYFRWCGGV
MpGI         RPTFRSDT-------DRRSPTRLLTPWITDSLLAAPPSIRSDYFQWCGGV
SmGI         RPIYKSES-------GRRAPKRLLTPWITDSLLAAPLGTKSDYFRWCGGV
```

```
TlGI         RPTYKAET-------EKRVPTRLLTPWITESLIAAPLGIRSDYFRWCGGV

CcGI         VGQYATSGDLHPLPSP--SGPGKHPQMLPSTPRWAVANGAAVIVSVCDDE
CiGI         TGKYAAVGALRPPTTVLGNGQGKAPQLQPSTPRWAMANGAAVILAVWDDE
AaGI         MGKYAAGVDLKPPSTVGKLGQGKQPQLLPSTPRWAVANGAAVILSVCDDE
PaGI         KGKYAAGGELKPPTTAGGRGPGKHPQLMPSTPRWAVANGAGVILSVCDDE
AtGI         MGKYAAG-ELKPPTIAS-RGSGKHPQLMPSTPRWAVANGAGVILSVCDDE
OsGI         MGKYAAGGELKPPTTAYSRGSGKHPQLMPSTPRWAVANGAGVILSVCDEE
MpGI         MGKYTAGEELRPPTTAGGRSQGKQPQLLPSTPRWAVANGAAVILSVCDDE
SmGI         LGKYAGGGDLRPPTTGDGKGHGKHPQLLSSTPRWAVANGAAVISSVCDDE
TlGI         MGKYAAGVELRPPTTAGGRGQGKHPQLLSSTPRWAVANGAAVILSVCEDE

CcGI         VARWGSVELTAAAVPALLLPPP--EVDEHLI-GLPPLEPFARLFHRYYAC
CiGI         VTRFGTADLTAAAVPALLIPPDHHDDDARAVVALPPLEPYTRLFHRYYTM
AaGI         VERYRSAHLTAAAVPALLLPPEPAALNNPLVAGLPPLEPYAQLFHRYYAI
PaGI         VTRYETANLTAAAVPALLLPPPTTALDEHLVAGLPPLEPYACLFHRYYAI
AtGI         VARYETATLTAVAVPALLLPPPTTSLDEHLVAGLPALEPYARLFHRYYAI
OsGI         VARYETANLTAAAVPALLLPPPTTPLDEHLVAGLPPLEPYARLFHRYYAI
MpGI         VSRYETADLTAAAVPALLIPPPTAQNENLLVSGLPFLEPYAHLFHRYYAV
SmGI         VLRYETADLTAAAVPALLLPPPSTSLDEHLVAGLPPLEPFARLFHRYYAI
TlGI         VMRYETADLTAASVPALLLPPPATALDQHLVAGLPPLEPYARLLHRYYAI

CcGI         ATSGATQRLLLGLLEALPLRAPDALDAAVQLVELLRSAEDYGSACQLPPN
CiGI         GAPVATQRLLLGLLDVGPPLAADAMEAAVQLIHLIRS-EDFGGPPKLPPS
AaGI         ATPEAAQRLLLGLLEAPSSWAPDALDAAVQLVVLLRSAEDLSSTFQLPQT
PaGI         ATPSATQRLLLGLLEAPPSWAPDALDAAVQLVELLRAAEDYASTMRLPKN
AtGI         ATPSATQRLLLGLLEAPPSWAPDALDAAVQLVELLRAAEDYASGVRLPRN
OsGI         ATPSATQRLLFGLLEAPPSWAPDALDAAVQLVELLRAAEDYDSGMRLPKN
MpGI         ATPGATQRLLFGLLEAPPTWAPDALVVAVALVELLRAADNYTSSVQLPRD
SmGI         ATPGATQRLLLGLLEAPASWAPDALDAAVQLVELLRAAEDYSSSFRLPEN
TlGI         ATPGATQRLLLGLLEAPPSWAPDALDAAVQLVELLRAAEDFSSSVQLPRS

CcGI         WLHLHFLRPIGVAMAKQSGAASDAAAALLFRIFSQPVLLFPTPGHAQGPL
CiGI         WLDLHFLRPIGVAMSSRAAVMASAASALLFRIFSHQTLLFPPPL-APLPD
AaGI         WLHLHFLRPIGSAMARQSGVIADAAAALLFRIFSQPVLLFPPRGHAQGVR
PaGI         WLHLHFLRAIGTALSMRVGIAADTAAALLFRTLSQPALLFPPPRLAQGVD
AtGI         WMHLHFLRAIGIAMSMRAGVAADAAAALLFRILSQPALLFPPLSQVEGVE
OsGI         WMHLHFLRAIGTAMSMRAGIAADTSAALLFRILSQPTLLFPPLRHAEGVE
MpGI         WLTIHFLRPVGAAMAQRSGTAADAAAALLYHIFSRPALLFPPPSQSQGLF
SmGI         WFRLHFLRPMGAAMTMKQGIASDAAAALLFRLFSQPALLFPPRGHAQGAQ
TlGI         WLHLHFLRPVGAAMTVRSGIAADAAAALLFRIFSQPALLFPPPGHAQGVR

CcGI         AGRFVVKEQAAAAQANDDATALGLASLLVDHGAEVEWRICALWEAAFGLL
CiGI         TSSLRMQMQVLHL-ASEEATARGLAALMCSHGPDVEWRICSVLEAAYGLT
AaGI         VIPFRDEIEMAAVQANGQATACALAALMTEHGPDVERRVCALWELSYGLQ
PaGI         VQCSGEEIEMETYQASIEATAQGVASLMCLHGPEVEWRICTLWEAAYGLL
AtGI         IQHYRKQIEVPAAEATIEATAQGIASMLCAHGPEVEWRICTIWEAAYGLI
```

```
OsGI         LHHYKRQLEVPASEATIDATAQGIASMLCAHGPDVEWRICTIWEAAYGLL
MpGI         STQSREEAKAAAAQESEVATASGLAALLTGHGIDVECHICAIWEAAYGLR
SmGI         VVQFHAQMEALATQVNEEATAKGVASLMRDHGRDVEWRICVLWEAAYGLI
TlGI         INQIQEVADAAAAQANEEATAQAVAALMCTHGLDVEWRICALWEAAYGLL

CcGI         PPQSSSQVLPLLVANLAIVPPVLSWNVLRPLLRVFEHLPPNSPSQSSLGR
CiGI         PVDSTVIALSHVGGISEVRHVVLSWNLFRPLLHLFEHLPPGGPSHACLRR
AaGI         PVDLSVVNVPQLDMVTPLQPPVLSWNLFHPLLRVMEHLRRGTRSQACLRR
PaGI         PLSSSTVDLPEIVVATPLQPPVLSWNLFQPLLRVLEYLPRGSPSEASLMR
AtGI         PLNSSAVDLPEIIVATPLQPPILSWNLYIPLLKVLEYLPRGSPSEACLMK
OsGI         PLSSSAVDLPEIVVAAPLQPPTLSWSLYLPLLKVFEYLPRGSPSEACLMR
MpGI         SLTPSSVDLPDLVLSTPLQPPVLSWNLLRALFRILSYLPPESPSQACLKR
SmGI         PLDKSVVDLPEMVIATPLQPPLLSWTLFRPFLRVLEHVPKGCQSQTCLRR
TlGI         PLNSSKVDLPELVVATPLQPPFLSWNLVRPLLRVMEYLPRETPSLVCMRR

CcGI         IFSATVDGILERTFQSDESAGARLVGMTELRALVHHLFTEPCPSPALASK
CiGI         IFSATLSSVLFRSFPLTGHPPQQTQPLQQQNDQQQALHHHHHHHHDLLSP
AaGI         VFSATVEAIVQRTFPCEENAGAKNVGMAELRAMVHCLFTEACPSPDVAAC
PaGI         IFTATVQAILQRTFPAEQSPTSKNLAVAELRTMVHSLFTESCVPIDLASR
AtGI         IFVATVETILSRTFPPESSSATKNLAMSELRAMVHALFLESCAGVELASR
OsGI         IFVATVEAILRRTFPSETSSQSKNLAVAELRTMIHSLFVESCASMDLASR
MpGI         IFSATVEAILQRTFPLDEVAGAKSVGMGELRAMLHCLFTESFLSPELAAH
SmGI         IFSATVDAILRRTFPLDDWSGVDPAGMAELRALVHCLFTEAFLGPALASQ
TlGI         IFSATVEAILQRTFPSGEVAGAQSVGMAELRAMIHCLFTESCPSPDLGSQ

CcGI         LFTRLLTSCLAYDAAISARSVIGVATQVGRKGRREVCAGGVGSIRMGNGG
CiGI         LQFPLPLHSSSSSSSFP--K---PPATVQ---LPAAWPLA-----WPPPS
AaGI         LLADALLLCLSHDAKWRGADDRGAVATFDSYLVAAMCALACEVQMVAFSR
PaGI         LLFMVITVCLSHDAVQKAKKERGAVATFGSYILAAVCAQACEVQLFSFTS
AtGI         LLFVVLTVCVSHEAQSSNVKGQGPVAAFDSYVLAAVCALACEVQLYPMIS
OsGI         LLFVVLTVCVSHQALPGCKKRQGPVATFDSYVLAAVCALSCELQLFPFIS
MpGI         LLSEALSLCLSHDASRQSGKQRGAVATFDSYVIAAVCALACEVQFFPFSP
SmGI         LLSDALTVCLSHDTLRQSNKDRGAVASFDSYLIAAVCALACEVQLCTFSA
TlGI         LLSDVLTVCLSHDTQSLVSKGRGAVAKFDSYVIAAICALACEVQLLSLPR

CcGI         VRSGEGGGQEGGSQEFPSREMGAVGDGQAFPFRETEAAGEGVTGDEMSSG
CiGI         PTHT-EHGPPPYHHHHPLHT-----HTPSVFSSPPAAENRSATQKMLATP
AaGI         STSS-FPALSNGTPVGIQQAKRVLELLERLLAEVPSLAEQSSSSDIVGDG
PaGI         PMVN-VEGPPNGFYSAVNHTRRLLGILEALLSLKPSAAGCYSSSEIIAAA
AtGI         GGGN-SKEYGAGIDSAISHTRRILAILEALFSLKPSSVGTYSSSEIVAAA
OsGI         KNGN-SNELHNSISSAILHTRRILGILEALFSLKPSSVGTYSSNEIVAAA
MpGI         AYFT-SNRFPIGVANVLEHTKRLLGLLELLLGVSPSFAGTTSTNEILGQA
SmGI         ADGT-------GVTNSAYQARRLMSVLEGLLVVEPFSPGVNSPNDLVEAA
TlGI         SMFT-PNVYPVEVAVAADHTRRVLGLLESLLALVPSSHGTSSSGEIVAAA

CcGI         SGGAQIETKSGPATGMANGAQEMTKTGGSPSFQKRKRSRESKSVRGARGV
CiGI         STLTSLPLLPPGLLFLLLLLLLQTRLPWPPSLLLSFRPYHHHHHHPPPSL
```

```
AaGI        ISAAHISGVLGKSKACLQALAGVVRCKWDSEVCTRASHALELVQACVQTM
PaGI        MVAAHVSDLLGRSKACMHALSVMMRCKWDSELCARASTVLALIDVNRKAV
AtGI        MVAAHISELFRRSKALTHALSGLMRCKWDKEIHKRASSLYNLIDVHSKVV
OsGI        MVAAHVSELFRRSRPCLNALSALKQCKWDAEISTRASSLYHLIDLHGKTV
MpGI        VGAAHMSDLLGHSRACLHSLTGIMRCKWDPGICLKASAVLSAIESNGDLV
SmGI        IVAAHISRLLGRSRACTHALTAIVRCKWDPGVSSKAASILALVDGNDKAV
TlGI        VVAAHMSGLLGRSNACIHSLTAIMRCKWDPGIQEKAASVLALVEGNGDVV

CcGI        KGAARARRGGLTSC-AAFSMSRSRSGSPLQLGADADSACQD-KVGALMLL
CiGI        LLLLLLAAAGGGGA-VDSGVVAESGGGSVNASDVAMLLCRNWPGGSLMAL
AaGI        VEVTSSAPTSNTEP-HPSGLGKGVGGPSSRLGALRSQSSMQWSSGPIRVI
PaGI        ASIIDKSESSVSHV-E-DVSGKVIANLSMDGSDVVSLLTVDNVGNFVTAV
AtGI        ASIVDKAEPLEAYLTRDEGSEKGIKDFLLDASDLANFLTADGTQKLLRSV
OsGI        TSIVNKAEPLEAHL-ELNGSGKAIASLQVEASDLANFLTMDGSQTLLRSV
MpGI        AIATHGRNGFGGPD-GPNGVGEARGSFPLEASDVASLLCKDTLDTSLKSV
SmGI        EAVFNYADKLSGDE-AISGDGVKDASLSLNASDVTNLLCG-TVSDLLKAV
TlGI        ATAVHASNEYMSKD-RINGMGKEIANLPLDAADMASLVCKDNVSASLKLV

CcGI        LMSQQDTAVAAVPLLWQRLVRLPEVSVSQEGTSARRGWRQVAEALCNLVV
CiGI        LMEKQQLALAAVPLLWQRLMCAPEVPLSTEGTSAEKGWRQVLEGLCNIVV
AaGI        LTEKDELVISSVPVIWRRLLLAPDSTNIMDGASPRQGWRQVVEALCKLVE
PaGI        LEEKQDLCVASVPLLWQRLITAPEMQMSVESTSAKQGWRQVVDALCNIVL
AtGI        LAEKPELSFSVVSLLWHKLIAAPEIQPTAESTSAQQGWRQVVDALCNVVS
OsGI        LSEKQELCFSVVSLLWQKLIASPEMQMSAESTSAHQGWRKVVDALCDIVS
MpGI        LVGKQHLAIGATALLCQRLILAPDIPNAIEGTSAERGWGQVVEALCNISA
SmGI        LKQKRDLAVAIVPLLWQRLMSAEELPTSKEGTSAKQGWRQVVDAVCNVVL
TlGI        LTEKWQLAVAAVPLLWQRLIFAPEIPVSSEGTSAKRGWRQVVEAICNVVV

CcGI        AFPGKAASAILAQAERDLKPWMPEVATAASPDWSVNTRIMSLLAEILQQR
CiGI        AFPLQAAVAIVAKAEGELRPWEAEEDVGGDGVCRGNVRIVSLLAEMLRLR
AaGI        TFPGKATSAILLQVQRDLSSWGDQSDMRVENVRKVNRRIVTILVELLRLK
PaGI        ASPAKAATAIVVQAERDLQPWVARHDSQGQQIWRLNQRIVSLLAELLRYH
AtGI        ATPAKAAAAVVLQAERELQPWIAKDDEEGQKMWKINQRIVKVLVELMRNH
OsGI        ASPTKASAAIVLQAEKDLQPWIARDDEQGQKMWRVNQRIVKLIAELMRNH
MpGI        AFPGKVARFVVSQAEAELRPWTVKDDGPSQSLWRINTRVVCLLSDLLRLT
SmGI        TYPEKATSVVLLQAERGIQPWIIGDG--GEEKWRMNTRIVFLLSELLRLN
TlGI        GLPAQAAAAIVVQAERDLHPWTGRDDLQVQTVWRVNTRILSLLTELLRLH

CcGI        RSREAVTAVACESSLLLRATDGIALAGVEGTLPQLELLEAVSVAARSAQA
CiGI        QCPAAVGVVAHAHALLHRATAGIGIEGEACTLPQLELLEAAATAADVARH
AaGI        HCPEAIQLAAKSSDLLLRATDGLELEEDAYVLPQLELLEAANIAVQRSLS
PaGI        NAPEALMVLANASDLLMRATDGMLVDGEACTTPQLELLEAMAVTAQLSLG
AtGI        DRPESLVILASASDLLLRATDGMLVDGEACTLPQLELLEATARAIQPVLA
OsGI        DSPEALVILASASDLLLRATDGMLVDGEACTLPQLELLEVTARAVHLIVE
MpGI        HLPEIVKVVVHDGALLRRATDGMTLDGEACKLPQLELLEAAALAAQAALK
SmGI        D-PQVLGLIANAGTLLYQATDGMSVDGEPCTLPQLELLEAIAMAIKSLCA
TlGI        TSLEAVKVVVQSSDLLHRATDGMVTEGEACTLPQLELLEAAAMAVQVLLR
```

```
CcGI        WPIGGCQVAGKLLDLLKVRIPSLVKCVCHENAHVRALATSLLQTILDTPD
CiGI        WE-GGHSVTNELLFMLKERLPVTVRCLSSSSTHVRALAMALLRHLAYEAV
AaGI        WDSNSGVGAQKLVAVLKDRIPGAVRCLSHCCVDARVLSQTFLQDLRVADS
PaGI        WGVPGKAMAEGLWNLLKYRLPATVQCLSHSSAHVRALSTSVLRDILHAES
AtGI        WGPSGLAVVDGLSNLLKCRLPATIRCLSHPSAHVRALSTSVLRDIMNQSS
OsGI        WGDSGVSVADGLSNLLKCRLSTTIRCLSHPSAHVRALSMSVLRDILNSGQ
MpGI        WEEPNEEVADQLFSLLRERLPATVRCLSHNSTHVRAMSVARLRDMLYMES
SmGI        WKVSS----RGLLILLKERLPAIVRCLSHDSPRIRASSASLLREIVSTDV
TlGI        SEVSGKNALDKLLLLLKDRLPAIVRCLSHSSAHVRALSMALLRDMLYAES

CcGI        SPMAASWQAAVERAVSLEVSDRQRHGLEVRILEEVSFALGCNT-C-----
CiGI        LAARCEWRRAVEQCLSWEAHFRRSGGLSTTLLVDAASTLGCQIAV-----
AaGI        VRLLDSWKRAVEEALFMEAQHRRAARLSLTSLAAAATVLGSTIPN-----
PaGI        LNFVQDWNKAVEQCLAWEAHNRQARGMSIALLALAANALGFSAN------
AtGI        IPISIDWKADIQNCLNWEAHSLLSTTMPTQFLDTAARELGCTISL-----
OsGI        INSIINWQADVERCIEWEAHSRRATGLTLAFLTAAAKELGCPLT------
MpGI        LRSTGNWRRYVEQCVVWEKFGSSASS-TIQIVEEYLSRFGTSVWIWWEKL
SmGI        LRAAGAWLEDVEQSIAWETHYRRAEGLSESFLASAAIALGCKLPP-----
TlGI        LQGEDDWRRAVEKCVEWEAYYRRATGMSTSLLANAASSLGCSLP------

CcGI        -
CiGI        -
AaGI        -
PaGI        -
AtGI        -
OsGI        -
MpGI        I
SmGI        -
TlGI        -

;
end;
```

(g)

```
#NEXUS
BEGIN DATA;
dimensions ntax=12 nchar=559;
format missing=?
symbols="ABCDEFGHIKLMNOPQRSTUVWXYZ"
interleave datatype=PROTEIN gap=- match=.;

matrix
KfZTL       MEDRIEVLHSSPHGLIVTDALAEDHPITYVNTIFQYYTGYDAESILGRNC
AtFKF1      HAIVGMFYPMTPPSFIVSDALEPDFPLIYVNRVFEVFTGYRADEVLGRNC
OsFKF1      GDRDEAAAEGRAAAIVVSDAVEVDFPVIYVNAAFEAATGYRADEVLGRNC
```

```
AaZTL       MDGRVKMLQTSPCALVVTDALELDQPVIYVNSIFEHATGYKAEEILGRNC
AtLKP2      MEWPVGSLGTAPCGFVVSDALEPDNPIIYVNTVFEIVTGYRAEEVI----
SmZTL       --------MLGPCSVVVTDALDVDFPIIYVNNIFEFITGYKAEEVLGRNC
OsZTL1      MEWAIEGV--GACGLVVSDALEPDFPIIYVNRGFEDATGYRAEEVLGRNC
OsZTL2      MEWAIEGMASGPCGLVVTDALEPDCPIIYVNCGFEEATGYRAEEVLGRNC
PaZTL       MEWAMDNLHSTPCGFAVTDALEPDQPIIYVNAVFEYVTGYKAEEILGRNC
AtZTL       MEWPVGNLHTAPCGFVVTDAVEPDQPIIYVNTVFEMVTGYRAEEVLGGNC
MpFKF1      --MRLEVLHSSPCGLIVTDALEPDQPIIYVNTVFEFITGYKAEEILGKNC
TlZTL       MDWRLEMLHSSPCGLTVTDALEPDQPIIYVNTVFEFITGYKAEEILGRNC

KfZTL       RFLQMRGEFADKRHPAVDLKTVRKMREAIAAGQEFKGELLNFKKDGTPLI
AtFKF1      RFLQYRDPRAQRRHPLVDPVVVSEIRRCLEEGIEFQGELLNFRKDGTPLV
OsFKF1      RFLQFRDPRAQRRHPLVDPMVVSEIRRCLNEGIEFQGELLNFRKDGAPLY
AaZTL       RFLQNRGPFAQRRHPLVDATTISEIRRCISEGVEFEGELLNFRKDGTPLI
AtLKP2      ------GPFTKRRHPMVDSTIVAKMRQCLENGIEFQGELLNFRKDGSPLM
SmZTL       RFLQFRGPFAQRRHPLVDSATVTEIRRCMREGIEFWGELLNFRKDGTPLM
OsZTL1      RFLQCRGPFAKRRHPLVDTTVVTDIRRCLEEGTVFQGDLLNFRKDGSPFM
OsZTL2      RFLQCRGPFAQRRHPLVDAMVVSEIRKCIDNGTEFRGDLLNFRKDGSPLM
PaZTL       RFLQYRGPFAQRRHPLVDSMVVSEIRRCLDEGVEFQGELLNFRKDGTPLM
AtZTL       RFLQCRGPFAKRRHPLVDSMVVSEIRKCIDEGIEFQGELLNFRKDGSPLM
MpFKF1      RFLQCRGPFAQRRHPLVDPTTVATIKRCLTEGVEFRGELLNFRKDGTPLM
TlZTL       RFLQYRGPFAQRRHPLVDSTVVSEIRRCIRGEVEFRGELLNFRKDGTPLI

KfZTL       NNLLMTPIHGEDGIVTHFIGIQSFRPIKMDLGPLPERPWKDSRHNPAVFA
AtFKF1      NRLRLAPIRDDDGTITHVIGIQVFSETTIDLDRVSYPVFKHHEDFCGILQ
OsFKF1      NRLRLIPMHGDDGFVTHVIGIQLFSEANIDLSNVSYPVYKQSSEYCCILQ
AaZTL       NKLFLTPILDDEGAVTHVIGVQSFAEAKIGMGPLSNAILKERSSTCGLLQ
AtLKP2      NKLRLVPIREEDE-ITHFIGVLLFTDAKIDLGPSPDLSAKESRGLCGIFE
SmZTL       NKLCLKPIRGEDGRITHIIGIQSFSEVKLDLGPLPPPLWRNSKDRCGILR
OsZTL1      AKLQLTPIYGDDETITHYMGMQFFNDSNVDLGPLSVSTTKECSEHSDLFL
OsZTL2      NKLHLTPIYGDDETITHYMGIQFFTNANVDLGPLPGSLTKECREYSSLFQ
PaZTL       NRLRLTPIHGDDGIITHIIGIQLFTEANIDLGPMPLPFYKECREICGIFQ
AtZTL       NRLRLTPIYGDDDTITHIIGIQFFIETDIDLGPVLGSSTKESRGMCGLFQ
MpFKF1      NKLCMTPIHAEDGTITHVIGIQSFTEAKLDLGPIP---WKDSKDACGLLQ
TlZTL       NKMCLTPIHGADGVITHIIGIQSFMEAKLDLGPLPCPSWKDSRDSCGLLH

KfZTL       LSDEVLVSRILGRLAPKDVAICSMVCRRFRRLGQDDYIWKRVCRNSWGHN
AtFKF1      LSDEVLAHNILSRLTPRDVASIGSACRRLRQLTKNESVRKMVCQNAWGKE
OsFKF1      LSDEVLAHNILSRLSPRDVASIGSVCTRMHELTKNDHLRKMVCQNAWGRD
AaZTL       LSNEVLSYRILALVSPRDVASIGLVCRRLHQLTKNDDLWKLVCQNAWGAD
AtLKP2      LSDEVIAIKILSQLTPGDIASVGCVCRRLNELTKNDDVWRMVCQNTWGTE
SmZTL       LSDEVLVQKILAQLTPRDVSSVALVCRRFNEMTKNTDLWRLVCRNAWGLE
OsZTL1      LSDEVLCQKILSRLSPRDIASVNSVCKRLYHLTRNDDLWRMVCQNAWGSE
OsZTL2      LTDEVLCQSILSRLSPRDIASVSSVCRRLYLLTRNEDLWRMVCQNAWGSE
PaZTL       LSDEVLAQKILSRLTPRDVASVGSVCKRLYQLTKNEDLWRMVCQNAWGSE
AtZTL       LSDEVVSMKILSRLTPRDVASVSSVCRRLYVLTKNEDLWRRVCQNAWGSE
MpFKF1      LSDEVLTHKIIAYVAPRDVAALGLVCRRLHEITKNDNLWRSVCQNSWGFE
```

```
TlZTL        LSDEVLAQKILALVAPRDVAAIGSVCKRLHELTKNEDLWRMVCQNAWGSE

KfZTL        TAAAIQAALDTPSLGWARIARELTTLEAAAWRKFTVGGSVEPSRCNFSEC
AtFKF1       ITGTLEIM--TKKLRWGRLARELTTLEAVCWRKFTVGGIVQPSRCNFSAC
OsFKF1       VTVRLEMS--TKMLGWGRLARELTTLEAASWRKFTVGGRVEPSRCNFSAC
AaZTL        ATMSLQALPGTTSVAWGRLARELTSLEAVAWRKLTVGGAVEPSRCNFSAC
AtLKP2       ATRVLESVPGAKRIGWVRLAREFTTHEATAWRKFSVGGTVEPSRCNFSAC
SmZTL        TTAVLERVHNPRSIDWGMLARELTTLEAAAWRKLKVGGAVEPSRCNFSAC
OsZTL1       ATQVLETVAGTRSLAWGRLARELTTLEAVTWRKLTVGGAVEPSRCNFSAC
OsZTL2       TTRALETVPAAKRLGWGRLARELTTLEAVAWRKLTVGGAVEPSRCNFSAC
PaZTL        TTRVLETVPGAKRLGWGRLARELTTLEAAAWRKLTVGGAVEPSRCNFSAC
AtZTL        TTRVLETVPGAKRLGWGRLARELTTLEAAAWRKLSVGGSVEPSRCNFSAC
MpFKF1       ATKALESVPGADNLEWGRLARELTTLEAAAWRKLTVGGAVEPSRCNFSAC
TlZTL        ATAMLEAVPPAGRIGWGQLARELTTLEAAAWKKLTVGGGVEPSRCNFSAC

KfZTL        AVGNKLVLFGGEGVNMQPMNDTFVLDLSLEHPAWRHVDVSAAPPGRWGHT
AtFKF1       AVGNRLVLFGGEGVNMQPLDDTFVLNLDAECPEWQRVRVTSSPPGRWGHT
OsFKF1       AVGNRLVLFGGEGVNMQPMDDTFVLNLESAKPEWRRVKVSASPPGRWGHT
AaZTL        AVGNKLVLFGGEGANMQPMNDTFVLDFSVPHPEWIRIEVGSPPPGRWGHT
AtLKP2       AVGNRIVIFGGEGVNMQPMNDTFVLDLGSSSPEWKSVLVSSPPPGRWGHT
SmZTL        AVGNKVVLFGGEGVNMQPMNDTFVLDLSAACPEWRHVDVGSAPPGRWGHT
OsZTL1       AAGNRVVLFGGEGVNMQPMNDTFVLDLNASKPEWRHINVRSAPPGRWGHT
OsZTL2       AVGNRVVLFGGEGVNMQPMNDTFVLDLNASNPEWRHVNVSSAPPGRWGHT
PaZTL        AVGNRLVLFGGEGVNMQPMNDTFVLDLSAANPEWRHVNVSSPPPGRWGHT
AtZTL        AVGNRVVLFGGEGVNMQPMNDTFVLDLNSDYPEWQHVKVSSPPPGRWGHT
MpFKF1       AVGNKLVLFGGEGVNMQPMNDTFVLDLSVKHPAWQHVNVKSPPPGRWGHT
TlZTL        AVGNKLVLFGGEGVNMQPMNDTFVLDLSVAKPEWRHVHVSSPPPGRWGHT

KfZTL        LCCLNGSWLVVFGGCGTDGPLNDVFVLDLDAEHPAWREVAGAGPPLPRSW
AtFKF1       LSCLNGSWLVVFGGCGRQGLLNDVFVLDLDAKHPTWKEVAGGTPPLPRSW
OsFKF1       LSWLNGSWLVVFGGCGQQGLLNDVFVLDLDAKQPTWREVASEGPPLPRSW
AaZTL        LSCVNGSWLVVFGGCGKDGLLNDVYVVDLDEPHPTWRKVAGAVPPVPRSW
AtLKP2       LSCVNGSRLVVFGGYGSHGLLNDVFLLDLDADPPSWREVSGLAPPIPRSW
SmZTL        LSCLNGSWLVVFGGCGRQGLLNDVFVLDLDAKQPSWREVAGVGPPVPRSW
OsZTL1       LSCLNGSRLVLFGGCGRQGLLNDVFMLDLDAQQPTWREIPGLAPPVPRSW
OsZTL2       LSCLNGSLLVVFGGCGRQGLLNDVFTLDLDAKQPTWREIPGVAPPVPRSW
PaZTL        LSCLNGSWLVVFGGCGRQGLLNDVFILDLDAQQPTWREVAGSAPPLPRSW
AtZTL        LTCVNGSNLVVFGGCGQQGLLNDVFVLNLDAKPPTWREISGLAPPLPRSW
MpFKF1       LSCLNKSWLVVFGGCGRDGLLNDVFIVDLDAKQPIWREIGGAVAPIPRSW
TlZTL        LSCLNDSWLVVFGGCGRDGLLNDVFTLDLDAEQPTWCEVAGAAPPLPRSW

KfZTL        HSSCTVDGTYLVVFGGCTDTGRLLSDTFLLDLTAQKPVWREIVGGFKPPS
AtFKF1       HSSCTIEGSKLVVSGGCTDAGVLLSDTFLLDLTTDKPTWKEIPTSWAPPS
OsFKF1       HSSCTLDGSKLVVSGGCTESGVLLSDTFLLDLTKEKPAWKEIPTSWSPPS
AaZTL        HSSCTLDGTKLVVSGGSADSG-LLSDTFILDVAKEEPIWQEIPVSWSPPA
AtLKP2       HSSCTLDGTKLIVSGGCADSGALLSDTFLLDLSMDIPAWREIPVPWTPPS
SmZTL        HSSCTLDGTQLVVYGGCADSGVLLSDTYMLDISKEKPMWREIPVAWTPPS
```

```
OsZTL1      HSSCTLDGTKLVVSGGCADSGVLLSDTYLLDVTMERPVWREIPASWTPPC
OsZTL2      HSSCTLDGTKLVVSGGCADSGVLLSDTYLLDVTMDKPVWREVPASWTPPS
PaZTL       HSSCTLDGTKLVVSGGCADSGVLLSDTFLLDLTMEKPIWKEIPVSWTPPS
AtZTL       HSSCTLDGTKLIVSGGCADSGVLLSDTFLLDLSIEKPVWREIPAAWTPPS
MpFKF1      HSSCTLDGTKLVVSGGCADSGVLLSDTFLLDLTMEKPMWREIHVSWSPPS
TlZTL       HSSCTVDATKLVVSGGCADSGVLLSDIFLLDLTMKKPKWREFPVSWSPPS

KfZTL       RLGHSLSVLEGSKVLMFGGLAQSGPLRLRSSDVFTIDVGIQEPNWNYLTG
AtFKF1      RLGHSLSVFGRTKILMFGGLANSGHLKLRSGEAYTIDLEDEEPRWRELEC
OsFKF1      RLGHTLSVFGKTKLFMFGGLAKSGSLRLRSCDAYTMDAGEDSPQWRQLAT
AaZTL       RLGHTLTVYGDKKILMFGGLAKSGPLSLRSSDVFTIDLSEEEPKWKYVTG
AtLKP2      RLGHTLTVYGDRKILMFGGLAKNGTLRFRSNDVYTMDLSEDEPSWRPVIG
SmZTL       RLGHSLSAYGGRKILLFGGLAKSGPLRFRSSDAFTIDLGEEEPTWKYVTG
OsZTL1      RLGHSLSVYDGRKILMFGGLAKSGPLRLRSNDVFTLDLSENKPCWRCITG
OsZTL2      RLGHSMSVYGGRKILMFGGLAKSGPLRLRSSDVFTMDLSEEEPCWRCLTG
PaZTL       RLGHSLTVYGGRKILMFGGLAKSGPLRLRSSDVYTIDLSEEEPKWRYLTG
AtZTL       RLGHTLSVYGGRKILMFGGLAKSGPLKFRSSDVFTMDLSEEEPCWRCVTG
MpFKF1      RLGHTLCVYQGWKVLMFGGLAKSGPLRLRSSDVFTIDLSEEQPKWKYVTG
TlZTL       RLGHTLSVYGGCKILMFGGLAKSGPLRLRSSDVFTIDLSEDVPKWKYVTG

KfZTL       SMLPGGAPATGPSPPPRLDHVAATLPGGRVLIFGGSVAGQHSPIQLYVLD
AtFKF1      SAFPG-----VVVPPPRLDHVAVSMPCGRVIIFGGSIAGLHSPSQLFLID
OsFKF1      TGFP------SIGPPPRLDHVAVSLPCGRIIIFGGSIAGLHSPSQLFLLD
AaZTL       SSLPGGATPADTPPPPRLDHVAVSLTGGRVLIFGGSIAGLHSASQLYLLD
AtLKP2      YGSSLPM----AAPPPRLDHVAISLPGGRILIFGGSVAGLDSASQLYLLD
SmZTL       STLPGGANIGGTTPPPRLDHVAVTLPGGRILIFGGSIAGLHSASQIYLLD
OsZTL1      SGMPGASNPAGVGPPPRLDHVAVSLPGGRILIFGGSVAGLHSASKLYLLD
OsZTL2      SGMPGAGNPAGAGPPPRLDHVAVSLPGGRVLIFGGSVAGLHSASQLYLLD
PaZTL       SGMPGAGNPGGKAPPPRLDHVAVSLPGGRVLIFGGSVAGLHSASQLYLLD
AtZTL       SGMPGAGNPGGVAPPPRLDHVAVNLPGGRILIFGGSVAGLHSASQLYLLD
MpFKF1      STLPGGAAPAGTPPPPRLDHVAVSLPGGRILIFGGSIAGLHSPAQLFVLD
TlZTL       STLPGGATPAGTPPPPRLDHVAVSLPGGRVLIFGGSIAGLHSASQLYLLD

KfZTL       PKEGRPSWRMLNAPGLLPQYAWGHSTCVVGGTRAVVLGGFEGEERLLNEL
AtFKF1      PAEEKPSWRILNVPGKPPKLAWGHSTCVVGGTRVLVLGGHTGEEWILNEL
OsFKF1      PAEEKPTWRILNVPGQPPKFAWGHSTCVVGGTRVLVLGGHTGEEWILNEL
AaZTL       PAEEKPTWRMLDVPGDQPKFAWGHSTCVVGGIRAVVMGGHTGEEWILNEL
AtLKP2      PNEEKPAWRILNVQGGPPRFAWGHTTCVVGGTRLVVLGGQTGEEWMLNEA
SmZTL       PSEEKPTWRMLNVPGQKPKFAWGHSTCFVGGTRAVVLGGHTGEDWILNEL
OsZTL1      PTEEKPTWRILNVPGRPPRFAWGHSTCVVGGTKAIVLGGQTGEEWTLTEL
OsZTL2      PTEEKPTWRILNVPGRPPRFAWGHSTCVVGGTKAIVLGGQTGEEWMLTEI
PaZTL       PTEEKPTWRILNVPGQQPRFAWGHSTCVVGGTRALVLGGHPGEEWILNEL
AtZTL       PTEDKPTWRILNIPGRPPRFAWGHGTCVVGGTRAIVLGGQTGEEWMLSEL
MpFKF1      PKEEQSTWRVLNVPGQPPKFAWGHSTCVVGGTRAVVLGGHTGEEWILNEL
TlZTL       PIEEKPTWRMLNVPGQQPKFAWGHSTCVVGGTRAVVLGGHTGEEWILNEL

KfZTL       HELSLLDVP
```

```
AtFKF1      HELCLASRQ
OsFKF1      HELCLASRP
AaZTL       HELSLASKM
AtLKP2      HELLLATST
SmZTL       HELSLSSTS
OsZTL1      HELSLMFPT
OsZTL2      HELSLASST
PaZTL       HELSLASKH
AtZTL       HELSLASYL
MpFKF1      HELSITHKS
TlZTL       HELSLASKV

;
end;
```

Fig. S2 Inferred phylogeny of homologs to the ELF3 gene family. The tree was constructed using MrBayes and PhyML on an amino acid alignment of proteins retrieved from *Arabidopsis thaliana* (At), *Oryza sativa* (Os), *Picea abies* (Pa), *Selaginella moellendorffii* (Sm), *Physcomitrella patens* (Pp), *Marchantia polymorpha* (Mp), *Anthoceros agrestis* (Aa), and *Klebsormidium flaccidum* (Kf). The Bayesian tree is shown with posterior probabilities (above) and bootstrap proportions from PhyML analysis (below) for each node. Nodes with conflicting support from the two methods were collapsed. Branch length is relative to the thickness of individual branches: the shortest branches have a straight line and the longest are increasingly triangular.

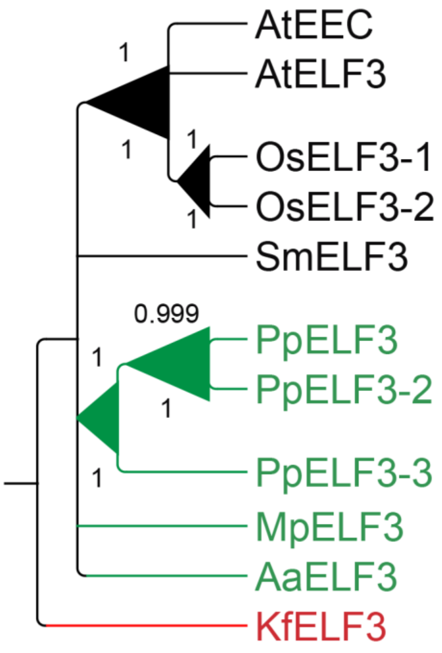

Fig. S3 Inferred phylogeny of homologs to the ELF4 gene family. The tree was constructed using MrBayes and PhyML on an amino acid alignment of proteins retrieved from *Arabidopsis thaliana* (At), *Oryza sativa* (Os), *Picea abies* (Pa), *Selaginella moellendorffii* (Sm), *Physcomitrella patens* (Pp), *Marchantia polymorpha* (Mp), *Anthoceros agrestis* (Aa), and *Klebsormidium flaccidum* (Kf). The Bayesian tree is shown with posterior probabilities (above) and bootstrap proportions from PhyML analysis (below) for each node. Nodes with conflicting support from the two methods were collapsed. Branch length is relative to the thickness of individual branches: the shortest branches have a straight line and the longest are increasingly triangular.

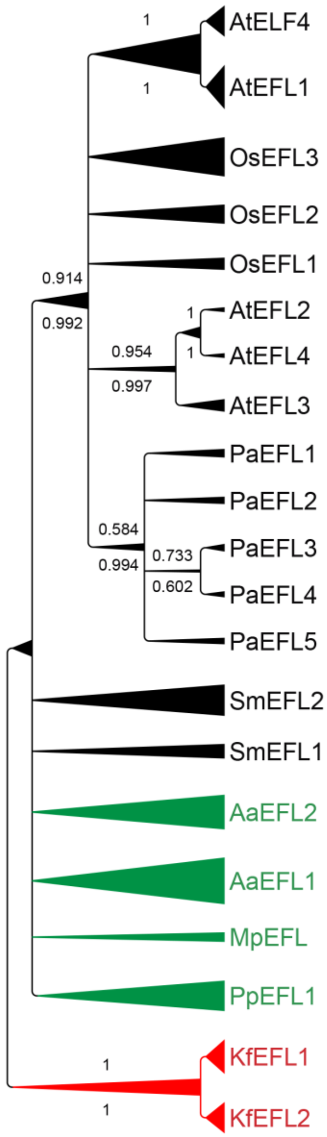

Fig. S4 Inferred phylogeny of homologs to the LUX gene family. The tree was constructed using MrBayes and PhyML on an amino acid alignment of proteins retrieved from *Arabidopsis thaliana* (At), *Oryza sativa* (Os), *Picea abies* (Pa), *Selaginella moellendorffii* (Sm), *Physcomitrella patens* (Pp), *Marchantia polymorpha* (Mp), *Anthoceros agrestis* (Aa), and *Klebsormidium flaccidum* (Kf). The Bayesian tree is shown with posterior probabilities (above) and bootstrap proportions from PhyML analysis (below) for each node. Nodes with conflicting support from the two methods were collapsed. Branch length is relative to the thickness of individual branches: the shortest branches have a straight line and the longest are increasingly triangular. The SHAQKYF-like motif in LUX homologs is SHLQKYR.

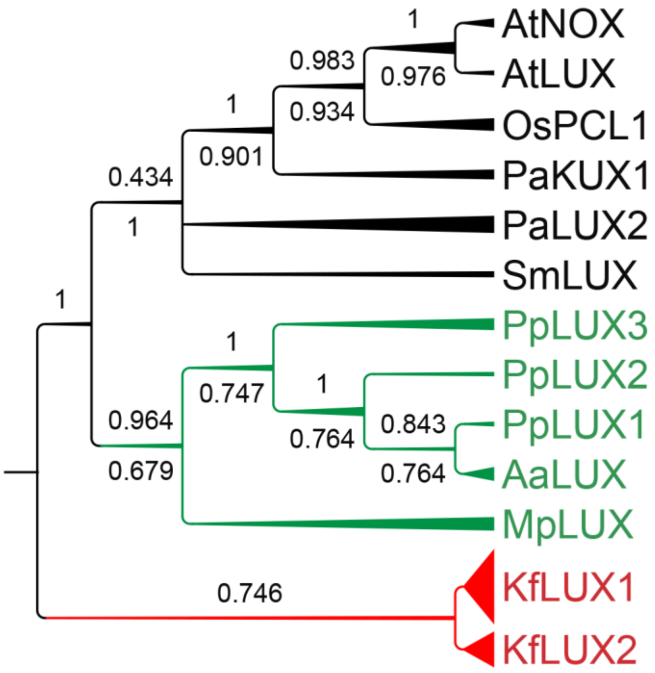

Fig. S5 Inferred phylogeny of homologs to the GI gene family. The tree was constructed using MrBayes and PhyML on an amino acid alignment of proteins retrieved from *Arabidopsis thaliana* (At), *Oryza sativa* (Os), *Picea abies* (Pa), *Selaginella moellendorffii* (Sm), *Marchantia polymorpha* (Mp), *Anthoceros agrestis* (Aa), *Cylindrocystis cushleckae* (Cc), *Coleochaete irregularis* (Ci) and *Takakia lepidozioides* (Tl). The Bayesian tree is shown with posterior probabilities (above) and bootstrap proportions from PhyML analysis (below) for each node. Nodes with conflicting support from the two methods were collapsed. Branch length is relative to the thickness of individual branches: the shortest branches have a straight line and the longest are increasingly triangular. † indicates gene loss in Pp (as well as all other mosses except Takakia).

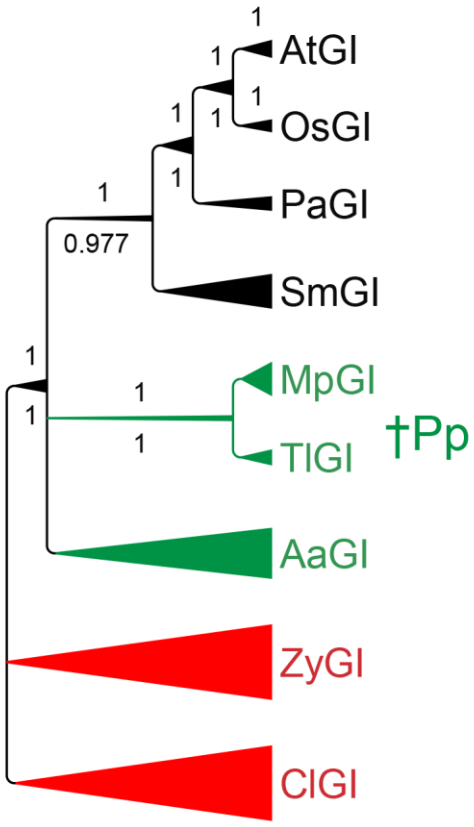

Fig. S6 Inferred phylogeny of homologs to the ZTL gene family. The tree was constructed using MrBayes on an amino acid alignment of proteins retrieved from *Arabidopsis thaliana* (At), *Oryza sativa* (Os), *Picea abies* (Pa), *Selaginella moellendorffii* (Sm), *Takakia lepidozioides* (Ti), *Marchantia polymorpha* (Mp), *Anthoceros agrestis* (Aa), and *Klebsormidium flaccidum* (Kf). The Bayesian tree is shown with posterior probabilities (above) and bootstrap proportions from PhyML analysis (below) for each node. Nodes with conflicting support from the two methods were collapsed. Branch length is relative to the thickness of individual branches: the shortest branches have a straight line and the longest are increasingly triangular. † indicates gene loss in *Physcomitrella patens* (Pp; as well as all other mosses except Takakia).

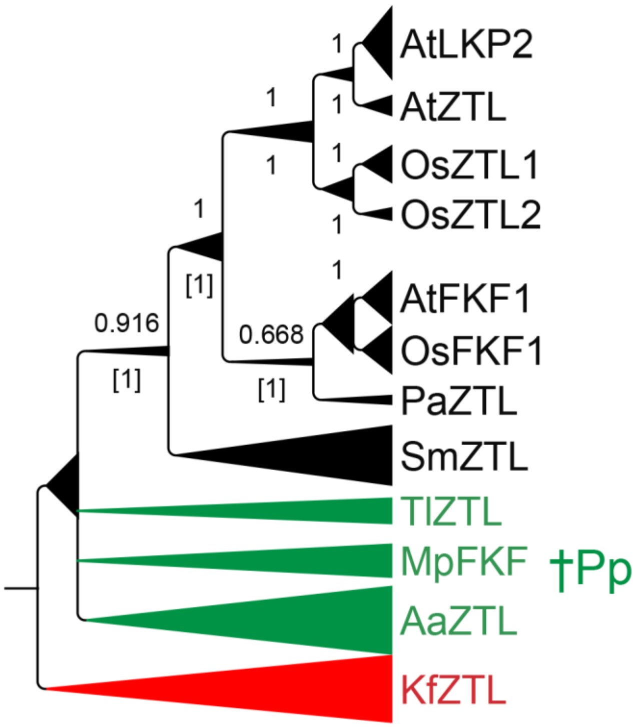

Fig. S7 Temporal expression patterns of putative circadian clock genes in *M. polymorpha* (Mp) under ND, LL, and DD conditions. Plants were sampled every fourth hour during 2 d. Quantitative RT-PCR expression values with standard errors are based on two biological replicates and were normalized using three reference genes (see the Materials and Methods section for details).

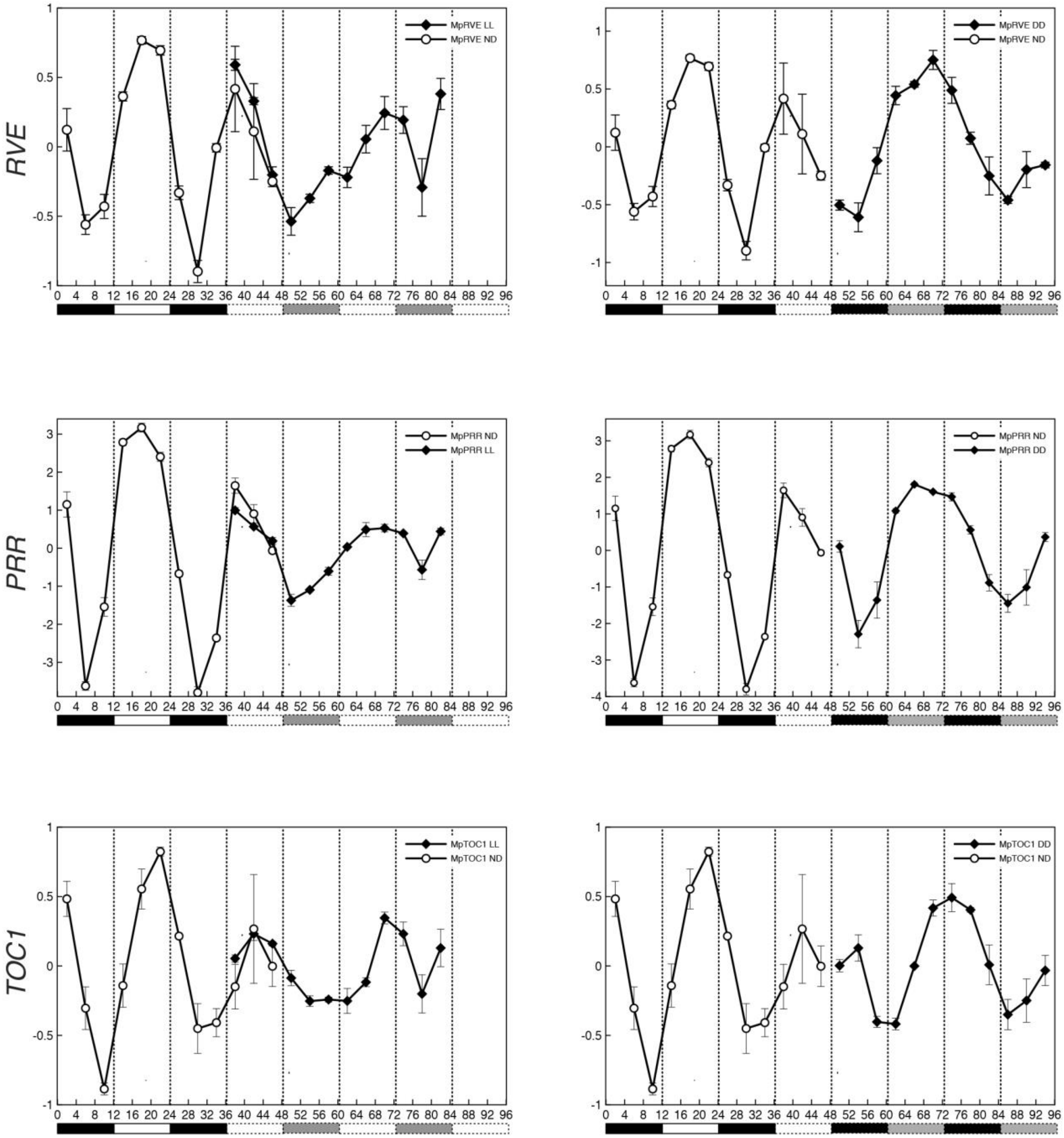

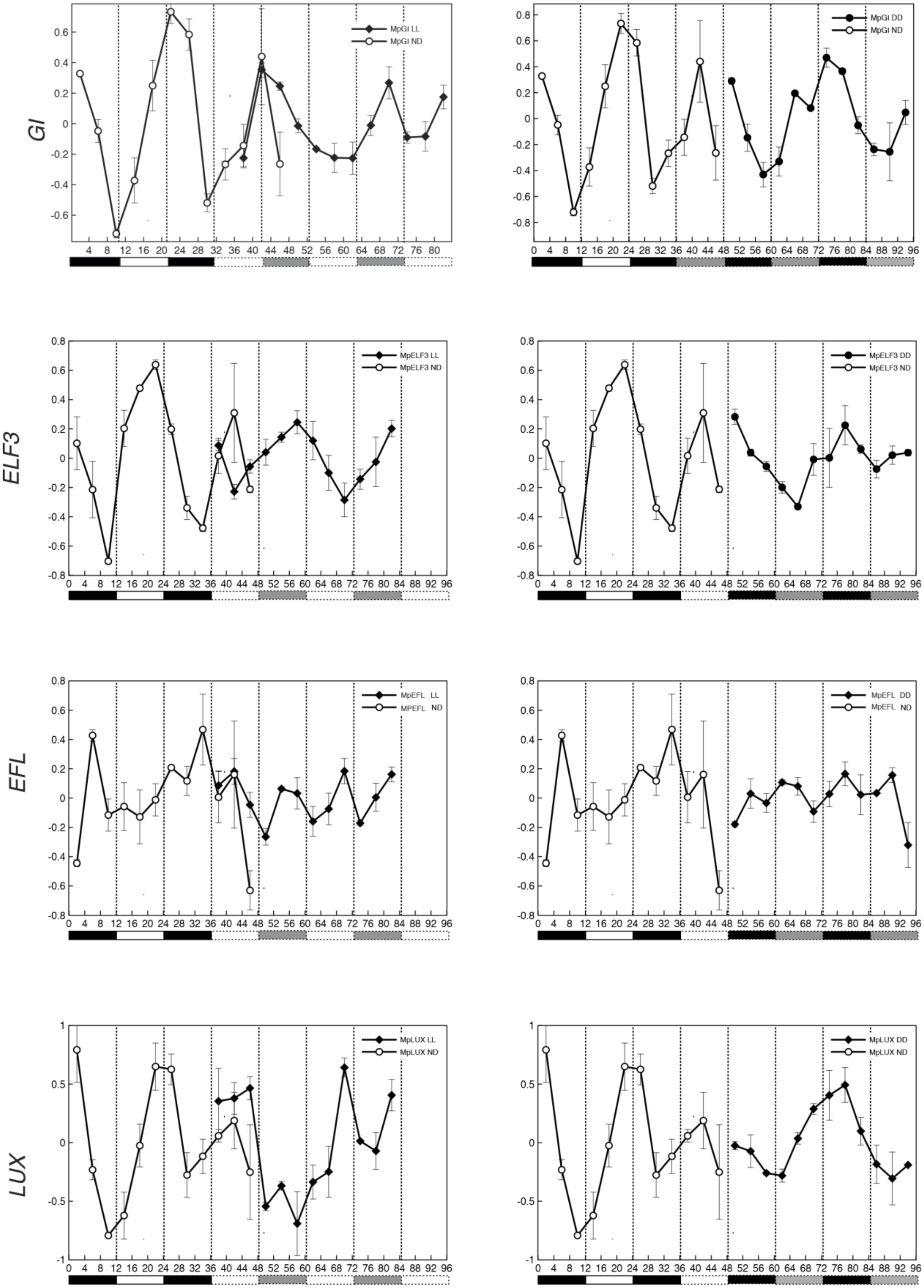
GI
MpGI LL
MpGI ND
MpGI DD
MpGI ND
ELF3
MpELF3 LL
MpELF3 ND
MpELF3 DD
MpELF3 ND
EFL
MpEFL LL
MPEFL ND
MpEFL DD
MpEFL ND
LUX
MpLUX LL
MpLUX ND
MpLUX DD
MpLUX ND

Fig. S8 Generation of Mp*RVE* knockout mutant. The exon-intron structure of Mp*RVE* and the construct used for Mp*RVE* transformation are shown in (a). Primer sets used in (b) are illustrated as arrows with numbers. The result of genomic PCR of Mp*RVE* knock-out line and WT are shown in (b). Numbers refer to primer sets as shown in (a).

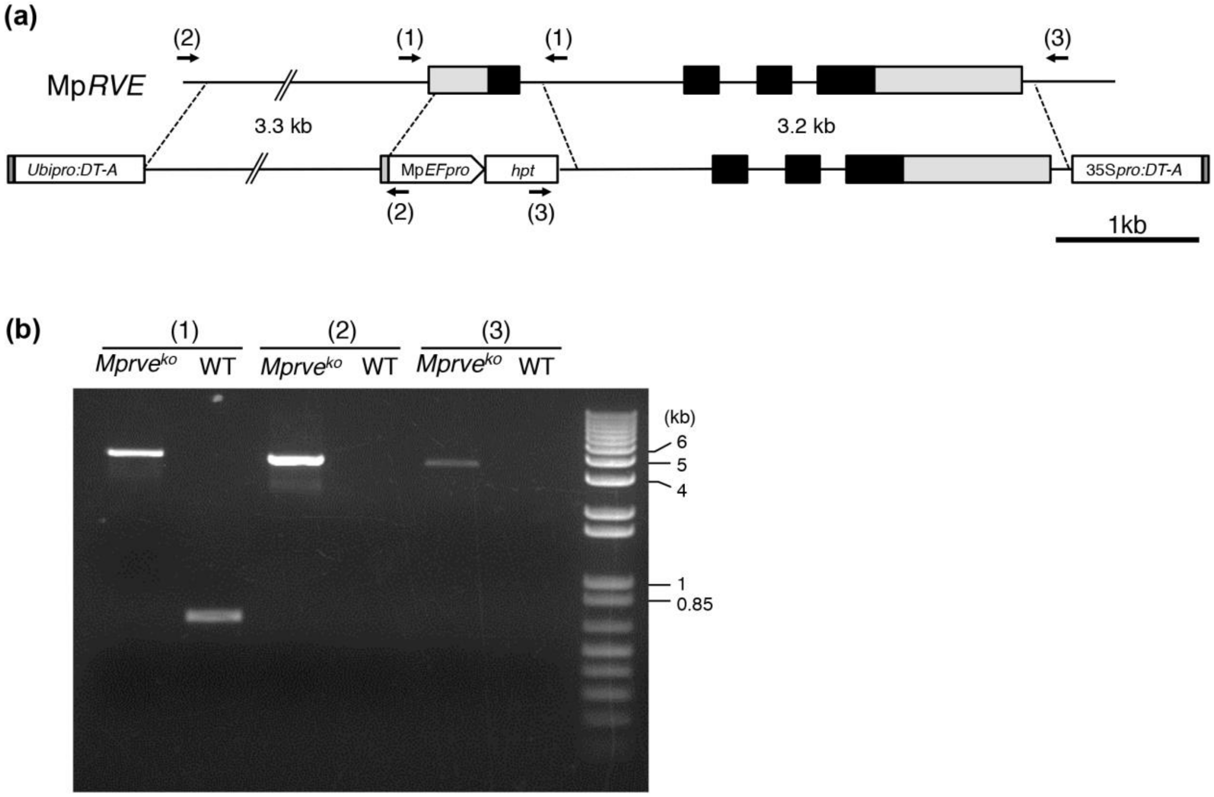

Fig. S9 Generation of Mp*PRR* knockout mutant. The exon-intron structure of Mp*PRR* and the construct used for Mp*PRR* transformation are shown in (a). Primer sets used in (b) are illustrated as arrows with numbers. The result of genomic PCR of Mp*PRR* knock-out line and WT are shown in (b). Numbers refer to primer sets as shown in (a).

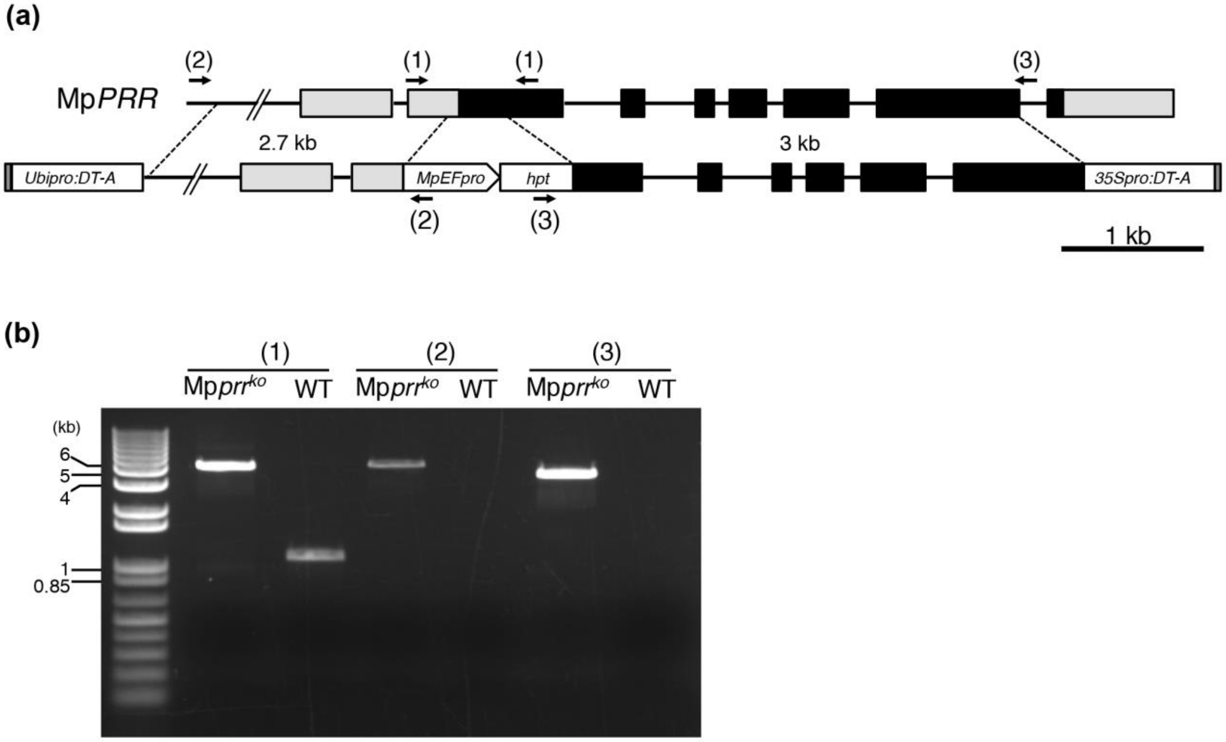

Fig. S10 Generation of Mp*TOC1* knockout mutants. The exon-intron structure of Mp*TOC1* and the construct used for Mp*TOC1* transformation are shown in (a). Primer sets used in (b) are illustrated as arrows with numbers. The result of genomic PCR of three Mp*TOC1* knock-out lines and WT are shown in (b). Numbers refer to primer sets as shown in (a).

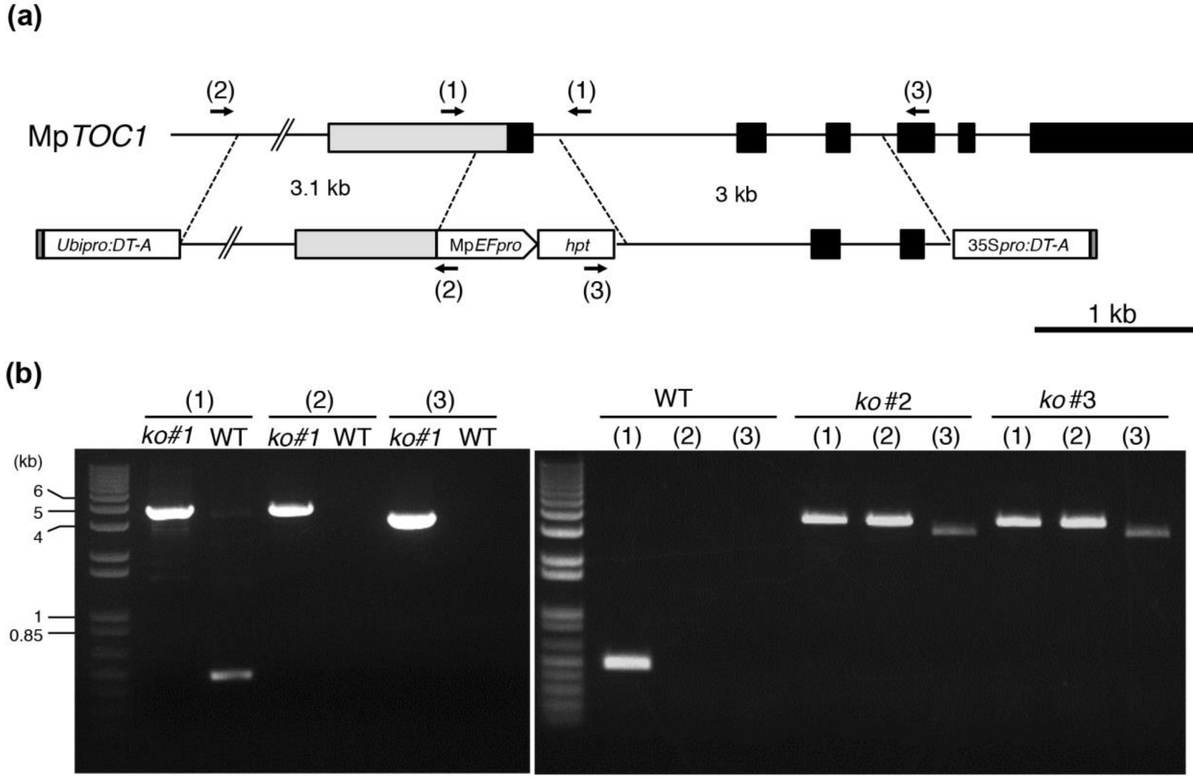

Fig. S11 Temporal expression pattern of Mp*PRR* (a) and *35S:LUC* (b) under ND and LL conditions. Quantitative RT-PCR expression values with standard errors are based on 3 biological replicates and were normalized using Mp*EF1*.

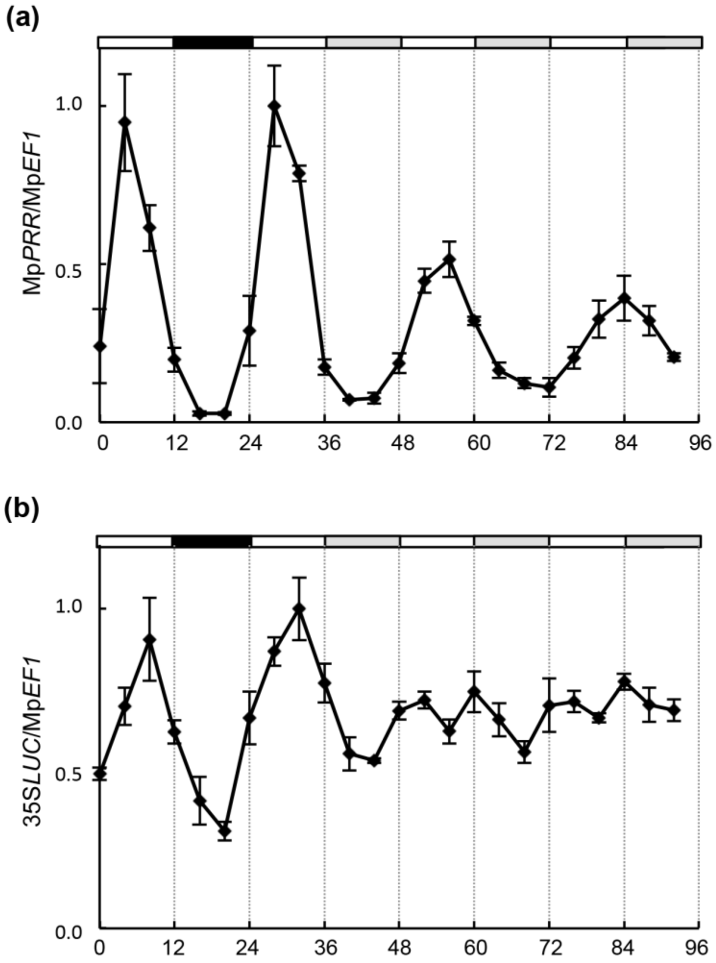

Fig. S12 Temporal expression pattern of Mp*PRR* in WT, Mp*rve*$_{ko}$, Mp*toc1*$_{ko}$ and restored lines of Mp*rve*^{ko}. Mp*PRR* expression in WT, Mp*rve*^{ko} and a restored line are shown in (a). Mp*PRR* expression under ND conditions (b) and LL conditions (c) in WT and two additional Mp*toc1*^{ko} lines. Quantitative RT-PCR expression values with standard errors are based on 3 biological replicates and were normalized using Mp*EF1*.

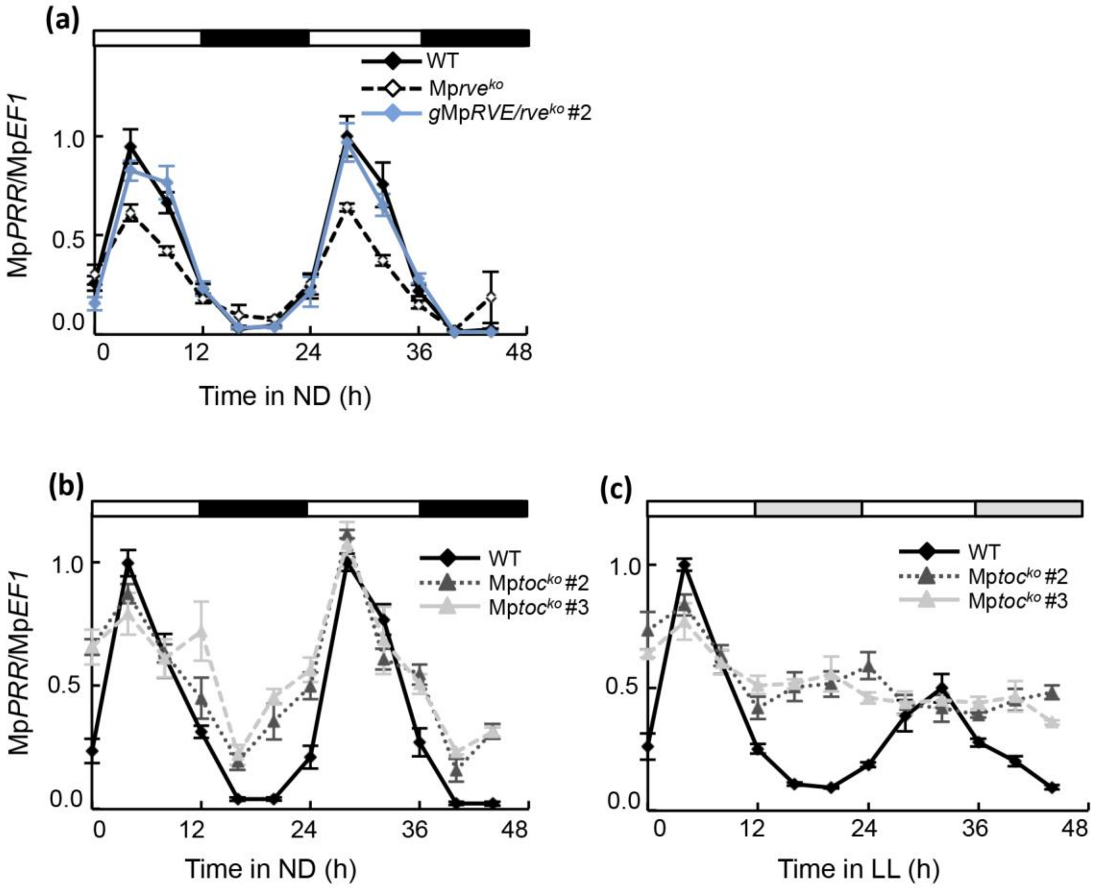

Fig. S13 *pro:LUC* bioluminescence for Mp*ELF3*, Mp*GI*, Mp*LUX* and Mp*RVE*. Plants were entrained in 12 h light, 12 h dark photoperiod and transferred to T=12 photocycles. Light intensity was set to 5 µmol m^{-2} s^{-1}. Averages from three replicates of one transformant per gene are shown. Expression patterns were readily adjusted to a T=12 photocycle without frequency demultiplication.

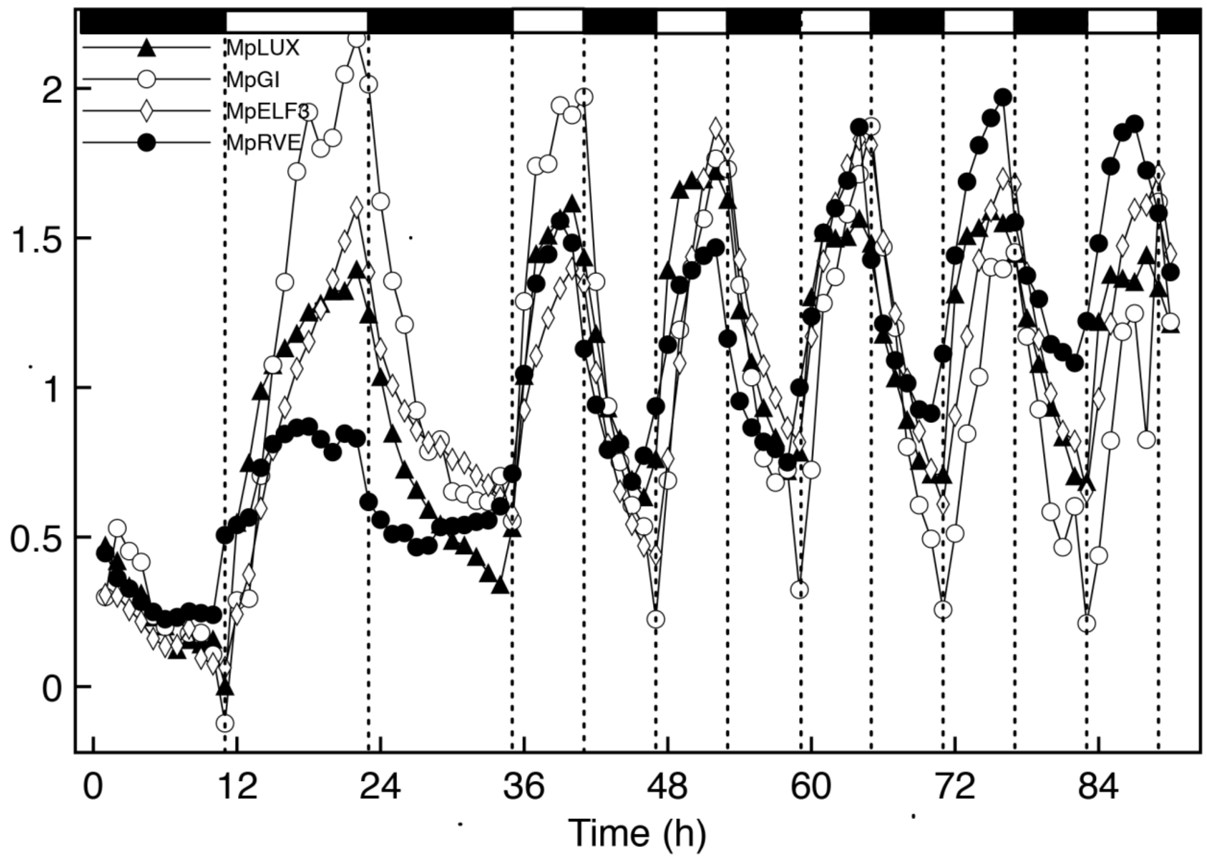

Fig. S14 Luciferase imaging in transgenic *M. polymorpha* plants expressing luciferase under the control of *M. polymorpha* promoters. (a–c) MpRVE_{pro}:*LUC*. (d–f) Mp$ELF3_{pro}$:*LUC*. (g–i) MpGI_{pro}:*LUC*. (j–l) MpLUX_{pro}:*LUC*. (a, d, g, j) 4-d-old gemmaling showing expression at the apical notches. (b, e, h, k) 7-d-old gemmaling with expression at the recently split apical notches. (c, f, j, l) 4-wk-old thallus showing strong expression in apical regions and young gemmae cups. Bioluminescense is pseudocolored in green.

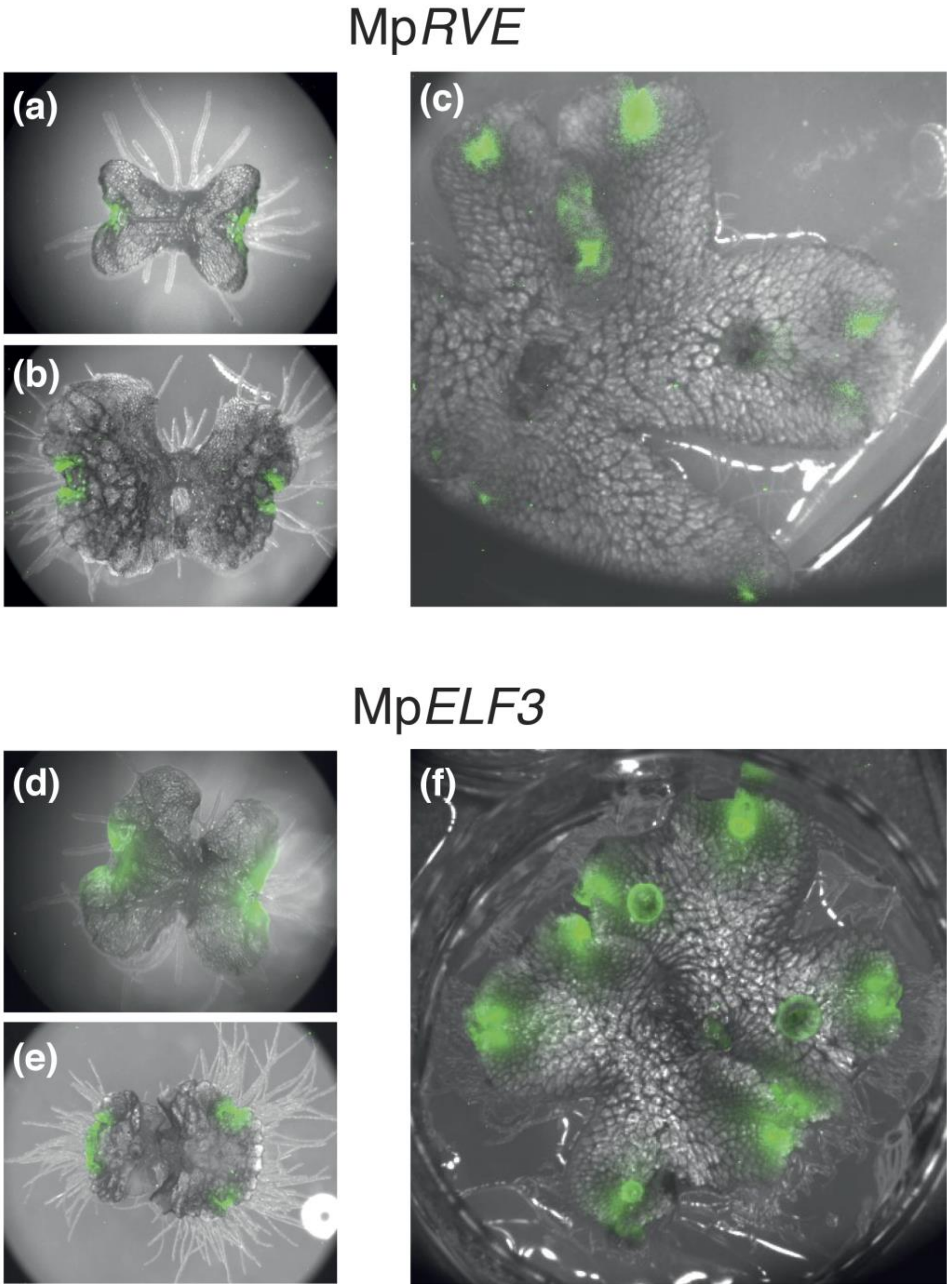

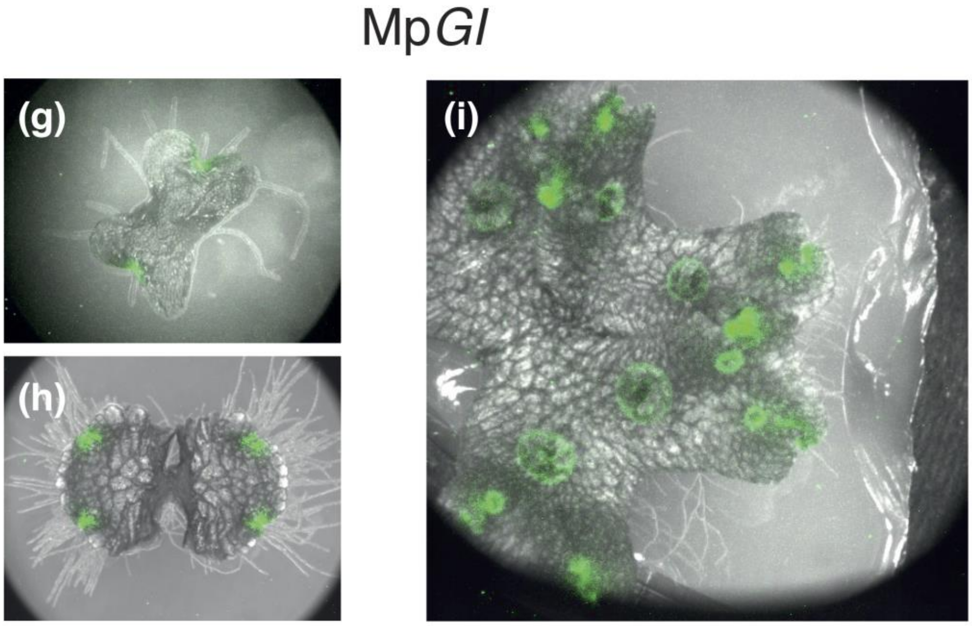
MpGI
(g)
(h)
(i)

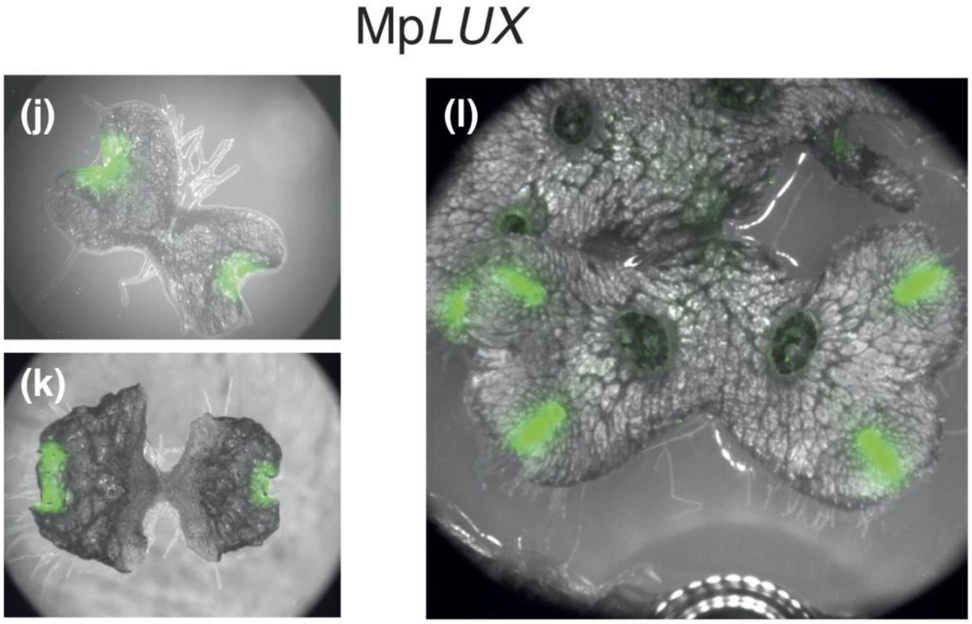
MpLUX
(j)
(k)
(l)

Fig. S15 Mp*PRR*$_{pro}$*:GUS* expression in mature thallus. Mp*PRR*$_{pro}$*:GUS#7* stained overnight (a, c) or for 4 h (b). Dorsal side (a), and ventral side (b) of mature thallus. Close-up of dorsal side showing air chambers with expression in chlorenchyma cells (c). Arrowheads points at strongly stained meristematic regions.

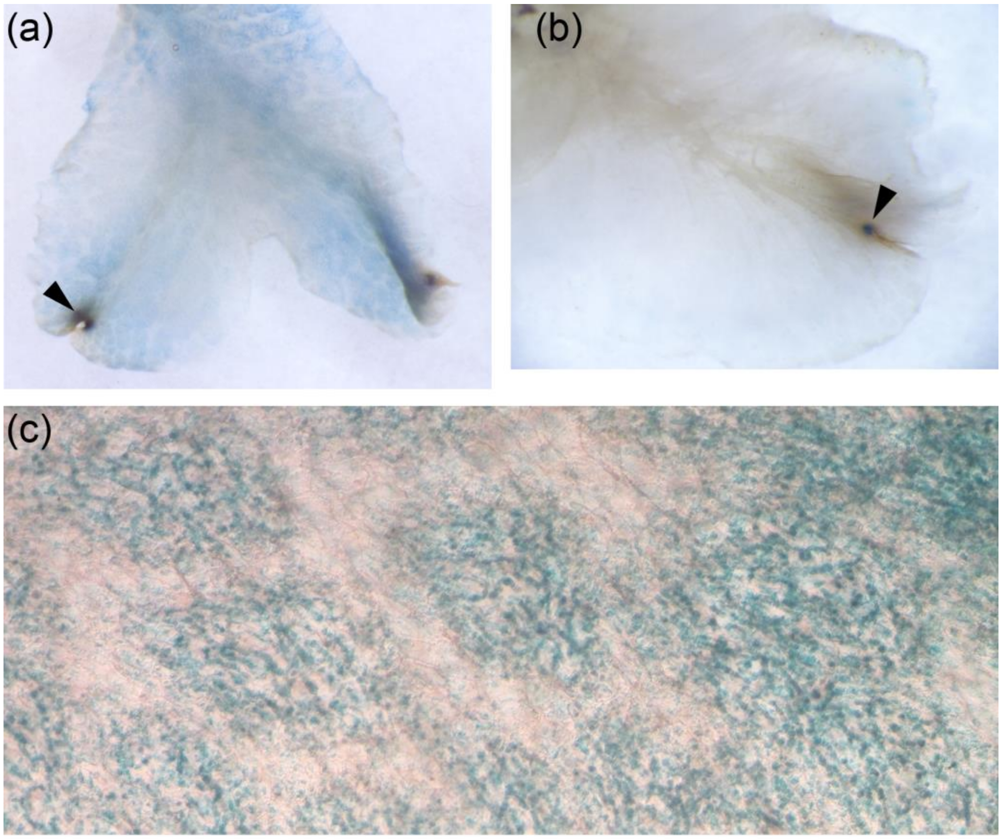

Fig. S16 Temporal expression patterns of putative circadian clock genes in *A. agrestis* (Aa) under ND, LL, and DD conditions. Plants were sampled every fourth hour during 2 d. Quantitative RT-PCR expression values with standard errors are based on two biological replicates and were normalized using two reference genes (see the Materials and Methods section for details).

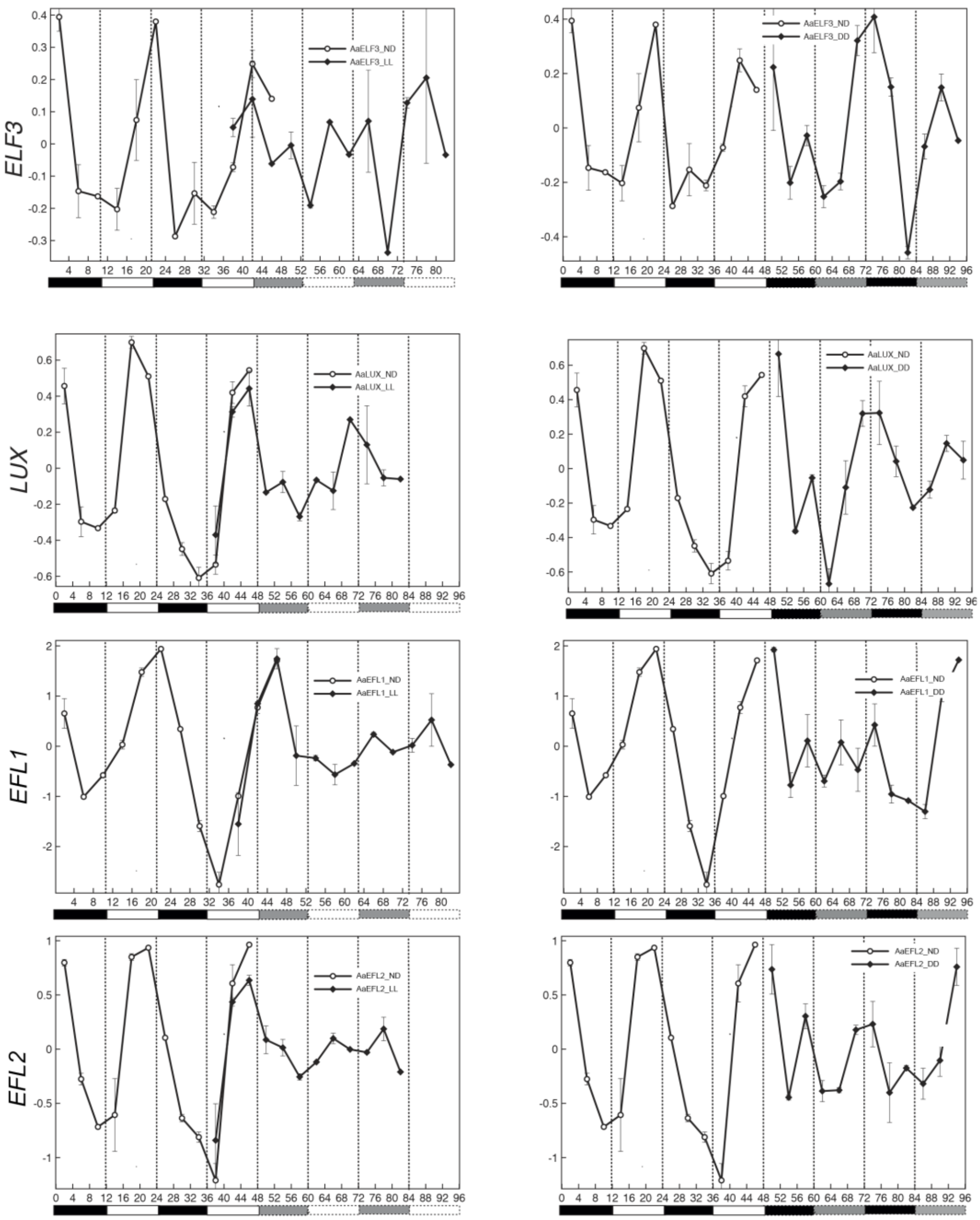

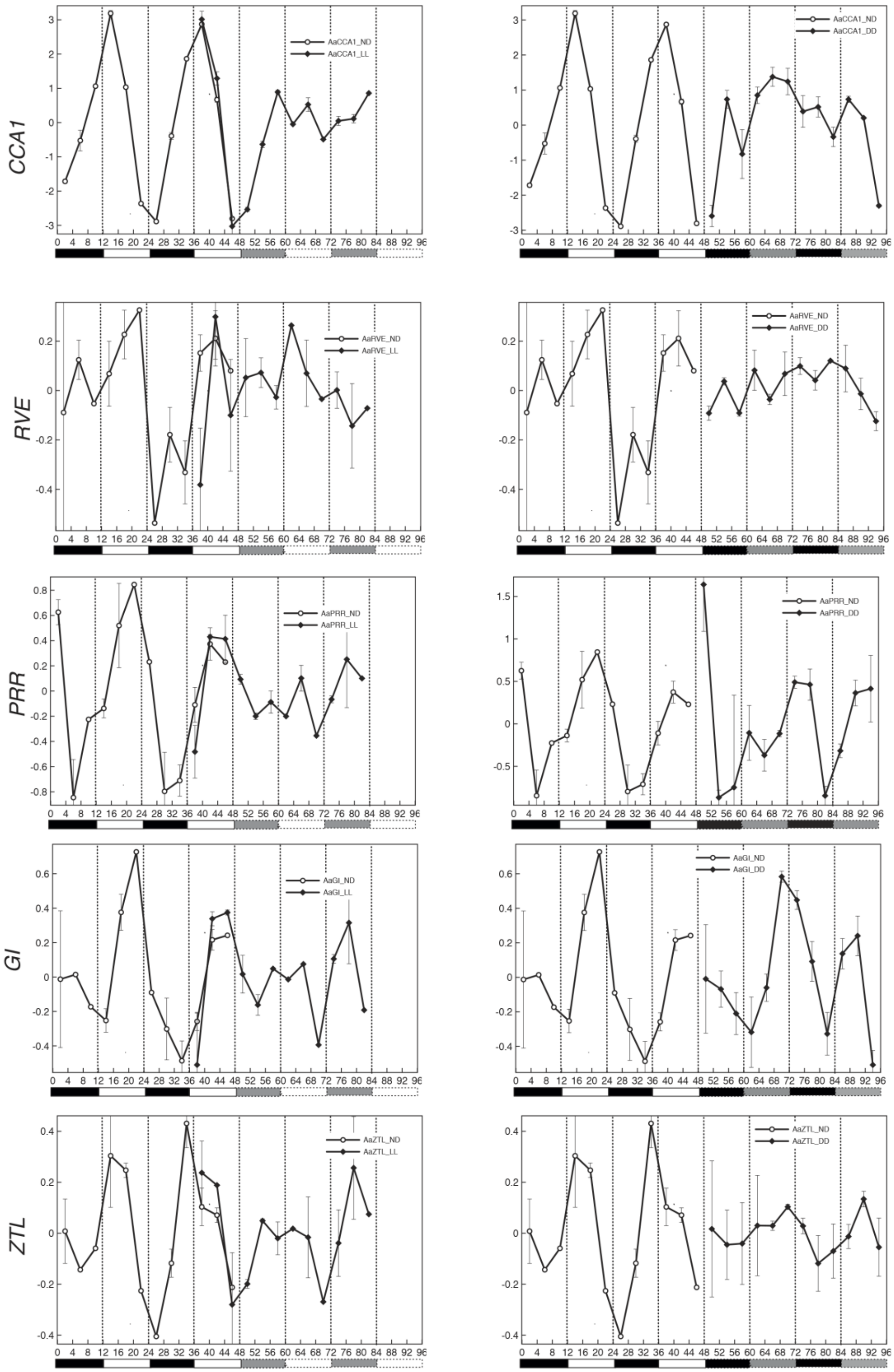
CCA1
AaCCA1_ND
AaCCA1_LL
AaCCA1_ND
AaCCA1_DD
RVE
AaRVE_ND
AaRVE_LL
AaRVE_ND
AaRVE_DD
PRR
AaPRR_ND
AaPRR_LL
AaPRR_ND
AaPRR_DD
GI
AaGI_ND
AaGI_LL
AaGI_ND
AaGI_DD
ZTL
AaZTL_ND
AaZTL_LL
AaZTL_ND
AaZTL_DD

Table S1 Gene names, family/sub-clade, gene ID or accession number.

**Species**	**Gene name**	**Gene family/Clade**	**ID/Accession**
Chlamydomonas reinhardtii	Data from Phytozome v11.0 (https://phytozome.jgi.doe.gov)		
	CrLHY (ROC40)	RVE	Cre06.g275350
	CrPRR-1	PRR	Cre02.g094150
	CrPRR-2	PRR	Cre16.g676421
	CrLUX-1 (ROC75)	LUX[?]	Cre02.g083750
	CrLUX-2 (ROC15)	LUX[?]	Cre09.g410450
	CrEFL	ELF4[?]	
Ostreococcus tauri	Data from JGI (http://genome.jgi.doe.gov)		
	OtCCA1	RVE	4802
	OtTOC1	PRR	24394
Klebsormibium flaccidum	Data from Klebsormidium flaccidum genome V1.0 (http://www.plantmorphogenesis.bio.titech.ac.jp/ ~algae_genome_project/klebsormidium/)		
	KfCCA1	RVE/CCA1-LHY-clade	kfl00255_0190
	KfRVE	RVE/LCL clade	kfl00237_0160
	KfTOC1	PRR/TOC1 clade	kfl00059_0230
	KfPRR	PRR/PRR clade	kfl00165_0030
	KfZTL	ZTL	kfl00059_0220
	KfELF3	ELF3	kfl00240_0090
	KfEFL-1	ELF4	kfl00065_0150
	KfEFL-2	ELF4	kfl00184_0080
	KfLUX-1	LUX	kfl00118_0040
	KfLUX-2	LUX	kfl00537_0090
Anthoceros agrestis	Data from the oneKP database (www.onekp.com)		
	AaCCA1	RVE/CCA1-LHY-clade	
	AaRVE	RVE/LCL-clade	
	AaPRR	PRR/PRR clade	
	AaELF3	ELF3	
	AaEFL-1	ELF4	
	AaEFL-2	ELF4	
	AaLUX	LUX	
	AaGI	GI	
	AaZTL	ZTL	
Marchantia polymorpha	Data from Phytozome v11.0 (https://phytozome.jgi.doe.gov)		
	MpRVE[1]	RVE/CCA1-LHY-clade	Mapoly0042s0058.1
	MpTOC1[2]	PRR/TOC1 clade	Mapoly0085s0081.1

	MpPRR[2]	PRR/PRR clade	Mapoly0122s0007.1
	MpELF3	ELF3	Mapoly0014s0139.1
	MpEFL	ELF4	Mapoly0033s0009.1
	MpLUX[3]	LUX	Mapoly0101s0068.1
	MpGI	GI	Mapoly0019s0145.1
	MpFKF	ZTL	Mapoly0004s0235.1
Physcomitrella patens	Data from Phytozome v11.0 (https://phytozome.jgi.doe.gov)		
	PpCCA1a	RVE/CCA1-LHY-clade	BAI39991.1
	PpCCA1b	RVE/CCA1-LHY-clade	BAI39992.1
	PpRVE1	RVE/LCL clade	Pp1s198_135
	PpRVE2	RVE/LCL clade	Pp1s160_6
	PpRVE3	RVE/LCL clade	Pp1s46_272
	PpPRR1	PRR/PRR clade	XP_001784613
	PpPRR2	PRR/PRR clade	XP_001766102
	PpPRR3	PRR/PRR clade	XP_001784616
	PpPRR4	PRR/PRR clade	XP_001766105
	PpELF3	ELF3	Pp3c1_12790
	PpELF3-3	ELF3	Pp3c11_14750
	PpELF3-2	ELF3	Pp3c7_10610
	PpEFL	ELF4	Pp1s180_31
	PpLUX1	LUX	Pp3c9_14170
	PpLUX2	LUX	Pp3c21_6770
	PpLUX3	LUX	Pp3c15_13960
Selaginella moellendorffii	Data from Phytozome v11.0 (https://phytozome.jgi.doe.gov)		
	SmRVE	RVE/LCL clade	78045
	SmTOC1	PRR/TOC1 clade	438647
	SmPRR7a	PRR/PRR clade	XP_002972852†
	SmPRR7b	PRR/PRR clade	XP_002979868†
	SmELF3	ELF3	415241
	SmEFL-1	ELF4	118674
	SmEFL-2	ELF4	91083
	SmZTL	ZTL	174189
	SmLUX	LUX	36646
	SmGI	GI	140066
Picea abies	Data from congenie.org		
	PaCCA1	RVE/CCA1-LHY-clade	MA_115536g0010
	PaCCA1-2	RVE/CCA1-LHY-clade	MA_11267g0020
	PaCCA1-3	RVE/CCA1-LHY-clade	MA_102199g0010
	PaPRR1	PRR/TOC1 clade	

	PaPRR3	PRR/PRR clade	
	PaPRR7	PRR/PRR clade	
	PaLUX	LUX	MA_3352g0010
	PaLUX2	LUX	MA_303578g0010
	PaZTL	ZTL	MA_70291g0010
	PaGI	GI	
	PaEFL-1	ELF4	MA_57007g0010
	PaEFL-2	ELF4	MA_99665g0010
	PaEFL-3	ELF4	MA_866497g0010
	PaEFL-4	ELF4	MA_4172g0010
	PaEFL-5	ELF4	MA_8565294g0010
Oryza sativa	Data from IRGSP-1.0 (http://rapdb.dna.affrc.go.jp)		
	OsCCA1	RVE/CCA1-LHY-clade	Os08t0157600
	OsRVE1	RVE/CCA1-LHY-clade	Os02t0685200
	OsRVE2	RVE/CCA1-LHY-clade	Os04g0583900
	OsRVE3	RVE/CCA1-LHY-clade	Os06t0728700
	OsRVE4	RVE/LCL clade	Os02t0680700
	OsRVE5	RVE/LCL clade	Os06t0105800
	OsRVE6	RVE/LCL clade	Os01t0156000
	OsPRR1	PRR/TOC1 clade	Os02g0618200
	OsPRR37	PRR/PRR clade	Os07g0695100
	OsPRR73	PRR/PRR clade	Os03g0284100
	OsPRR95	PRR/PRR clade	Os09g0532400
	OsPRR59	PRR/PRR clade	Os11g0157600
	OsZTL1	ZTL	Os11t0547000
	OsZTL2	ZTL	Os06t0694000
	OsFKF1	ZTL	Os11t0547000
	OsPCL1	LUX	Os01g0971800
	OsELF3-1	ELF3	Os06t0142600
	OsELF3-2	ELF3	Os01t0566100
	OsEFL-1	ELF4	OS11G0621500
	OsEFL-2	ELF4	OS03G0410300
	OsEFL-3	ELF4	OS08G0366200
	OsGI	GI	Os01g0182600
Arabidopsis thaliana	Data from TAIR 10 (www.arabidopsis.org)		
	AtCCA1	RVE/CCA1-LHY-clade	At2g46830
	AtLHY	RVE/CCA1-LHY-clade	At1g01060
	AtRVE1	RVE/CCA1-LHY-clade	At5g17300
	AtRVE2	RVE/CCA1-LHY-clade	At5g37260

	AtRVE3	RVE/LCL clade	At1g01520
	AtRVE4	RVE/LCL clade	At5g02840
	AtRVE5	RVE/LCL clade	At4g01280
	AtRVE6	RVE/LCL clade	At5g52660
	AtRVE7	RVE/CCA1-LHY-clade	At1g18330
	AtRVE7-like	RVE/CCA1-LHY-clade	At3g10113
	AtRVE8	RVE/LCL clade	At3g09600
	AtTOC1	PRR/TOC1 clade	At5g61380
	AtPRR3	PRR/PRR clade	Ag5g60100
	AtPRR5	PRR/PRR clade	Ag5g24470
	AtPRR7	PRR/PRR clade	Ag5g02810
	AtPRR9	PRR/PRR clade	Ag2g46790
	AtZTL	ZTL	At5g57360
	AtFKF1	ZTL	At1g68050
	AtLKP2	ZTL	At2g18915
	AtLUX	LUX	At3g46640
	AtBOA	LUX	At5g59570
	AtELF3	ELF3	At2g25930
	AtEEC	ELF3	At3g21320
	AtELF4	ELF4	At2g40080
	AtEFL-1	ELF4	At2g29950
	AtEFL-2	ELF4	At1g72630
	AtEFL-3	ELF4	At2g06255
	AtEFL-4	ELF4	At1g17455
	AtGI	GI	At1g22770

[?] Uncertain homology.
[1]The second most similar *M. polymorpha* gene from the BLAST searches, Mapoly0026s0070, was excluded from further analyses as it resembled At5g08520 and related genes with high similarity to *DIVARICATA* in reciprocal BLAST searches (Galego & Almeida, 2002).
[2]The third best *M. polymorpha* BLAST hit was Mapoly0101s0006. This protein contains a MYB domain not present in PRR family members and it also lack the C-terminal CCT domain. Reciprocal BLAST searches against the Arabidopsis genome gave RESPONSE REGULATOR1 (RR1) as the best hit, indicating Mapoly0101s0006 belongs to the RR family.
[3]As with MpTOC1/PRR the most similar protein to MpLUX in the *M. polymorpha* genome is the RR-family member Mapoly0101s0006.1.

Table S2 Oligonucleotides used in this study.

**Primer name**	**Sequence - 5′ to 3′**	**Comments**
AaCCA1 F	CGAAGCCATGCCCAGAAATT	qRT-PCR
AaCCA1 R	CGGTGGTGGGATGTCTATGT	qRT-PCR
AaELF3 F	AGGACGGCATTACTGACACA	qRT-PCR
AaELF3 R	GTGGATGACGGTGCTTTTCA	qRT-PCR
AaELF4_2011664 F	CCCAGTACCGGTGATGATGA	qRT-PCR
AaELF4_2011664 R	TCTGTTGCAGCGGTCAATAC	qRT-PCR
AaELF4_2045507 F	TCACTGATCACGGAGCTGAA	qRT-PCR
AaELF4_2045507 R	CCACACTCTGAAACGTGCTT	qRT-PCR
AaELF4_2048939 F	TGTCCGCTGTTTTCGAGAAG	qRT-PCR
AaELF4_2048939 R	GCCACACATCCAGTCTCCTT	qRT-PCR
AaGI F	GGGAGACGCTTCGGTTATTG	qRT-PCR
AaGI R	TCATCGTTCCCTCTGCTTGT	qRT-PCR
AaLUX F	ATGCCTGCTCACATGGGATT	qRT-PCR
AaLUX R	CCATAGGGTCCCATGCTCAA	qRT-PCR
AaPRR F	CTAACGTGGGAGGTTGAGGT	qRT-PCR
AaPRR R	ACCGATATCCACAGCACCAT	qRT-PCR
AaRVE F	CCGGCAAAAGAACATGGAGT	qRT-PCR
AaRVE R	CGGCCACAGGTGAAAGTAAC	qRT-PCR
AaTUB F	ACGCCTGAACATCTCCTGAA	qRT-PCR
AaTUB R	CGTGCAGAACAAGAACTCGT	qRT-PCR
AaZTL F	GGGTGGAGGTGGAGTATCTG	qRT-PCR
AaZTL R	GAGTGAAGAGGAGCCCAAGT	qRT-PCR
CPEP23_2	AGTACCTGACGACCACTAGCGAACC	Rev $MpRVE_{pro}$ 1.9kb
CPEP24	caccAGGTACCCGGGAGTTCAGTA[2]	Fwd $MpRVE_{pro}$ 1.9kb
CPEP29	caccTTGATGCTTCTCGCCTT[2]	Fwd $MpGI_{pro}$ 5.4kb
CPEP30	AGGGCCAATCTCCAAAGG	Rev $MpGI_{pro}$ 5.4kb
CPEP32	caccATGGTATGGGCAGTGGAGGC[2]	Fwd $MpLUX_{pro}$ 5.5kb
CPEP33	CGCTGCCATTCACTTCGAAGTGTG	Rev $MpLUX_{pro}$ 5.5kb
CPEP49	caccGGCTTGTTTCCTTGCTTG[2]	Fwd $MpELF4_{pro}$ 5.8kb
CPEP50	CGCTTCCAGACTCGCAAATCTCTACT	Rev $MpELF4_{pro}$ 5.8kb
**EFpro_R**	**CAACCTTTCTGCAGGCACATC**	**Genotyping**[19]
**gMpPRR_F**	**CACCAGCCTGCACATTCTTGGTCT**	**Fwd Compl. test**
**gMpPRR_R**	**TGAAGGGTAACAAGTGCAAACA**	**Rev Compl. test**
**gMpRVE_F**	**CACCATTGTTTGCAGCCCTTCTTT**	**Fwd Compl. test**
**gMpRVE_R**	**CGTGATTGGACGAGGAGATT**	**Rev Compl. Test**
**HPT_F**	**GTATAATGTATGCTATACGAAGTTATGTTT**	**Genotyping**[20]
ME292	caccTGTTTGATGCTGACAGTTTGGGAAA[2]	Fwd $MpPRR_{pro}$ 4.0kb
ME303	TAACGCACTAATCTGGCAAGCAACA	Rev $MpPRR_{pro}$ 4.0kb
ME367	CGAAAGCCCAAGAAGCTACC	Fwd MpAPT qRT-PCR[1]
ME368	GTACCCCCGGTTGCAATAAG	Rev MpAPT qRT-PCR[1]
ME369	AGGCATCTGGTATCCACGAG	Fwd MpACT qRT-PCR[1]
ME370	ACATGGTCGTTCCTCCAGAC	Rev MpACT qRT-PCR[1]
ME428	caccGGAAAACCATGGACTGACT[2]	Fwd $MpELF3_{pro}$ 2.1kb
ME429	GCCTCACTCTCACGACAACA	Rev $MpELF3_{pro}$ 2.1kb
MpELF3 F	ATCTTCCTCCCAGTCTTCTTCG	qRT-PCR
MpELF3 R	ACAGCAGATGATATTCGCAT	qRT-PCR
MpELF4 F	GGCAGCGCAATCTCAAGAAAA	qRT-PCR
MpELF4 R	AGGCTCATAATTCCCGCAGATT	qRT-PCR
MpGI F	TTGATCTGCGACCTCCTCATTT	qRT-PCR
MpGI R	CATGACCTTGGATGGAGAAGCT	qRT-PCR
MpLUX F	TGGGAGAGCATAGAGAATTCATG	qRT-PCR
MpLUX R	ATTTCTCATGACTCTCGCTCT	qRT-PCR
MpPRR F	CAGCAGCTCCTTTGAACAAACA	qRT-PCR
MpPRR R	GCCGTGAAGCAGGAAAGAGAAT	qRT-PCR

**MpPRR_GT3_F**	**AACACTAGTGGCGCGGCTGGGAAAGCTTCCTACCT[4]**	**Ko-plasm.**
**MpPRR_GT3_R**	**TTATCCCTAGGCGCGGCTGGAGAAGGCAAACTGAC[4]**	**Ko-plasm.**
**MpPRR_GT5_F**	**CTAAGGTAGCGATTAATCAACAATGAGCCATTCTATCTG[5]**	**Ko-plasm.**
**MpPRR_GT5_R**	**CCGGGCAAGCTTTTAATAAGGCGTGAACCTGTATCGT[5]**	**Ko-plasm.**
MpRVE F	AAACCTCGGCAAAATCAGGAGT	qRT-PCR
MpRVE R	GGCGAGGCAATTTTCAAAGCTG	qRT-PCR
**MpRVE_GT3_F**	**AACACTAGTGGCGCGCCTTAAAGTGGGGCAACTGA[6]**	**Ko-plasm.**
**MpRVE_GT3_R**	**TTATCCCTAGGCGCGCGGACCCCTGATAACTACGA[6]**	**Ko-plasm.**
**MpRVE_GT5_F**	**CTAAGGTAGCGATTAATCGTACGCCACCAAAGAATTT[7]**	**Ko-plasm.**
**MpRVE_GT5_R**	**CCGGGCAAGCTTTTAATTAAGCAGTCCGAGACCGAAT[7]**	**Ko-plasm.**
MpTOC1 F	CGAAGGAAGAACGACTGAAGCA	qRT-PCR
MpTOC1 R	TCTGAGACATTTGACGACGACA	qRT-PCR
**MpTOC1_GT3_F**	**AACACTAGTGGCGCGTCCTTTGCATTGTGATTTCG[8]**	**Ko-plasm.**
**MpTOC1_GT3_R**	**TTATCCCTAGGCGCGCTATGGACAGGTGGGTGCTT[8]**	**Ko-plasm.**
**MpTOC1_GT5_F**	**CAAAGGTAGCGATTAATCGTCAATGAAAGATGCGATG[9]**	**Ko-plasm.**
**MpTOC1_GT5_R**	**CCGGGCAAGCTTTTAATATGGAAATCTCCCTCGGAGT[9]**	**Ko-plasm.**
MpTUB[3] F	AGGGGCTGAACTCATAGACTCT	qRT-PCR
MpTUB[3] R	TATGAGGAGAGTACCCATGCCA	qRT-PCR
MpZTL F	TGTTCGAGTTCATAACAGGCTACA	qRT-PCR
MpZTL R	CATCGTTTAATCGTTGCAACAT	qRT-PCR
**prrko_F1**	**GTTTCGAAGAAGAGCTCCCA**	**Genotyping[13]**
**prrko_R1**	**GGATTCGGAAACCGATTTTT**	**Genotyping[13]**
**prrko_F2**	**AAAATGAAACGTGCTAATTTTGG**	**Genotyping[14]**
**prrko_R3**	**CGCAGACATCGAACACTGAT**	**Genotyping[15]**
**RP_LUC_F1**	**AATCCATCTTGCTCCAACACC**	**qRT-PCR**
**RP_LUC_R1**	**CCGTGCTCCAAAACAACAAC**	**qRT-PCR**
**RP_PRR_F1**	**CAGAAGACGATGATTCCACCAG**	**qRT-PCR**
**RP_PRR_R1**	**CATCTCCCAGGCTTGCATTC**	**qRT-PCR**
**rveko_F1**	**TGAGAATCCCTTATGCCCTG**	**Genotyping[10]**
**rveko_R1**	**TAGCATTCTTCAGGGGTTCG**	**Genotyping[10]**
**rveko_F2**	**TGATCAAATGCGACCAAATG**	**Genotyping[11]**
**rveko_R3**	**AAGGATTGCGCTCATGTTTT**	**Genotyping[12]**
**tocko_F1**	**GCTAGACACAGCCAACAACG**	**Genotyping[16]**
**tocko_R1**	**TTGCTAAGCCAAGTGGAGGT**	**Genotyping[16]**
**tocko_F2**	**TTTACCGGATTGATTCCATGA**	**Genotyping[17]**
**tocko_R3**	**CCAGTGAGAAGCTGGAGGAC**	**Genotyping[18]**

Notes

Primers in **bold** were used in the Kyoto lab. All other primers were used in the Uppsala lab.

1) Saint-Marcoux *et al.*, 2015
2) “cacc” is added to the 5’ end of the F primer for directional TOPO cloning in pENTR/D-TOPO (Thermofisher)
3) The gene ID for MpTUBβ2 is Mapoly0158s0010.1
4) MpPRR 3′ homologous arm
5) MpPRR 5′ homologous arm
6) MpRVE 3′ homologous arm
7) MpRVE 5′ homologous arm
8) MpTOC1 3′ homologous arm
9) MpTOC1 5′ homologous arm
10) Illustrated as primer set 1 in S8
11) Illustrated as primer set 2 in S8
12) Illustrated as primer set 3 in S8

13) Illustrated as primer set 1 in S9
14) Illustrated as primer set 2 in S9
15) Illustrated as primer set 3 in S9
16) Illustrated as primer set 1 in S10
17) Illustrated as primer set 2 in S10
18) Illustrated as primer set 3 in S10
19) Illustrated as primer set 2 in S8-10
20) Illustrated as primer set 3 in S8-10

Methods S1 Supplemental materials and methods describing sequence retrieval, sequence analysis and phylogenetic reconstruction.

Inventory of putative bryophyte and charophyte circadian clock genes

Homologs to *Arabidopsis thaliana* circadian clock genes were first identified in the liverwort *Marchantia polymorpha,* in the hornwort *Anthoceros agrestis* and in the charophyte *Klebsormidium flaccidum.* Initial gene family classification was supported by BLAST e-values, reciprocal BLAST searches to the Arabidopsis genome, characterization of conserved protein domains and by BLAST searches against the oneKP database (www.onekp.com).

Sequence retrieval

M. polymorpha genes were identified using Arabidopsis amino acid sequences as queries in tBLASTn searches in the publically available *M. polymorpha* genome v. 3.1 (Phytozome 11; https://phytozome.jgi.doe.gov). *A. agrestis* genes were likewise obtained searching the oneKP database (www.onekp.com). Algal gene sequences were retrieved from the publically available *K. flaccidum* genome V1.0 database (http://www.plantmorphogenesis.bio.titech.ac.jp/) and from transcriptome NGS data for the streptophyte algae *Cylindrocystis cushleckae* (Cc; Acc. No. ERR364373) and *Coleochaete irregularis.* (Ci; Acc. No. ERR364367*),* which were obtained from the NCBI short read archive (SRA). Sequences were assembled using Trinity r20131110 (Haas *et al.*, 2013), tBLASTn searches were performed and gene prediction was done using the web-based FGENESH+ program. Homologs were also retrieved from the following species: *A. thaliana*, *Oryza sativa*, *Selaginella moellendorffii*, *Picea abies* and *P. patens*. Arabidopsis sequences were downloaded from TAIR (www.arabidopsis.org). Genes of other species were identified by BLASTp searches with Arabidopsis queries against databases at JGI (http://www.jgi.doe.gov/), NCBI (http://ncbi.nlm.nih.gov) and plantGDB (http://www.plantgdb.org). To investigate the distribution of identified clock homologs among charophyte algae, hornworts, liverworts and mosses, BLAST searches were performed against the oneKP database (www.onekp.com). All previously identified clock genes as well as genes

identified in this study are listed with accession numbers in Supporting Information Table S1.

Sequence analysis and phylogenetic reconstruction

Conserved protein motifs and domains were identified using MEME Suite MAST (http://meme-suite.org; Bailey & Gribskov, 1998; Bailey *et al*., 2009), SMART (Schultz *et al*., 1998; Letunic *et al*., 2015), and NCBI CD-search (CDD v.3.14; Marchler-Bauer & Bryant, 2004; Marchler-Bauer *et al*., 2015). Amino acid sequences were aligned using the M-Coffee algorithm in T-Coffee (Notredame *et al*., 2000; Wallace *et al*., 2006). Alignments were filtered using Transitive Consistency Score (TCS) in the T-Coffee distribution (Chang *et al*., 2014) and are available in Supporting Information Fig. S1. Phylogenetic reconstructions were done using PhyML 3.0 (Guindon *et al*., 2010) and MrBayes 3.2.6 (Huelsenbeck & Ronquist, 2001; Ronquist *et al*., 2012). For MrBayes the substitution model used was decided individually for each alignment using Modelgenerator v.85 (Keane *et al*., 2006). The final phylograms were visualized and edited in TreeGraph2 (Stöver & Müller, 2010), where the Bayesian tree was used as reference tree on which both posterior probabilities and bootstrap proportion from PhyML were mapped. Branches resulting in conflicting topologies from the two methods were collapsed. Trees were rooted with charophyte sequences, except for the CCA1/LHY/RVE and PRR families that were midpoint rooted.

References for Supporting information

Bailey TL, Boden M, Buske FA, Frith M, Grant CE, Clementi L, Ren J, Li WW, Noble WS. **2009**. MEME SUITE: tools for motif discovery and searching. *Nucleic Acids Research* **37**: W202–W208.

Bailey TL, Gribskov M. **1998**. Methods and statistics for combining motif match scores. *Journal of Computational Biology* **5**: 211–221

Chang J-M, Di Tommaso P, Notredame C. **2014**. TCS: a new multiple sequence alignment reliability measure to estimate alignment accuracy and improve phylogenetic tree reconstruction. *Molecular Biology and Evolution* **31**: 1625–1637.

Galego L, Almeida J. **2002**. Role of *DIVARICATA* in the control of dorsoventral asymmetry in *Antirrhinum* flowers. *Genes & Development* **16**: 880–891.

Guindon S, Dufayard J-F, Lefort V, Anisimova M, Hordijk W, Gascuel O. **2010**. New algorithms and methods to estimate maximum-likelihood phylogenies: assessing the performance of PhyML 3.0. *Systematic Biology* **59**: 307–321.

Haas BJ, Papanicolaou A, Yassour M, Grabherr M, Blood PD, Bowden J, Couger MB, Eccles D, Li B, Lieber M *et al.* 2013. De novo transcript sequence reconstruction from RNA-seq using the Trinity platform for reference generation and analysis. *Nature Protocols* **8**: 1494–1512.

Huelsenbeck JP, Ronquist F. **2001**. MRBAYES: Bayesian inference of phylogenetic trees. *Bioinformatics (Oxford, England)* **17**: 754–755.

Keane TM, Creevey CJ, Pentony MM, Naughton TJ, Mclnerney JO. **2006**. Assessment of methods for amino acid matrix selection and their use on empirical data shows that ad hoc assumptions for choice of matrix are not justified. *BMC Evolutionary Biology* **6**: 29.

Letunic I, Doerks T, Bork P. **2015**. SMART: recent updates, new developments and status in 2015. *Nucleic Acids Research* **43**: D257–D260.

Marchler-Bauer A, Bryant SH. **2004**. CD-Search: protein domain annotations on the fly. *Nucleic Acids Research* **32**: W327–W331.

Marchler-Bauer A, Derbyshire MK, Gonzales NR, Lu S, Chitsaz F, Geer LY, Geer RC, He J, Gwadz M, Hurwitz DI *et al.* 2015. CDD: NCBI's conserved domain database. *Nucleic Acids Research* **43**: D222–D226.

Notredame C, Higgins DG, Heringa J. **2000**. T-Coffee: a novel method for fast and accurate multiple sequence alignment. *Journal of Molecular Biology* **302**: 205–217.

Ronquist F, Teslenko M, van der Mark P, Ayres DL, Darling A, Höhna S, Larget B, Liu L, Suchard MA, Huelsenbeck JP. **2012**. MrBayes 3.2: efficient Bayesian phylogenetic inference and model choice across a large model space. *Systematic Biology* **61**: 539–542.

Saint-Marcoux D, Proust H, Dolan L, Langdale JA. 2015. Identification of reference genes for real-time quantitative PCR experiments in the liverwort *Marchantia polymorpha*. *PLoS ONE* **10**: e0118678.

Schultz J, Milpetz F, Bork P, Ponting CP. **1998**. SMART, a simple modular architecture research tool: identification of signaling domains. *Proceedings of the National Academy of Sciences, USA* **95**: 5857–5864.

Stöver BC, Müller KF. 2010. TreeGraph 2**:** Combining and visualizing evidence from different phylogenetic analyses. *BMC Bioinformatics* **11:** 7.

Wallace IM, O'Sullivan O, Higgins DG, Notredame C. **2006**. M-Coffee: combining multiple sequence alignment methods with T-Coffee. *Nucleic Acids Research* **34**: 1692–1699.
